# Carbon Nanomaterials Embedded in Conductive Polymers: A State of the Art

**DOI:** 10.3390/polym13050745

**Published:** 2021-02-27

**Authors:** I. Jénnifer Gómez, Manuel Vázquez Sulleiro, Daniele Mantione, Nuria Alegret

**Affiliations:** 1 Department of Condensed Matter Physics, Faculty of Science, Masaryk University, 61137 Brno, Czech Republic; gomez.perez@ceitec.muni.cz; 2 IMDEA Nanociencia, Ciudad Universitaria de Cantoblanco, Faraday 9, 28049 Madrid, Spain; manuel.vazquez@imdea.org; 3 Laboratoire de Chimie des Polymères Organiques (LCPO-UMR 5629), Université de Bordeaux, Bordeaux INP, CNRS F, 33607 Pessac, France; 4 POLYMAT and Departamento de Química Aplicada, University of the Basque Country, UPV/EHU, 20018 Donostia-San Sebastián, Spain

**Keywords:** conjugated polymers, poly(3,4-ethylenedioxythiophene), polypyrrole, polyaniline, carbon nanotubes, graphene, carbon dots

## Abstract

Carbon nanomaterials are at the forefront of the newest technologies of the third millennium, and together with conductive polymers, represent a vast area of indispensable knowledge for developing the devices of tomorrow. This review focusses on the most recent advances in the field of conductive nanotechnology, which combines the properties of carbon nanomaterials with conjugated polymers. Hybrid materials resulting from the embedding of carbon nanotubes, carbon dots and graphene derivatives are taken into consideration and fully explored, with discussion of the most recent literature. An introduction into the three most widely used conductive polymers and a final section about the most recent biological results obtained using carbon nanotube hybrids will complete this overview of these innovative and beyond belief materials.

## 1. Introduction of Conductive Polymers

*What is plastic made of?* This simple question could have the same answer as *Why was I born?* Or *What is the meaning of life?* The answers are complicated. Actually, they are very complicated; not in terms of the real science behind them, but taking into account our background. When we think of *plastic*, some clear examples come to mind, such as water bottles, plastic glass or a shopping bag; it is more uncommon to think of textiles, adhesives or our eyeglasses. The six examples we have given here are from six different plastics, or better said, six different polymers. This is the term that chemists use to define all this family and even more. Etymologically the word *polymer* comes from Greek origins and is formed by two parts: *poly-* meaning many, and *-meros* meaning parts. Therefore, from the chemical point of view, everything that is composed of many (almost) identical molecules that are connected is a polymer, and thus, the initial question can be answered as: “…it depends which plastic are we talking about”. In recent years many new polymers have been discovered and used for various applications: biomedical, energy, environmental, etc. Among them, conductive polymers (CP) represent one of the most peculiar and revolutionary types of polymer. In fact, their discovery passed un-noticed and their boom came with the new millennium, when in 2000 a Nobel prize was given to this discovery which was made in the 1970s. From then on, the literature around these materials has been growing exponentially up to today. Conjugated polymers buck the trend as they conduct electricity and heat, two properties that the “conventional plastics” do not have. Hereafter, the three main conductive polymers, i.e., polyaniline (PANI), polypyrrole (PPy) and poly(3,4-ethylenedioxythiophene) (PEDOT), are considered, together with their synthesis, characterizations and principal properties. These three systems represent the most used CPs, thanks to their high stability, ease of handling and synthesis, as well as their low price.

### 1.1. Polyaniline (PANI)

This polymer started the conductive polymers’ story in the 1862 when H. Letheby, a chemistry professor at the London Hospital, tried to study two cases of fatal poisoning by nitrobenzene [1]. The professor electropolymerized aniline sulfate by varying the synthetic conditions until arriving at a black slurry. The only further observation made by him was that the reduced colorless state turns into a deep color when it is oxidized. The story of PANI commenced from 1968, when, in Paris, the group led by Buvet started to relate this polymeric chain to electronical behavior [2]. All in all, the actual rise of PANI came in the 1980s when many research groups began to shed light upon this topic [3,4,5,6].

**Figure 1:**
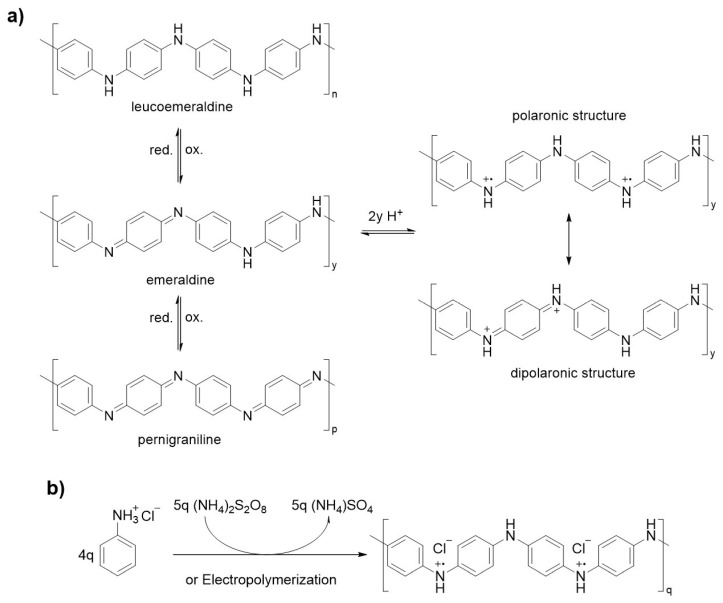
Skeletal formulas of polyaniline in different oxidation states and their resonance, (**a**) leucoemeraldine, (**b**) Oxidative chemical polymerization.

As shown in Figure 1, the base structure of PANI has a generalized composition, formed by alternating oxidized and reduced moieties, both equally present; this is called emeraldine. The oxidation state may vary, as does the names of these molecules: the most reduced (Figure 1a) is called leucoemeraldine, and the most oxidized pernigraniline. The protonated Emeraldine form is the most common form, green in color and with a conductivity around 10 S/cm, the non-protonated form has a blue color and is not conductive. PANI is used in a large variety of applications, from energy storage and conversion to biosensing applications, through to being used in sensors and supercapacitors. PANI’s physical characteristics change depending on the application, i.e., in the form of a powder, fiber, or colloid dispersion, etc. [7,8,9,10,11,12].

#### 1.1.1. Synthesis

PANI is generally prepared via oxidative polymerization or via electropolymerization; anyhow, it is worth mentioning, that enzymatic, ultrasound and ionizing radiation polymerization routes are also possible [13,14,15].

Oxidative chemical polymerization consists of a solution of aniline hydrochloride with a persulfate as the oxidant (Figure 1b). The reaction proceeds in water at room temperature in about 10 min and the product precipitates. The reaction is highly exothermic and the color changes from the colorless–greenish appearance, of the aniline hydrochloride, to deep blue, indicating the formation of oligomers. The viscosity increases during the reaction too, from a watery solution to a viscous dark paste. The mechanism, as shown in Figure 1b, occurs through the formation of a radical and an excess of oxidant is needed to ensure the presence of polarons in the PANI backbone. The final conductivity of the material is affected by many factors, among which, the reaction conditions represent a considerable part; however, an indicative value of 10 S/cm can be fixed as the average protonated emeraldine reference.

Electropolymerized PANI materials are prepared in a similar manner as the chemical ones, using an acid solution of aniline and cyclovoltammetry of chronoamperometry techniques. In this case, the acid acts as the electrolyte and the resulting product will have only the anion of the acid as a cationic balance on the chain. The polymerization occurs between 0.1 and at 0.82 V vs. Ag/AgCl electrode and it can be observed an increase in the voltammogram can be observed due to the deposition of the conductive layer and the oxidation of the conductive polymer [16]. The two peaks correspond to the first oxidation of a dimeric form and then the full oxidation to pernigraniline [17,18,19].

#### 1.1.2. Characterization

The most common form of analysis, used for almost all the conductive polymeric materials, is the ultraviolet–visible–near infra-red spectroscopy (UV–Vis–NIR) method. PANI presents the typical signals of a conjugated backbone around 300 nm due to the π–π* transitions, and at around 600 nm, a signal is attributed to the benzenoid-quinoid ring transition absorption (HOMO of the benzenoid ring to LUMO of the quinoid ring) [20,21,22]. Upon doping, via an acidic media, the intensity of the absorption of the polaron relative signal around 600 nm decreases and partially shifts to 450 nm, at the same time, a broad band of dipolarons become visible up to 950 nm [23].

FT-IR is another non-destructive useful technique to characterize CPs, including PANI. Even if not exhaustive, it can provide an indication of success in the synthesis. The spectrum is typically not explicit below 2000 cm^−1^, as all the spectra at higher wavenumbers are saturated in terms of absorption of the aliphatic/aromatic backbone. At around 1550 cm^−1^, quinone and phenyl bending is visible, while carbon–nitrogen stretching is detected at 1370 cm^−1^. The conductive framework is visible at around 1250 and 1150 cm^−1^, with the bands being relative to the protonated state [6,24].

Size exclusion chromatography (SEC) analysis is possible for PANI, in contrast to the expected difficulties relative to the highly charged polymeric chain. *N*-methylpyrrolidone (NMP) represents the most common solvent employed in this technique, coupled with soluble salts, such as lithium bromide (LiBr) or lithium tetrafluoro borate (LiBF_4_) [25,26]. Although the molecular chain variation depends on the different synthetic conditions used, the average in the literature is about 55 kDa polymer mass for around 600 aniline units. Although the polydispersity indexes are narrow in the electrochemical synthesis, PANI normally presents a broad range of mass with a dispersity (Đ or DPI) of 2–7 [25]. 

### 1.2. Polypyrrole (PPy)

The birth of polypyrrole (PPy) occurred around the end of the 1960s, although the first reference to PPy dates further back to 1919 [27]. The first scientific paper talking about a black slurry from the electropolymerization of pyrrole dates back to 1968, with the first paper that focused specifically on pyrrole dating back to 1973 [28,29]. Anyhow, the electropolymerization of PPy, together with the possibility of having a conductive material, had to wait until 1979 [30]. Since then, many studies have regarded this polymer, enunciating its stability in air, high conductivity and the ease of obtaining it [31,32,33,34,35]. Nowadays PPy is used in many fields, including in solar cells, biomedical applications, and energy storage, but its two main applications are in electronic devices and sensors [36,37,38,39,40]. The physical aspects of PPy are variable depending on the synthetic route used to obtain it and the final application; films, nanospheres, and nanowires are some examples of the possible final structures of PPy [32]. 

#### 1.2.1. Synthesis

**Figure 2:**
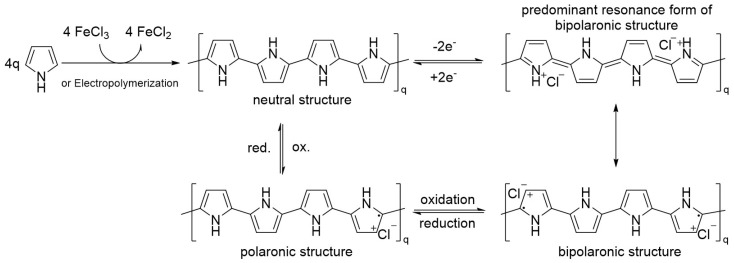
Pyrrole polymerization reaction, further redox equilibria and their resonance.

PPy is generally prepared via oxidative polymerization or via electropolymerization; although the mechanism is not fully understood, a radically oxidative coupling pathway seems the most probable explanation. The neutral structure, shown in Figure 2, is not conductive, has poor mechanical properties and presents a light yellowish color, while the polaronic or bipolaronic structure—the oxidized form—can reach conductivities of (10^1^–10^2^) S/cm and appears darker, tending display green or blue colors [41]. The stability under air is high: a doped film can last days without any changes and the polymer is stable up to 150–300 °C, while the reduced form is relatively stable under an inert atmosphere but is not stable under air, thus, it degrades and loses the redox properties [42].

Chemical oxidative polymerization runs smoothly at room temperature and is feasible even for a non-chemist user; for PANI, this is completed, on average, in 10 min. As common oxidant salts, iron (III) and copper (II) can be used in water, in addition to a large variety of organic solvents such as benzene, alcohols, halogenated solvents, dimethylformamide etc. [43,44,45]. The oxidant must be used in stoichiometric excess; 2.5 equivalents is the average amount reported in the literature: each unit of pyrrole needs two electrons to be polymerized, plus another excess to form the polarons. The final conductivity is also affected, in this case, by many factors: if, on average, we report a conductivity of 10^1^–10^2^ S/cm and 25% of positive nitrogen atoms, this can be pushed way higher, to around 2000 S/cm [46]. 

Electropolymerization of PPy has the added value of directly creating a film up to the electrode or the desired object. The thickness of the film can be adjusted depending on the cyclic voltammetry cycles and the nature of the electrolyte and solvent. PPy can be electropolymerized in water, acetonitrile, dimethylformamide, dichloromethane, and by using a large palette of electrolytes such as tetrafluoroborate, perchlorate, tosylate, hexafluorophosphate of alkaline or alkaline earth metals [33,47,48]. PPy starts to polymerize at 0.35 V in a water solution, or at around 0.65 V in organic media (vs. Ag/AgCl), forming a colored film: either dark green in the oxidized state or yellowish in the reduced state. The thickness of the film is controllable by the time of application of the anodic potential. The film formed at the cathodic potential becomes reduced and is insulating, with the possibility to swipe up to the solvent window. Instead, potentials up to 0.65–1 V could result in an irreversible over-oxidation and degradation of the films, with losses in conductivity and mechanical properties [49].

#### 1.2.2. Characterization

UV–Vis–NIR spectroscopy is a golden standard in PPy characterization. Similar to the rest of the conductive polymers, PPy presents an absorption peak around 400 nm due to the π–π* transitions, and a bipolaronic absorption at around 850 nm and across all the NIR, up to 1500 nm. In this case, the signal of the bipolaron is closer to the visible than in the case of PANI or PEDOT and, as in the other cases, the high absorption is directly related to the high conductivity of the material. The intensity of this band is proportional to the doping level of the material [50,51]. A band at around 500 nm, instead, is characteristic of undoped/reduced material, due to transition from the valence band to the polaron/bipolaron level. In the case of PPy, the difference between the polaron and bipolaron is not as defined as in PEDOT, and the literature is limiting in terms of reports on the differences between doped and undoped materials.

The FT-IR spectra of PPy results are more understandable compared to those for PANI or PEDOT, showing the stretching vibration of C=C at 1550 cm^−1^ and that of C–N at 1350 cm^−1^. The bending signals of C–N are clearly visible at 1150 and 1050 cm^−1^ for the in-plane deformation and at around 900 cm^−1^ for the out-plane [50,51,52,53,54,55].

Raman spectroscopy presents a consistent part of the PPy characterization literature as this method provides extremely precise results by comparing the doped dicationic form and the neutral undoped form. Excitations of 514, 633 and 780 nm are most commonly present in the literature, with this having both pros and cons: the short wavelength penetrates through the material, so could be affected by underlayers; it is close to the minimum absorption level of PPy, which could be detrimental to the resolution, however, as this is far from the energy of polarons and bipolarons, the neutral form can be better observed. In addition, the most common signals include: 1550 cm^−1^ C=C stretching, 1320 cm^−1^ C–C stretching, 1210 cm^−1^ N–H bending, 1030 and 880 cm^−1^ C–H bending [56].

Size exclusion chromatography (SEC) studies of PPy are not present in the literature as the solubility of the polymer in common laboratory solvents is low. However, the chain length can be calculated as it is correlated to the absorbance maximum, and this has been demonstrated to be around 40 units, or rather, a molecular mass of about 2500 Da [57,58,59]. MALDI-TOF analysis in positive mode confirmed this calculation, and, even if not numerous, an average of 20–30 units has been determined as the chain length [60,61,62].

### 1.3. Poly(3,4-ethylenedioxythiophene) PEDOT

Chronologically, PEDOT emerged last in the timeline of conductive polymers. Even though polythiophenes were studied in the 1960s, and the conductivity of oligothiophene rings was known about in 1982 [63,64], PEDOT was still discovered afterwards. Many researchers have fought to modify the thiophene structure with side groups in order to achieve a semblance of stability, for example, as in the case of PANI or PPy [65,66,67]. It was clear that bare thiophene was not yielding results, and that substitutions at position three and four were a must [68,69,70,71,72,73]. One of the discoveries of the century was made by researchers from the company Bayer^®^ in Germany. A dioxolane ring was the key, and since this discovery in 1988, the scientific environment has all been using the same, air stable, water solution of PEDOT [74]. However, PEDOT itself was not processable and to obtain the nowadays commercially available water solution it had to be coupled with an anionic polymeric chain poly(styrenesulfonate) (PSS). This was the second achievement that represents a milestone in the word of conductive polymers: from then on, the era of PEDOT:PSS commenced. Commercialized with the name Baytron^®^ or Clevios^®^, PEDOT:PSS seems one of the most ubiquitous polymer ever. It is present in solar cells, capacitors, organic transistors, OLEDs, bioelectronic devices, sensors and more [75,76,77]. Among the other characteristics, shared with others conductive polymers, such as stability in air, resistance to high temperatures and good mechanical properties; PEDOT:PSS is the most processable, user friendly, and economical. Macroscopically, PEDOT is a dark blue color when in suspension in water, and in order for it to be stable and processable, it must be coupled with a water-soluble anionic chain, such as PSS. In its solid state, via methods such as electropolymerization, in situ chemical polymerization or vapor phase polymerization, PEDOT can be obtained without the polymeric PSS, yielding a blue/green, either dark or light film. Other forms of PEDOT or PEDOT:PSS can also be achieved, such as nanospheres, nanotubes, fibers, coatings, etc., demonstrating its great versatility [78,79,80].

#### 1.3.1. Synthesis

Contrary to other CPs, PEDOT is not widely synthesized. The commercial solution sold by H.C. Starck—taken over by Heraeus and other major chemical sellers—is extremely stable and improved (over indium tin oxide, ITO, polyethylene terephthalate (PET), and others) solutions of PEDOT:PSS or films can be purchased cheaply. This represents a stumbling block: not only it is not encouraging for the development of research, but also because, after years of industrial improvements, these suspensions result in far more stable, more conductive and more performant solutions—in term of physicochemical properties—than homemade versions. All in all, PEDOT can be synthesized, as we reported, through many pathways and, among them, the two most common are: chemical oxidative polymerization, either in a water suspension or in an organic solvent (in situ), and electrochemical polymerization. The final conductivity will significantly vary from 10^−2^ to 10^3^ S/cm depending on the method, the solvents, the additives and the level of oxidation of the final material. It is worth mentioning, that commercial suspensions of PEDOT:PSS can vary from 10^−2^ to 1 S/cm for a bare spin coated non-post-threated film [81].

**Figure 3:**
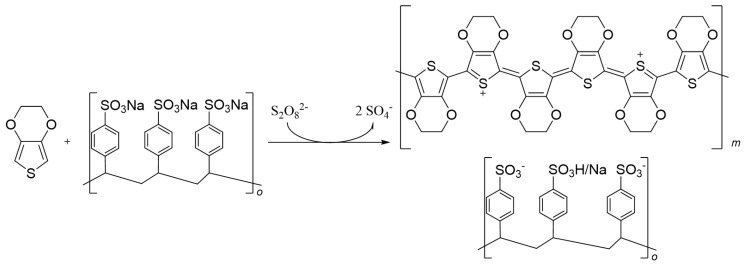
(3,4-ethylenedioxythiophene) (EDOT) polymerization reaction in presence of poly(styrenesulfonate) (PSS).

As shown in Figure 3, PEDOT can be obtained via chemical oxidation polymerization. Commonly, a persulfate oxidant in three molar equivalents is added to a water suspension of about 1% total solid content of EDOT and poly(styrene sulfonate) sodium salt (PSSNa), in a mass ratio of 1:2.5 EDOT:PSS. Iron (III), usually as FeCl_3_ or Fe(SO_4_)_3_, is added in a catalytic molar amount (0.05 eq). The kinetics of this reaction are slower than for other CPs, being completed at room temperature in about 8 h while stirring [82]. This suspension can be stored cold and is weakly stable. To avoid side reactions, a proper workup of the excess of oxidants via ultrafiltration or ion exchange resins is required [83,84,85].

In situ chemical and oxidative polymerizations are present in the literature and are more suitable for obtaining a highly homogeneous material, highly conductive (due to the lack of an insulating chain of PSS), but these materials are not processable afterwards. These syntheses normally consist of a highly concentrated solution of EDOT in an organic solvent (5–10% in mass), coupled with a polymerization retardant such as pyridine or imidazole that acts as a proton sink. The oxidant in this case is an over-stoichiometric amount of FeCl_3_ or Fe (III) p-toluene sulfonate. The mixture is heated and the polymerization is completed in several minutes [86,87,88].

The electropolymerization of PEDOT is one of the easiest methods, as the reaction conditions, in terms of solvents and electrolytes, are extremely flexible. Water, alcohols, acetonitrile, dimethylformamide (DMF), dichloromethane (DCM) are the principal solvents and KPF_6_, H_2_SO_4_, LiCl, NBu_4_PF_6_ and NaCl are the principal electrolytes present in the literature. The onset is at about 1.2 V vs. Ag/AgCl and the thickness is proportional to the time of the oxidative current applied. During cyclic voltammetry, it is possible to observe how the reduced/undoped film appears to be much less colored than the oxidized/doped one [89].

#### 1.3.2. Characterization

UV–Vis–NIR of PEDOT is, without a doubt, the most common form of characterization. The literature is rich in studies and interpretations of the obtained spectra. Older studies agreed that PEDOT has three major absorption bands: the neutral chain, which is the most reduced form, at around 600 nm; the radical cation of the polaron around 900 nm; and the dications or dipolarons after 1250 nm. Many studies have used this to prove the real doping of the material, as increased doping results in the polaron and dipolaron bands being higher [90]. Recently, a better interpretation has been achieved, stating that the absorptions do not belong to the two different species but the bands at 900 nm and 1250 nm belong to different orbital transitions that both, polarons and dipolarons, own.

FT-IR of PEDOT can be done either in solid form, as an electropolymerized film, in situ chemically polymerized or in solution as a PEDOT:PSS suspension. The spectra are not explicative, the region from 1700 cm^−1^ to higher wavenumbers is practically flat and does not provide much information either with or without PSS. The interesting region is at lower wavenumbers from 1700 to 800 cm^−1^. At 1650 and 1500 cm^−1^, two peaks are visible belonging to the C=C stretching of the benzene rings of PSS and of the thiophene ring, respectively. Carbon–oxygen stretching is visible at 1150 and 1100 cm^−1^. At 1350 cm^−1^, single bond carbon stretching appears, at 1200 and 1050 cm^−1^ the sulfur–oxygen double bond appears, and at 1150 cm^−1^ the single sulfur–oxygen bond appears, i.e., either of the sulfonate groups or the possible sulfate residues of the oxidant. The signals at 980, 930, 840, and 691 cm^−1^ belong to carbon–sulfur stretching [91,92,93].

Gel permeation chromatography (GPC) or SEC, in this case, is not possible due to the negligible solubility in most common laboratory GPC compatible solvents and the high charge of the polymeric chain; nonetheless, only a few examples of mass spectrometry using MALDI techniques are available in the literature [94,95]. It is worthy to mention, that since the radical nuclear magnetic resonance is not possible on bare PEDOT, its molecular weight remains unclear. Indicatively, more soluble EDOT derivates have been polymerized and analyzed via GPC, giving a degree of polymerization of about 10–60 units [96,97,98].

## 2. The Allotropic Nanoforms of Carbon

Nature has provided us with many different allotropic forms of carbon. This element, apart from being the most essential and common element found in nature, is the one that provides the highest number of different structures. The main reason for this is its capacity to form lattices through bonds using sp^2^ and sp^3^ hybrid orbitals. The most widely known allotropes of carbon are diamond—where all carbon atoms are found in a sp^3^ hybridization and disposed in a tetrahedral structure—and graphite, with all carbon atoms in a sp^2^ hybridization forming a planar structure. 

All allotropic forms of carbon that have a lateral size in the range of nanometers (1–100 nm) are referred to as carbon nanomaterials, and their discovery and evolution runs hand-in-hand with the evolution of the field of nanotechnology. The third allotrope of carbon and the first carbon nanomaterial was discovered in 1985, when Harold W. Kroto, Robert F. Curl and Richard E. Smalley purified a molecule containing 60 atoms of C arranged in a “soccer-ball” fashion, which was named *Buckminsterfullerene* [99]. Fullerenes are a family of spherical molecules, in which carbon atoms are bonded together through pyramidalyzed orbitals between sp^2^-sp^3^ hybridization. This milestone paved the way for further discoveries and in 1990, a tubular shaped elongated form of a fullerene was envisioned. Experimental evidence of this new carbon allotrope was produced in japan by Iijima in 1991, and after two years, the same group managed to synthetize and isolate the first single-wall carbon nanotubes (SWCNT) [100,101].

Later in 2004, mechanical exfoliation of graphite led to the discovery of graphene. This new material is formed by an atom thick single layer of graphite, where the sp^2^ hybridized carbon atoms are arranged in a honeycomb structure [102]. The impact of this discovery on the scientific community and eventually on everyday life, led to a Nobel price being awarded to its discoverers, just six years after its discovery. Carbon dots (CDs) are the youngest members of the carbon family, serendipitously discovered in 2004 by Scrivens and co-workers during the purification and separation of SWCNT [103]. It was not until 2006 that they received their first name as “carbon quantum dots” and appeared in the scientific community as new fluorescent carbon nanoparticles [104]. Typically, CDs are quasispherical nanoparticles with sizes below 10 nm and contain various functional groups, such as carboxylic acids and amines. Furthermore, CDs synthesis procedures are easy, fast and cheap [105,106]. Given all their amazing properties, it is not surprising that these new fluorescent carbon nanoparticles are considered highly promising nanomaterials for several applications [105,106,107,108]. 

Herein, we summarize the most recent advances in the field of conductive nanotechnology, which combines the properties of carbon nanomaterials with conjugated polymers. We focus on the hybrid materials resulting from the embedding of CNT, graphene derivatives and CDs with CPs. We also discuss how the combination of carbon nanomaterials (CNM) with CP improves the thermal, electrical and mechanical properties of the resulting hybrid materials. It is worth mentioning, that the fabrication and characterization approaches for these hybrid systems are quite similar. Therefore, against the usual timeline flow, we are going to take the reverse path and focus on the current progress in each field. In this way, we start with the most recent hybrid materials, i.e., CD/CP composites, which are in their infancy, to examine the basic production and evaluation approaches, followed by G/CP composites, which are at a slightly more advanced stage, to review the most common fabrication approaches and characterization techniques, and finish with the big brother, CNT/CP, which has been vastly explored and is in an advanced stage of research. We provide a special focus on the fabrication of the 3D architectures achieved and their applications. 

## 3. Carbon Dots and CP

**Figure 4:**
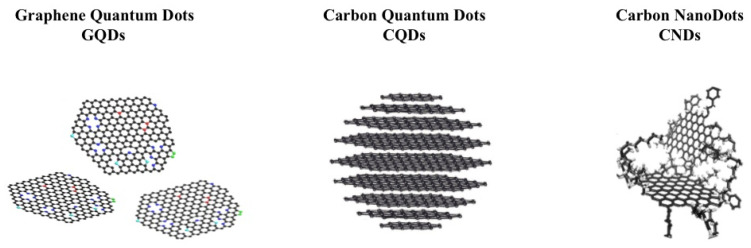
Illustration of the types of CDs considered. Carbon quantum dots (CQDs), graphene quantum dots (GQDs) and carbon nanodots (CNDs).

Carbon-based dots (CDs) are recognized as a class of carbon materials with unique optical properties, similar to traditional semiconductor quantum dots (QDs) [107,108]. Furthermore, CDs present high biocompatibility and lower toxicity than inorganic dots, making them a promising green alternative material for bioapplications, among other applications [106,108]. CDs have been widely studied since their discovery and [103], meanwhile, polymers have increasingly become an essential tool for modifying CDs to provide a polymeric matrix to tune and enhance their properties. CDs can be synthesized using simple, fast, and low-cost approaches [105,106]. Herein, in the following sections, the main three types of fluorescent CDs will be considered and these are represented in Figure 4: carbon quantum dots (CQDs), graphene quantum dots (GQDs) and carbon nanodots (CNDs) [109]. We summarize the use of CPs in the modification of CDs, including their fabrication, characterization, properties, and the applications of these composites in various fields.

### 3.1. Fabrication and Processing

**Figure 5:**
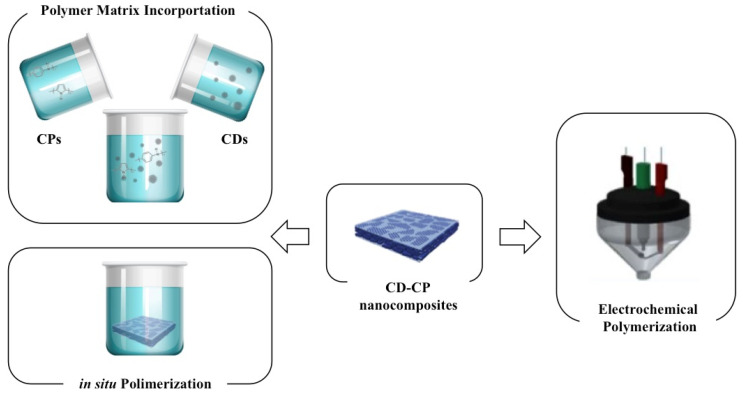
General scheme of the fabrication and processing methods of carbon dot–conductive polymer (CD–CP) nanocomposites.

CDs embedded with CPs can be prepared via polymerization, polymer matrix incorporation, electrochemical polymerization and deposition or co-deposition. A summary is shown in Figure 5 and a detailed discussion of each process is presented below.

#### 3.1.1. Polymerization and Polymer Matrix Incorporation 

The most common and cost-effective methods to incorporate CDs within a polymer matrix are based on in situ polymerization and/or polymerization in a solution of the conductive monomer in the presence of an oxidant and the carbon-related dots. Nevertheless, the main drawback when using these methods, is that they do not achieve the same level of homogeneity and integrity on the final nanocomposite as the produced by electrochemical polymerization.

**Figure 6:**
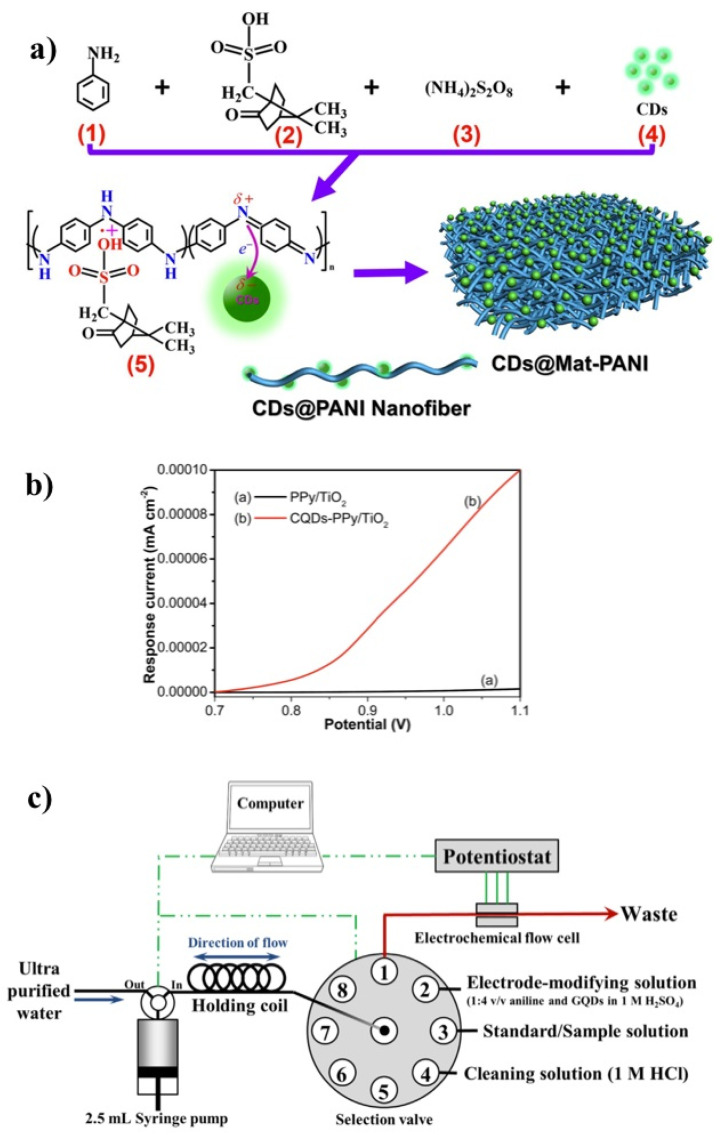
(**a**) Schematic diagram of the synthesis procedure of CDs@Mat-PANI. Adapted from reference [110] (**b**) Response current curves of PPy/TiO_2_ and CQDs–PPy/TiO_2_ nanotube hybrid in a regular pulse voltammetry deposition process. (**c**) Illustration of the Auto-Pret system coupled with electrochemical detection for online modification of the screen-printed carbon electrode (SPCE) with polyaniline (PANI)-GQD and the determination of Cr. Adapted with permission from ref. [116,117]. Copyright Royal Society of Chemistry (2015) and Elsevier (2016), respectively.

Polymerization of CPs, such as aniline and pyrrole, have been known to industries for about a century. Despite this, reports of CDs tailored with CP are scarcely found in the literature. For instance, the development of magnetic CD nanocomposites with PANI was achieved by in situ polymerization of a solution containing the monomer and ammonium persulfate (APS) as an initiator, in H_2_O, which served as a matrix [110]. Thus, aniline can be chemically oxidized into the polymer form, i.e., PANI, in an aqueous solution by various oxidants, as long as the oxidant is capable of withdrawing a proton from an aniline molecule without forming a strong co-ordination bond [111]. The matrix incorporation method was used to create, in situ, the nanocomposite, while the polymerization of PANI occurred. The CDs were attached to the CP through electrostatic interactions, forming the so-called CDs@Mat-PANI nanocomposite (Figure 6a) [110]. Similarly, CDs embedded in PPy nanocomposites (CDs-PPy) were prepared using CDs and Py in an acidic medium [112]. The nanocomposite was synthesized by combining an acidic Py solution to a new aqueous solution of CDs, with H_2_O_2_ as the initiator and matrix, which finally led to the polymerization of Py. In detail, the color of the reaction mixture gradually changed from brownish to black as polymerization of Py started to occur in the presence of the dots [112]. Xia et al. reported a similar procedure of nanocomposite fabrication. In that study, PPy and GQDs were successfully obtained through in situ polymerization of Py in the presence of a GQD suspension, where oxidative chemical polymerization of PY occurred thanks to the dots that served as a soft template [7]. 

Interestingly, CDs could be used as initiators of the polymerization of PANI and PPy from the monomers under UV light [113]. Recently, Gedanken and co-workers reported a one-pot synthesis of PANI and PPy using CDs and UV light that act as catalysts without any additional initiator [113]. 

#### 3.1.2. Electrochemical Polymerization and Deposition 

The electrochemical polymerization and deposition methods for the fabrication of conductive nanocomposites present several advantages compared to the processes mentioned above. Such advantages include direct, homogeneous, and controllable synthesis. One of the first attempts to electrochemically deposit CPs on CDs was made in 2013 by Li and co-workers [114]. The authors prepared a counter electrode that was based on the electrodeposition of GQDs doped with PPy onto F-doped tin oxide (FTO) glass. The GQDs used were synthesized via a chemical oxidation approach and then mixed with Py in different weight ratios. The polymer film synthesis was carried out via the electrochemical deposition method, using a deposition voltage of 0.8 V. Afterwards, the GQD-doped PPy films were washed using deionized water and finally dried at 80 °C under vacuum conditions [114]. According to the authors, the CP electrodeposition with a constant voltage could enable the connection of the polymerized film to the FTO substrate. In addition, GQDs have abundant oxygen groups that lead to a negative charge and, in contrast, PPy units have positive charges on their N. Therefore, GQDs and Py should interact well due to their opposite charges, and the dots can electrostatically absorb Py at nucleation sites to promote the PPy growth.

CDs and titanium were used for the deposition of PANI onto films [115], with a higher current density and more effective polymerization being found compared with PANI deposited on Pt electrodes. For instance, doped S-, N-GQDs and titanium electrodes were utilized for the formation of PANI composite films by in situ electrochemical polymerization [115]. In their work, the authors claimed that the deposition of CP on metals, such as titanium, always required a cleaning treatment of the substrate to remove natural oxides. Thus, before the electrochemical polymerization of aniline, the titanium electrodes were first mechanically polished, rinsed in distilled water, and finally chemically etched. Later, the electrochemical polymerization of aniline was conducted through cyclic voltammetry in an acidic solution containing aniline and GQDs. The PANI-GQD film formation and growth on the electrode surface was confirmed, as seen from its cyclic voltammogram. 

Apart from film formation, CPs and CDs have been used for the processing of nanotubes. In particular, a CQDs–PPy/TiO_2_ nanotube hybrid was fabricated by a controlled electrochemical polymerization method [116]. This consisted of using a three-electrode system where the TiO_2_ plays the role of the working electrode, Pt as the counter electrode, and Hg/Hg_2_Cl_2_ as the reference electrode. Then, a normal pulse voltammetry deposition method was optimized to synthesize the hybrid. Figure 6b displays the response current curves of the CQDs–PPy/TiO_2_ and PPy/TiO_2_ nanotube hybrid during the fabrication process. It can be observed that the electrochemical polymerization reaction of the Py monomer occurs predominately at an anodic potential above 0.8 V, as a quick enhancement of the response current was achieved when the electrode potential was further increased from 0.8 to 1.1 V (vs. Hg/Hg_2_Cl_2_). This behavior indicates that the presence of GQDs could effectively promote the electrochemical polymerization reaction, and thus, the continuous electrodeposition of PPy on the TiO_2_ nanotube array. 

The processing methodologies employed so far can be considered as easy and feasible, using—in most cases—a single compartment cell with a three-electrode system. In another study, electrochemical polymerization using PANI was also achieved by Chailapakul and co-workers using a novel approach [117]. PANI/GQDs were used to modify a screen-printed carbon electrode (SPCE) in a flow-based system. This was the first study that involved a fully computer-controlled methodology, with accurate synchronization between the system, named Auto-Pret, and the potentiostat (Figure 6b). First, an electrode-modifying solution was prepared, which contained an aniline monomer in an acidic medium and an aqueous solution of GQDs, previously designed by a bottom-up pyrolysis approach using citric acid [118]. The electrode-modifying solution was set up for online modification in the Auto-Pret system, where the SPCE surface was covered with the solution and followed by the electro-polymerization of aniline using cyclic voltammetry [117]. 

### 3.2. Characterization and Properties

The CD–CP composites combine the large pseudocapacitance of the CP with the unique optical properties of the CDs. Thus, analysis of the composition, morphology, electronic and fluorescent properties, and others, are essential to understand and guarantee their successful interaction. 

#### 3.2.1. Composition, Morphological and Physical Characterization and Properties

FT-IR is the most commonly used technique to determine the composition of the CD–CP composite through the vibrations of the bonds on the functional groups [119,120], while X-ray photoelectron spectroscopy (XPS) deals with the atomic composition of the nanocomposites [37,110]. RAMAN is mostly used for graphene-related dots (i.e., GQDs) modified with CPs, and gives the vibration of sp^2^ bonded carbon atoms in the 2D hexagonal lattice (1573 cm^−1^, G-peak) and the vibrations of carbon atoms with dangling bonds, related to the defects and disorders in structures in carbon materials (1342 cm^−1^, D-peak). The intensity ratio for the D-band and G-band (I_D_/I_G_ ratio) is widely used to evaluate the defect quantity in graphitic materials and the chemical elements present in the materials [121]. Additionally, X-ray diffraction (XRD) is commonly employed to confirm the π–π interactions between the CPs and CDs in the matrix [7,122]. 

Thermogravimetric analysis (TGA) investigates the thermal stability of the CDs–CPs [113], while it can also quantify the percentage of each specimen that conforms to the resulting nanocomposite [119]. The incorporation of CDs into the polymeric matrix can extraordinarily increase the thermal stability [123], which can be explained due to the presence of a large number of aromatic structures acting as a thermal barrier, and hence, preventing degradation. 

**Figure 7:**
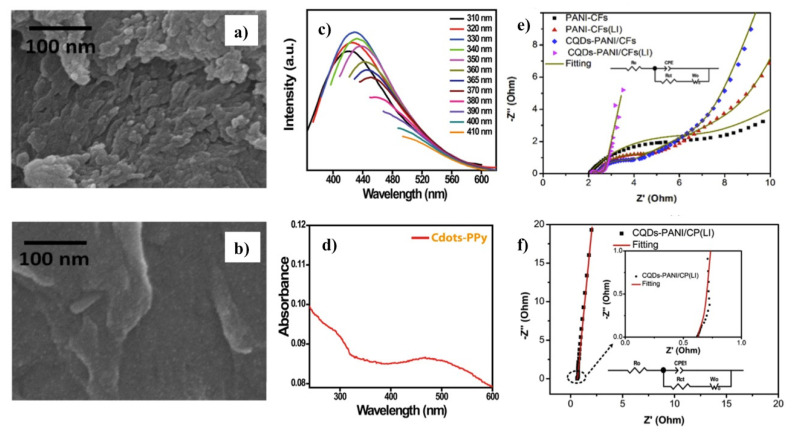
SEM images of (**a**) PPy@Cdots (1:1) and (**b**) PANI@Cdots (1:0.4) supercapacitor. (**c**) Emission spectra of Cdot-PPy composite. (**d**) UV–Vis absorption spectra of CD-PPy composite in water. (**e**) Nyquist plots and the equivalent circuit, (**f**) Nyquist plots of the supercapacitor based on CQDs-PANI/CP(LI) and the corresponding equivalent circuit. Reprinted with permission from reference [37,112,119,128]. Copyright Elsevier (2015, 2017) and American Chemical Society (2016, 2018).

Regarding the morphology and size of the CD–CP films, CPs such as PPy usually present chainlike structures, while PEDOT shows a grain-like architecture. Their electrodeposition, via a constant voltage, can enable the connection of the polymerized film to the substrate [114]. Several studies have shown that the introduction of CDs can electrostatically absorb the CPs as nucleation points to promote their growth, resulting in many “nano-islands” and a highly porous structure, as can be observed by scanning electron microscopy (SEM) [114,115,124]. In other cases, the addition of CDs promotes smoothing and flattening of the surface of CD–CP composites when compared to that of pristine polymers (Figure 7a,b) [119]. However, it is challenging to distinguish CD polymer matrixes by SEM because of their small size (less than 10 nm). Therefore, transmission electron microscopy (TEM) is more practical as it can show the small quasispherical carbon nanoparticles embedded in the polymer matrix [112]. Moreover, high-resolution TEM (HRTEM) can analyze the semicrystalline nature of the nanocomposite. The crystalline nature of the material can be established by selected area electron diffraction (SAED) measurement. Thus, HRTEM reveals the presence of fringes with lattice spacings from 0.35 to 0.28 nm, which correspond to interplanar distances and the face-to-face stacking of CPs with aromatic structures, such as PPy [112]. Additionally, a crystal lattice spacing around 0.20 nm can be observed by HRTEM, which could be related to the crystalline CDs [105]. Overall, atomic force microscopy (AFM) is in good agreement with SEM, and some reports suggest that the diameter of CD–CP structures varies in the range from 106 to 172 nm with an average diameter of 134 nm [121,125]. Nevertheless, it seems that the size is more dependent on the CP and crosslinking level [126,127]. 

#### 3.2.2. Optical Characterization and Properties

Conductive materials combined with carbon-related dots may bring many advantages, such as enhanced fluorescence intensity and sensitivity. The optical properties of these composites are characterized by fluorescence, UV–Vis absorption and UV–Vis diffuse reflectance spectroscopies, i.e., solid samples, which are extremely important for applications that include sensing, such as fluorescent biosensors. It is well known that CDs present unique optical properties, such as tunable fluorescence emission and excitation wavelength-dependency [105,106,107,108]. On the one hand, it is reasonable to expect the remaining excitation wavelength-dependance after CP coating, and most reports did indeed observe this behavior (Figure 7c) [37,112]. On the other hand, it has been observed that incorporating CPs into the hybrid nanocomposite leads to an increase in the fluorescence intensity, as was claimed by Shen and co-workers [37]. The authors presumed that such an increase might come from the surface passivation of the GQDs through an acid–base type interaction between the carboxylic groups of GQDs and the nitrogen atoms of PPy [37]. However, discrepancies regarding the quenching fluorescence effect when incorporating a CP have also been described. For example, Chattopadhyay et al. observed a reduction in the intensity after incorporating CPs [112], concluding that the main factor governing this phenomenon was the electron transfer through a non-radiative pathway between CDs and PPy. Consequently, a decrease in the fluorescence quantum yield (QY) of CD–PPy composites was found [112]. 

In addition, CD–CP composites present a broad optical absorption on the UV–Vis range in aqueous dispersion (Figure 7d). CDs showed their maximum absorption on the UV with a tail extending to the Vis range in aqueous solution [107,108,118]. Several reports have claimed that the introduction of CPs may produce broader and red-shifted signals compared to those of pristine polymers [112,123]. For instance, CD–PPy displayed broad absorption bands at 290 and 468 nm, respectively (Figure 7d). The first absorption peak around 290 nm was assigned to the π-π* transition band. The authors hypothesized that the second absorption band, from 350–600 nm, might be related to a bipolaron transition from the CPs and the n-π* transition band from the CDs, due to interaction between transition bands into the nanocomposite [112].

#### 3.2.3. Electronic or Electrochemical and Thermoelectric Characterization and Properties

The electrochemical performance of the synthesized polymer-carbon-based dot materials has been reviewed through cyclic voltammetry (CV), galvanostatic charge–discharge (GCD) and electrochemical impedance spectra (EIS) in a three-electrode system. In all the studies performed, whether using CQDs or GQDs, a small number of CDs enhanced the electrochemical performance significantly [7,116,128]. Moreover, the type and amount of dopant and/or CP present on the nanocomposite may also have an impact on the electrical properties of the system. For instance, Wu and co-workers studied the electrochemical behavior at different ratios of GQDs used within the matrix by CV [7]. The authors observed a relevant increase even at lower rates, from 344 F g^−1^ specific capacitance of pure PPy to 394 F g^−1^ PPy-GQDs (5:1). The highest specific capacitance of 485 F g^−1^ was reached using the 50:1 PPy to GQD ratio. Afterwards, when the mass ratio of PPy-GQD was higher than the optimal one, there were not enough templates for PPy growth. Therefore, a decreasing specific capacitance was detected. Notably, the authors noticed good cycling stability as well. These findings were associated with a reduction in the oxygen-containing groups in PPy-GQD composites during the electrochemical process and the defects in the GQD structure. 

Additionally, Xie et al. conducted EIS measurements to investigate the ionic diffusion and electron transfer in the PANI-based electrodes (CQDs-PANI) [128]. Figure 7e,f show the Nyquist plots and the equivalent circuit model, which includes the elements: ohm resistance (Ro); constant phase element (CPE1); charge transfer resistance (Rct); and the Warburg resistance (Wo). In all cases, an incomplete semicircle at the high-frequency region and linearity at low-frequency region were observed, where the authors suggest that there is a charge transfer resistance (Rct) at the electrode/solution interface. In addition, the curve in the low-frequency region is close to a vertical line, which means that the supercapacitor exhibited almost ideal capacitive behavior. After adding CQDs, the fitted Rct and Wo values were 0.8175 and 0.3521 U, respectively, which were much smaller than those of the pristine and control electrodes. Conclusively, such observations indicated that the CQD-PANI electrode possessed a high electrochemical performance and fast ionic diffusion in the electrode structure.

Du and co-workers demonstrated that the electrical properties could be enhanced even more if a TiO_2_ nanotube array is used during electrochemical polymerization as a working electrode [116]. They investigated the electrochemical capacitance through the GCD of CQD modified PPy/titania (CQD–PPy/TiO_2_) nanotubes. The authors calculated the specific capacitance using the following Equation (1):

(1) $$C_{m}=\frac{I\;\times \;\Delta t}{\Delta V\;\times \;m}$$

where *Cm* is the specific capacitance, *I* is the current of charge–discharge, Δ*t* is the discharge time, Δ*V* is the scanned potential window, and *m* is the mass of the active material in the electrode. The GCD curves obtained from the nanocomposites exhibited a straight line and had a very symmetrical nature, suggesting excellent reversible and fast ion doping.

PEDOT has recently been suggested as a thermoelectric material. Among CP, the commercially available solutions of PEDOT:PSS possess great potential in thermoelectric applications due to their high electrical and low thermal conductivity, in addition to their low-cost production. Nevertheless, their thermoelectric properties are not sufficient compared to their inorganic counterparts. A great deal of effort has been made to enhance PEDOT:PSS thermoelectric properties by increasing the charge transfer between PEDOT chains. Cheng et al. demonstrated that using GQDs onto PEDOT:PSS enhanced the thermoelectric properties of the final nanocomposite [124]. To help to assess the feasibility of PEDOT:PSS/GQDs, the electrical conductivity and Seebeck coefficient were analyzed in this work. The composites reached the highest Seebeck coefficients of more than 15 µV/K. It is well known that high-performance thermoelectric materials require a high Seebeck coefficient and electrical conductivity. Even though GQDs present a low electrical conductivity, adding a small amount of them onto the PEDOT:PSS brought a significant improvement in the electrical conductivity from 5475 to 6489 S/m. The authors ascribed the improvement to the strong interactions created on the nanocomposite, such as interactions between GQDs–PEDOT and GQDs–PSS, via π-π bonding chains and hydrophilic groups, which finally led to an increase in the PEDOT charge transfer for the decoupling and separation of its chains [124]. 

### 3.3. Applications

**Figure 8:**
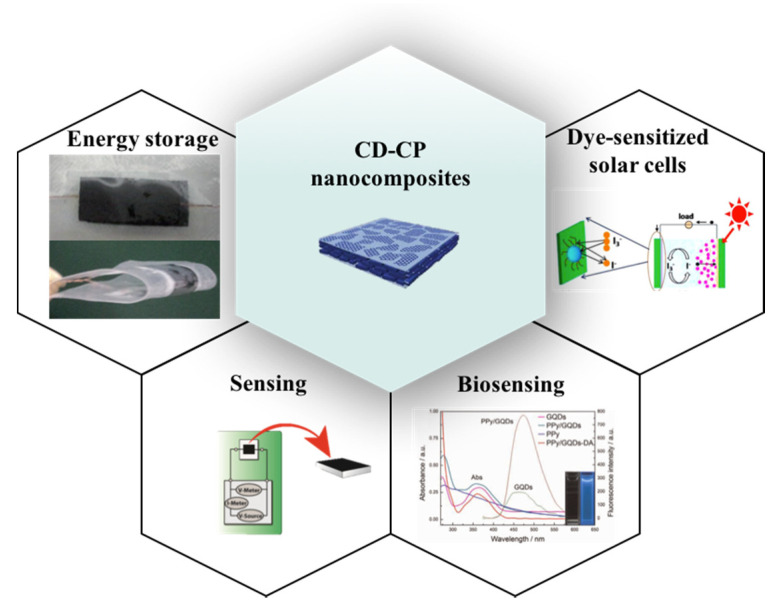
Applications where CD–CP composites have been applied. Adapted with permission from references [37,112,114,116], Copyright Elsevier (2015) and American Chemical Society (2013, 2016) and The Royal Society of Chemistry (2015).

Although the CD–CP field is still in its infancy and not many reports have yet been published, in this section, we present the current state-of-the-art applications of CD–CP composites and anticipate that these will exponentially increase in the coming years [129]. The unique properties of CDs combined with CPs have demonstrated outstanding potential for a wide range of applications and may change the landscape of various fields, such as energy storage, dye-sensitized solar cells, sensing and biomedical applications (Figure 8).

#### 3.3.1. Supercapacitors for Energy Storage Applications

Most of the published studies have involved the use of CD–CP composites designed as supercapacitors for energy storage applications [115,119,120,122]. Commonly, supercapacitors work under two energy storage mechanisms: the electrical double-layer capacitance (EDLC) stored via ion adsorption and the pseudocapacitance stored via fast surface redox reactions. Nearly all π-conjugated CP systems, such as PPy and PANI, have an adequate electronic charge flow and are most widely used for the latter-mentioned applications. Ideally, their excellent properties depend on the electrode materials, which should present high electrical conductivity, good stability, and exceptional capacity.

For instance, Goudarzirad’s team has developed an electrode based on PANI/GQD composites [115]. The group evaluated their potential as supercapacitors through CV. The cyclic voltammograms were recorded in 0.5 M H_2_SO_4_ electrolyte at the potential scan rate of 10 mV s^−1^ and at the potential range of −0.20 to +0.60 V. The shape of the voltammograms was more extensive than that of pristine PANI film electrodes in the same scan. The results obtained predict that the higher capacitance of PANI/GQD composites may be attributed to the larger surface area. Thus, increasing the interfacial surface between the nanostructures and the electrolytes leads to more fascinating reactions. Similarly, Xie and co-workers created a flexible solid-state supercapacitor with a promising application in flexible energy-related devices [128]. Additionally, Imae et al. reported capacitance enhancements by using CDs in CPs of more than double those shown for pristine polymers [119]. 

A CQD–PPy/TiO_2_ nanotube hybrid was fabricated as a supercapacitor electrode material by the controlled electrochemical synthesis route [116]. Next, Du and co-workers removed the TiO_2_ from the CQDs–PPy/TiO_2_ to create a flexible supercapacitor. Figure 8 shows images of a flexible CQD–PPy supercapacitor in both planar and bent states. In detail, the supercapacitor could be fully folded to 180º and exhibited a similar capacitance performance by comparing the integral areas of the CV curves. Furthermore, the shape of the CV curves indicates characteristic supercapacitor behavior, for example, conduction is attributed to the inner parts in the electrolyte not being able to sustain fast redox transitions at high scan rates [116]. 

#### 3.3.2. Dye-Sensitized Solar Cells

Dye-sensitized solar cells (DSSCs) present special features, such as simple design, low manufacturing costs and a high power conversion efficiency [130]. Therefore, it is not surprising that they are becoming an excellent alternative for substituting traditional solar cells in energy storage applications. DSSC devices consist of a dye-sensitized nanocrystalline-TiO_2_ photoanode, electrolyte-containing triiodide/iodide (I_3_^−^/I^−^) redox couple, and a counter electrode. However, the counter electrodes used up to now present some limitations, such as high-costs in the case of Pt, in addition to the volatile liquid used as the electrolyte often resulting in leakage and low chemical stability [131]. To overcome these drawbacks, incorporating nanocomposites based on CPs with prominent and tunable electrical features in organic electronics and CDs may enhance its photovoltaic performance. For instance, Li and co-workers developed a DSSCs device using a GQD-PPy film as the counter electrode [114]. Using 10% of GQDs doped onto the film resulted in a higher charge transfer rate toward the I_3_^−^/I^−^ redox couple. Additionally, the nanocomposite enhanced the power conversion efficiency up to 5.27%. The latest results were comparable to those of Pt and higher than pristine CPs. The GQD-doped PPy provides a promising candidate to replace Pt as an inexpensive counter electrode for DSSC devices.

#### 3.3.3. Sensing Applications

The exceptional optical properties of CDs have been utilized to detect a wide range of relevant molecules based on either turn-on or turn-off fluorescence emission mechanisms [106,108]. Despite the high fluorescence emission sensitivity, the high electrical conductivity of CPs is also attractive for CD nanocomposites to work as sensitive sensors, leading to improved detection sensitivity and selectivity [112,117].

##### Fluorescence Biosensors

In the last decade, CDs have attracted a great deal of interest in biomedical research communities. The dots harbor unique properties, making them a promising material for biomedical applications both in vitro and in vivo [105,107,108]. Furthermore, adding CPs as biofriendly polymers may revolutionize their biological potential, with a particular focus on biocompatibility, biodegradability and bioimaging [123,126,127]. Nevertheless, the bioapplications developed up to now are based on biomedical devices and, therefore, used ex vivo [37]. 

The Shen’s group developed a highly sensitive, simple and economically designed dopamine sensor based on PPy/GQDs [37]. The detection method of the biosensor relies on the turn-off system, i.e., quenching on the fluorescence emission, through a photo-induced electron transfer process, a strong electrostatic interaction between the electronegative part of PPy/GQDs composite and the electropositive part of a dopamine biomolecule. Moreover, hydrogen bonds between the amine groups and/or the hydroxyl groups in dopamine and the nitrogen-oxygen functional groups in PPy/GQDs are another force for detecting dopamine on the nanocomposite surface. Overall, all of these interactions led to the quenching of the fluorescence emission, and therefore, its detection. These sensing experiments were carried out in real samples, such as human serum and urine. It demonstrates that the sensor proposed by the Shen group is appropriate for practical applications related to the clinical determination of dopamine.

##### Electrical Conductivity Sensors

Reports based on CD–CPs nanocomposites for metal ion detection applications are scarcely found in the literature. Nevertheless, one report can be found online regarding a sensor for the determination of Cr (VI) using SPCE in an AutoPret system, coupled with electrochemical detection [117]. The proposed sensor was used for the detection of Cr (VI) in mineral drinking water and deteriorated Cr-plating samples. Furthermore, it could be used several times without any renewal processes and more than 90 samples per hour could be analyzed, with a detection limit as low as 0.097 mg L^−1^.

In 2016, the first sensor based on the conductivity of CDs was reported [112]. Pal et al. manufactured a conducting nanocomposite consisting of PPy-CDs (Figure 8). The conductive film was highly selective and sensitive toward picric acid, considered as a groundwater and soil pollutant. The detection of picric acid was based on the changes in the current–voltage (I−V) characteristic plots. Interestingly, the authors found concentration-dependent distinct differences on the current, which indicated a higher degree of protonation to the aromatic ring in the PPy backbone, leading to a higher charge carrier density. More analytes were assessed, even though the composite showed much higher sensitivity toward picric acid. It is worth mentioning here, that the detection limit for electron acceptor molecules was lower than those of electron donor molecules. 

## 4. Graphene Mixed with CP

Graphene, considered as a single layer of graphite, is one of the most recent additions to the allotrope carbon family. Since its discovery in 2004 by Novoselov and Geim, and the Nobel Prize for its discovery later awarded in 2010, the number of graphene publications has increased considerably [132]. Its discovery involved the start of a new generation of materials with multiple properties and applications: the 2D materials. The properties of this single layer of atoms arranged in a honeycomb lattice are numerous and diverse, from its mechanical properties due to its lightness and elasticity [133], to the electrical behavior that comes from its structure with excellent charge mobility [134]. Additionally, the high optical transparency [135], and the higher thermal conductivity of this material [136] makes it this an ideal candidate for a large variety of applications, such as field-effect transistors [137], sensors [138], supercapacitors [139], and hydrogen storage [140,141]. 

The term graphene means a hexagonal arrangement of sp^2^ carbon atoms in a single atom-thick layer [142]. It has been used to inaccurately describe many different types of graphene-based materials, such as several layer-thick graphene (FLG), graphene oxide (GO) reduced graphene oxide (rGO), graphene ribbons and graphene nanosheets. An accurate model of the graphene nomenclature and classification was defined by Wick et al., as well as a description of how the properties of these materials change depending on their composition, making them suitable for different applications [143]. 

In the last decade, a great deal of effort has been made to introduce graphene into several devices to replace, improve, or reinforce its application [144,145,146]. One of the challenges is to produce optimum graphene, i.e., with the best electrical properties, and even though many are trying, only few have succeeded [147]. The incorporation of graphene within CP, results in the generation of new unique polymers with enhanced properties and a wide range of applications. For this reason, several graphene-based materials embedded in conductive polymers are discussed in this section. 

### 4.1. Fabrication and Processing

The integration of graphene with polymeric conductive chains is a crucial step for improving the thermal, electrical or mechanical properties of the composites. The fabrication involves the interfacial bonding and distribution of the matrix with graphene. In many cases, small amounts of graphene are enough to improve the properties of the new composites [148]. There are different methods for the fabrication and processing of CP/graphene composites, as we will discuss below.

#### 4.1.1. In Situ Production

The most widely used technique for the fabrication of new compounds based on graphene and CP is the in situ polymerization of the monomer or the pre-polymer in the presence of the 2D material. The polymerization process should integrate the graphene flakes homogenously within its matrix. An essential step in the fabrication of graphene embedded on CP is the mixing procedure, where good dispersions and uniformity between them are crucial for the fabrication of reliable composites [149,150,151]. This connection can be performed through a covalent anchor or supramolecular interaction. In the covalent modification, the surface of the material should be perturbed, disrupting the aromatic character of the graphene, although the new moieties introduced onto the surface act as anchor points and enrich the assembly of the CP [152]. On the other hand, in the supramolecular fabrication, the formation of the composites does not alter the main properties of the graphene or CPs, and they are easier to manufacture [153]. For instance, Zhu and co-workers introduced an in situ polymerization of a PPy/GO film [154]. The authors showed how the nanocomposite has a noticeable improvement in corrosion protection. 

The preparation of graphene and a conductive polymer through two-step functionalization, mixing, and heating of the polymer, without the use of any additive as a catalyst acid or oxidant, was developed by Rizwan and co-workers [155]. The functional group introduced in the GO surface acts as a strong linker. Moreover, the new composite showed an excellent electrochemical performance, making this new graphene-polymer suitable as a supercapacitor electrode material with good cycling stability. The synergistic interaction between the polymer and GO allows for fast electron transfer. This methodology showed how the covalent bond crosslinker enhances the interaction in the graphene–polymer composites. Bose et al. identified a new composite made from the in situ polymerization of PPy with GO and chemical reduction with hydrazine. The composites with a higher number of sp^2^ domains showed an elevated value of conductivity; this behavior was attributed to the high aspect ratio and surface area of the graphene nanosheet embedded on the PPy matrix. The difference before and after the reduction was confirmed by XPS—with a decrease in the C/O ratio being observed, XRD Raman and FTIR spectroscopic analyses [156].

**Figure 9:**
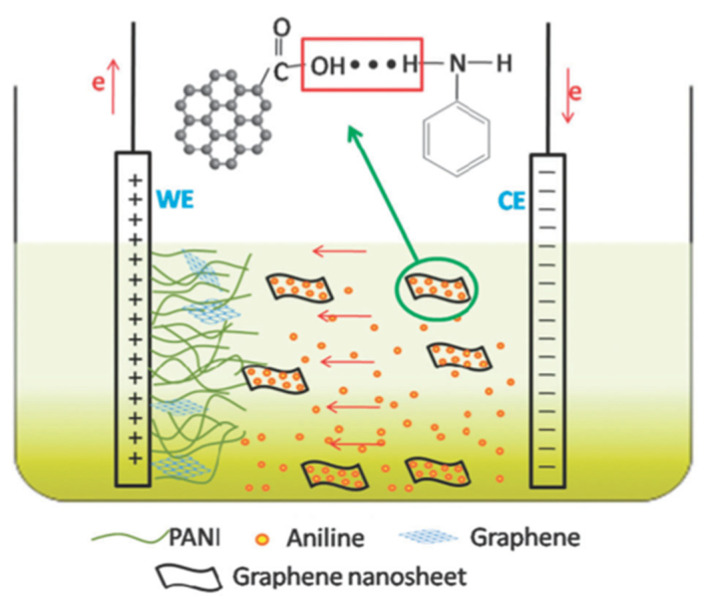
Scheme of in situ polymerization of nanorod-PANI-graphene developed by Hu et al. Reproduced with permission from reference [11]. Copyright (2012) The Royal Society of Chemistry.

Electrochemical polymerization is an essential in situ method in the preparation of CP films [157]. The control over the thickness and roughness, combined with the room temperature reaction conditions, rapid polymerization and catalyst-free nature of the reaction are some advantages of this technique. Due to their electrical properties, the introduction of graphene-based materials in the electrochemical polymerization is a good strategy for the formation of novel CP/G hybrid materials [11,158,159]. For instance, Hu et al. took advantage of the fast redox and acid-base doping/dedoping properties of PANI to produce nanorod-PANI-graphene films with controllable sizes (Figure 9) [11]. Graphene increased the nucleation rate of PANI, making it more accessible to the electrolyte. The charge–discharge rate of the new composite showed good capacitance and cycle stability due to the appropriate pore size distribution. The use of graphene derivatives, such as graphene oxide (GO), with many carboxyl, epoxide and hydroxyl groups on the surface is an excellent dopant method for electropolymerization, which is due to the fact that the GO surface is negatively charged, making it hydrophilic and well dispersed in aqueous solutions [160]. Wang et al. prepared—through the in situ electropolymerization of polyaniline and GO—a composite paper with flexibility and electrochemical activity. The presence of PANI showed an increase in tensile strength of up to 43% and an enhancement of the electrochemical capacitance up to 58%. The building blocks formed were suitable for use as flexible supercapacitors [161].

#### 4.1.2. Melt Processing

An alternative method, preferably used to scale up manufacturing processes, is the melting of the polymer without the presence of a solvent. Once the molten state is reached, graphene is incorporated using different methods, such as via a twin-screw extruder [162]. The graphene flakes can withstand high temperatures without their characteristics being altered and intercalate with the polymer chains during the process [163,164]. This technique is commonly used in industrial processes with thermoplastic polymers, since only simple parameters such as the screw speed, temperature and time need to be adjusted [163]. However, during the mixing of the dispersion, the quality and homogeneity of the resultant CP/graphene composite usually has lower standards than that obtained via in situ polymerization. Zhang and co-workers developed conductive polyethylene terephthalate/graphene nanocomposites, prepared via this technique [164] The incorporation of graphene improves the conductivity, with a low loading of the material (3% vol). The resulting composite showed a high aspect ratio and large specific surface area; in this case, the dispersion achieved during the fabrication was adequate to elicit a considerable improvement of the nanocomposite.

#### 4.1.3. Solvent Mixing

**Figure 10:**
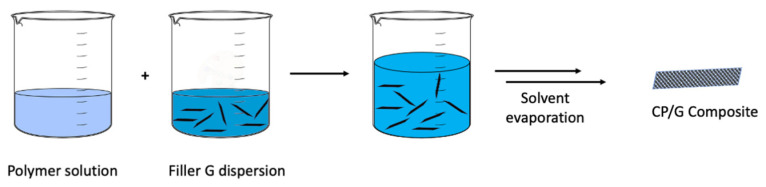
Scheme of the common solvent mixing procedure for the fabrication of graphene/CP composites.

The solvent mixing method is a common technique, where the matrix—after its polymerization—is mixed in solution with a dispersion of graphene-based material (Figure 10). This methodology is easier for scale-up processes and has fewer complications as it does not involve time-consuming chemical reactions. However, compatibility between the solvent chosen to dissolve the matrix and the graphene dispersion is imperative. In many cases, before solvent evaporation, the mixture needs to be exposed to a powerful homogenization technique, such as ultrasonication. For instance, Qiu and co-workers developed a concentrated graphene aqueous solution using ultrasonic vibration and PPy as intercalators [165]. The π−π interaction of PPy with graphene allows for exfoliation of the material to a thickness of less than five layers without increasing the graphene defects. The new composite has enhanced corrosion protection qualities in an environment-friendly aqueous media. The evaporation process—after mixing—can be varied; an example is described by Subtil et al. [166]. The authors developed a new polyethersulfone (PES) membrane by mixing PANI and rGO using a phase inversion process. The new membrane showed higher conductivity and changes in roughness; these modifications increased the permeability, porosity and hydrophilicity of the membranes, due to the fabrication method. 

#### 4.1.4. Electrospinning

The versatility of electrospinning (ES) comes from the facility to produce ultrathin fibers from many precursor materials [167]. The formation of fibers is favored by the application of electrostatic forces to a jetting polymer solution [168]. More specifically, the dynamics of electrospinning resemble those of electrospraying, in which an electrostatic charge is used to obtain an aerosol of liquid charged drops from a capillary tip. In a typical process, a solution of the polymer is loaded into a syringe and pushed to the tip where it forms a droplet. A jet of solution is formed when a high voltage is applied to the tip, modifying the shape of the drop and forming an initial structure called a Taylor cone and electrostatic repulsion building at the drop withdraws a jet of the material, which is deposited on a collector resulting in the formation of a continuous homogeneous fiber. Fibers with diameters of a few hundred nanometers can be obtained in a random orientation.

The introduction of carbon forms with polymers in ES offers structural advantages in the fabrication procedure and enhances the mechanical properties, resulting in a high surface area composite being obtained. The suspension of graphene can be mixed easily with the electrospinning solutions [169,170,171]. For example, Moayeri et al. showed how the introduction of rGO in a PANI solution improves the fibers’ properties, increasing the thermal degradation and electrical conductivity without alterations to the electrospinning process [12]. In the last decade, the number of applications of these mixing solutions/inks has increased, making them suitable for the fabrication of 3D scaffolds for tissue engineering, sensor substrates, and membranes for ultrafast photonics [172,173]. 

### 4.2. Characterization and Properties

To understand the properties and the formation of graphene embedded in CP, common characterization techniques can provide data such as the amount of graphene loaded on the sample, the confirmation of covalent bonding between the polymer and the graphene, whether the aromatic character of the mixture has been disturbed, or the precise chemical composition, as well as its optical, morphological and electrical properties. Morphological characterization of the composites gives us information about the roughness of the mixture, number of layers, thickness, length and particle size. For instance, for electrospinning fabrication, SEM is a reliable and fast tool to determine the morphology of the fibers [12]. Additionally, SEM–EDX can provide extra information about the composition and homogeneity of the sample [174]. 

The properties of how the graphene in the CP is embedded can be studied by several techniques. For instance, TGA allows one to study the loading of the sample by analyzing the weight loss proportion of the composites [152,175], while FT-IR and UV–Vis can identify specific groups of the CP backbone to confirm that no degradation has occurred during the fabrication process [153]. Moreover, the chemical composition of the sample, such as the oxygen functionality after reduction of the composite and its bonding structure, can be studied by XPS [153]. 

Raman spectroscopy is a powerful technique for this purpose as well. An intense band of graphene appears around 1580 cm^−1^ (G band) and is standard to all graphitic nanomaterials, while the so-called 2D band, which appears between 2500 to 2900 cm^−1^, is the second-order mode and is related to the number of graphene layers. Additionally, covalent grafting can be confirmed by the D band, placed around 1350 cm^−1^. Bose et al. determine the bonding structure from the intensities and shifts of the Raman spectra of their composites [156].

The electronic characterization of CP/G showed improved properties due to the introduction of graphene, which enhances the electrochemical performance. Several techniques can be performed to characterize such composites. For instance, impedance spectroscopy is a procedure that measures the impedance response of the composites. Chang et al. determined that an rGO/PPy composite had a lower charge transfer resistance and better capacitive behavior than the homologue without rGO [176]. These observations coincided with studies conducted through cyclic voltammetry, which measures the current galvanostatic discharge curves, to measure the charge–discharge time capacity.

### 4.3. Applications

The unique properties of CP/G composites are widely implemented in several applications; some of them are summarized in Table 1.

#### 4.3.1. Biomedical Applications

The introduction of graphene into biomedical fields has increased in recent years thanks to its electrical, physical, and chemical properties. Practically, the use of graphene derivatives, such as GO or rGO, has demonstrated great potential due to the great integration of the nanomaterial with biological systems [186]. 

The electrochemical polymerization of PEDOT in the presence of GO showed good biocompatibility and excellent conductivity, with easy integration of the GO into the matrix [160]. After 24 h, Luo and co-workers showed how the PEDOT/GO films were trivially cytotoxic, promoting neuron growth and great neurite length. The free functional groups available on the surface allowed for the immobilization of a functional laminin peptide, preserving its bioactivity. This composite also showed low impedance, easy biomolecule functionalization, and good potential for tissue engineering applications [160]. The use of PEDOT/GO enhances mechanical properties and can amplify the electrode’s active surface area, making them biocompatible for electrical stimulation [179]. When studying the use of conductive polymers and graphene as electrodes, Ouyang and co-workers prepared a PEDOT/G/GCE dispositive that produced excellent results for the simultaneous detection of four DNA bases without any treatment or preparation. The electrode exhibited excellent electrocatalytic activity, making it a promising tool in clinical genetic engineering diagnostics [180]. The electrical properties of PEDOT/graphene electrodes make them great candidates for many biosensing applications [127,181].

The controllable conductivity of PANI combined with graphene allows one to create flexible, electrochemically active, and biocompatible hybrid papers that are attractive for tissue engineering, drug delivery and biosensing applications [177]. Moreover, some theoretical studies have pointed out that graphene/PANI composites exhibit high sensitivity and adsorption capabilities in recognizing NH_3_ gas molecules, thus, making them suitable for ammonia sensors [187]. Muthusankar and Ragupathy developed a copolymer PANI-PDPA hybrid impregnated with graphene nanosheets via copolymerization of the prepared PANI/graphene composite and PDPA/graphene blend, observing a homogeneous dispersion of the sheets [188]. Additionally, the hybrid was prepared for the immobilization of glucose oxidase onto an ITO electrode and it showed an electrochemical determination of glucose over a broad linear concentration range with good sensitivity and fast response.

**Figure 11:**
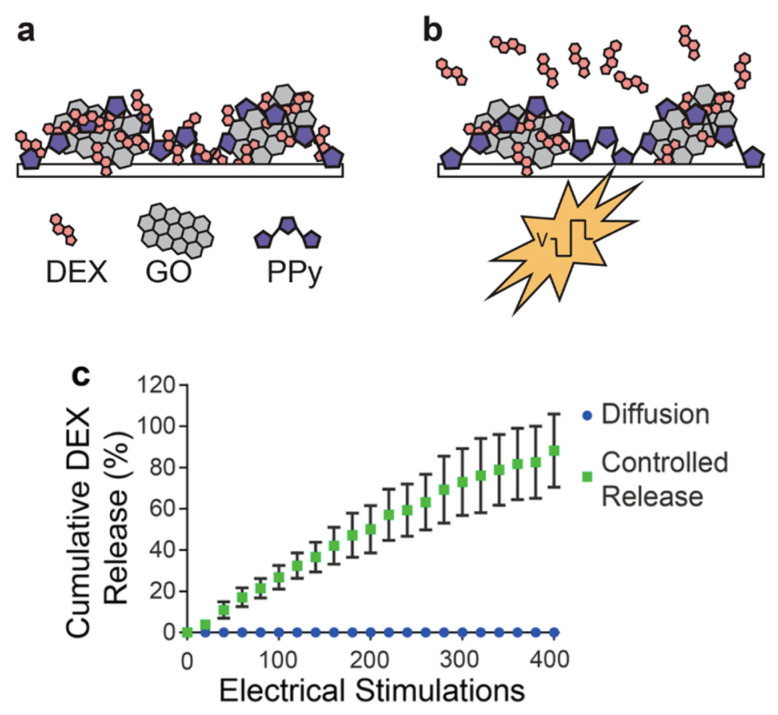
Illustration of GO/PPy-DEX nanocomposite (**a**) and its DEX release (**b**) after electrical stimulation. (**c**) Cumulative release of the nanocomposite for up 400 pulses, without electrical stimulation no drug release was observed. Adapted from reference [184].

An electrically controlled drug release procedure was also developed. Weaver and co-workers used GO nanosheets incorporated into a composite of PPy. The GO/PPy nanocomposite was loaded with dexamethasone (DEX), an anti-inflammatory drug; it showed a lineal release of the drug that hold hundreds of stimulations only when a voltage is applied [184]. Interestingly, no drug release was observed in the absence of stimulation. Additionally, the device did not present toxic by-products and could be regulated depending on the size and thickness of the GO nanosheets (Figure 11). This platform was an excellent candidate for on demand drug delivery. Finally, it is worth mentioning one last exciting application, that was developed by Byun et al., who developed a hybrid nanostructured bioenergy storage device of GO and PPy [185]. 

#### 4.3.2. Electronic Applications

The exploration of new materials to optimize the structural and electrical properties is ongoing. CP/graphene composites are good candidates for their fast doping/undoping process, as well as the electrical properties of graphene mentioned above. Energy storage is a crucial application nowadays due to the use of portable electronics, supercapacitors, and electric vehicles. The use of PPy is promising, mostly because of its high stability in air and easy production in aqueous solutions. However, the lack of stability during cycling suggests the possibility of introducing graphene into the nanocomposite to create a synergy between them [189]. Supercapacitors are a promising source of power generally used in applications that require fast charge/discharge cycles in electronics equipment. Chang and co-workers developed a new approach using a PPy/rGO film with supercapacitance properties [176]. The rGO expanded its surface area, and the assembly of the device was performed in a one-step electrochemical approach. Biswas et al. aligned flakes of graphene embedded in a PPy fibrous network [36], and the resulting electrode showed high ionic and electric transport. Impedance analysis exhibited low equivalent resistance between the graphene basal plane and the polymer and the composite revealed great electronic charge transport and cyclic stability.

PANI has also attracted attention as a framework in supercapacitors due to its thermal stability, tunable properties, high conductivity and low-cost fabrication processes. Additionally, the color change from various redox reactions makes this CP a promising candidate for electrochromic applications. For instance, Lyu fabricated PANI-based hybrid films by electropolymerizing the monomers onto ITO glass slides with WO_3_, graphene and a heterostructure of both [178]. Cyclic voltagrams and galvanostatic charge–discharge graphs showed better areal capacitances in the case of WO_3_/G/PANI. Faster switching responses and higher coloration efficiencies were observed in the multi-composite. The rougher morphology of the sample and the embedment of the nanofiller into the matrix played an important role in this effect. The use of photocapacitors in self-sufficient energy composites is an ambitious research topic that aims to simplify charge transport and storage processes. PANI, PEDOT, and PPy are suitable candidates due to their properties, as already mentioned in this review. Gao et al. integrated a carbon supported graphene/PEDOT electrode and an ionic liquid electrolyte [182]. The resultant device showed good stability and even presented performance under low intensity illumination.

#### 4.3.3. Sensing

Finally, CP/G has been used for chemical sensing, creating a large surface platform that amplifies the adsorption behavior, with the increased surface area producing increased analytical recognition spots. Additionally, CPs can act as sensitive agents and enhance the selectivity of some substances that graphene alone would not adsorb. The combination of these composites decreases the broad detection limit and response time and enhances the sensitivity and selectivity [190]. Yang et al. prepared an in situ porous film of PEDOT/rGO with an efficient chemiresistor platform for NO_2_ detection. The large surface area of the composite enhances gas desorption and adsorption. The authors confirmed the sensitivity by exposing it to different concentrations of NO_2_ gas, obtaining repeatable characteristics. Additionally, during the fabrication process, the porosity of the material can be controlled by modifying the heating rates in the baking process [150]. 

**Figure 12:**
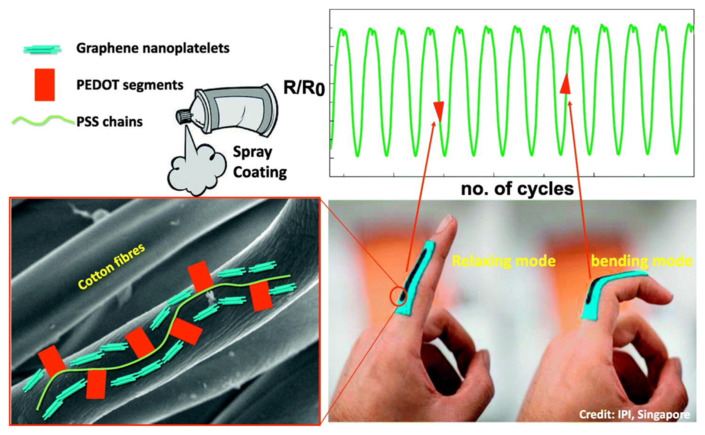
Representation of the cotton fabrics of PEDOT:PSS/G with strain response. Reprinted with permission from reference [183], Copyright Elsevier (2017).

As one last example, the conductivity of PEDOT allowed Zahid and co-workers to mercerize woven cotton fabrics using PEDOT:PSS and graphene nanoflakes up to 20 wt% by spray coating [183]. The new conductive fiber was lightweight, completely flexible, and resistant to laundry cycles, while it also showed resistance under heavy weight. Additionally, the fabric showed a strain deformation response with a 90% viscoelastic recovery after 1000 cycles. These conductive fibers are great candidates for wearable bionics and sensing (Figure 12).

## 5. CNT Embedded with Conjugated Polymers

Carbon nanotubes (CNTs) represent a the state-of-the-art nanomaterial for application in any field involving nanotechnology, ranging from electronics to tissue engineering [191]. Their structure can be described as a rolled-up sheet of graphene; the way in which the graphene sheet is rolled on itself defines the diameter and the helicity of the obtained CNT. These features are known to directly influence the characteristics of the CNT, especially its electrical behavior [192,193]. The highly unusual electronic properties of CNTs arise from their nanometer dimensions together with the peculiar electronic structure of graphene-like sheets; their behavior as metals or semiconductors is highlighted on the basis of the diameter and helicity of the tube. Furthermore, the excellent mechanical properties of nanotubes, and carbon nanomaterials in general, arise from the strong carbon–carbon chemical bond in the graphene-like lattice. For this reason, CNTs are used in many applications as fillers in order to reinforce the structure of composite materials. 

CNTs remarkable characteristics have helped their rise as one of the most commonly used building-blocks for the production of advanced materials. Composites that incorporate CNTs have shown superior mechanical and electronic properties. Concerning the mechanical properties, the CNT-containing substrates have shown to demonstrate increased Young’s modulus values and enhanced tensile strengths with increased CNT loading [194]. It is worthy to note that the homogeneous distribution of the filler in the matrix should be taken into account when comparing the composite to the neat polymer. Functionalization of the CNT surface also influences the scaffold’s mechanical properties. The different kind of derivatization groups used to modify the surface of pristine CNTs can be specifically chosen to improve the mechanical properties of hybrids by interacting with the structure of the polymeric network. Hybrid scaffolds also take advantage of the electronic properties of CNTs. In all the studies reported so far, the conductivity is directly proportional to the number of nanotubes present. 

Such hybrid materials have proved to be valuable tools in biological applications, such as in tissue engineering, sensing, and drug delivery, as well as in the preparation of batteries, energy storage and the assembly of microelectronic devices [195,196]. In particular, the inclusion of CNTs with conjugated polymers has been widely studied, and the literature includes plenty of examples where CNT/CP have been combined in multiple ways and with various architectures for use in electronic and biomedical applications. In this section we aim to give an overview on the most recent advances in respect to embedding CNTs with conjugated polymers. 

### 5.1. Fabrication Approaches for Obtaining CNT/CP Materials

An extensive number of different polymers have been used as matrices for the production of hybrid materials, with CNTs giving rise to enormous libraries of composites. The final application of the structure generally dictates the synthetic method used, with the two main routes being in situ and ex situ methodologies. Despite of which synthetic pathway is used, efforts must be made in order to obtain a dispersion of CNTs. Nanotube aggregates may weaken the final structure by impeding the formation of a homogeneous polymer network, as well as limiting the conductivity and overall biological applicability of the composite [197]. The strong π–π stacking forces between CNTs, given by their large aspect ratio, hinder the individualization of the single tubes in solution. Generally, a good dispersion of nanotubes is achieved by ultrasonication or ball milling of the carbon nanomaterial. More recently, dispersion has been improved, implying the possible use of surfactants or surface functionalization of the nanotubes [198]. For the up-scaling and large-scale production of composites, ex situ methods are preferred over in situ ones. 

In situ methods indicate the synthesis of a composite when the polymerization reaction of the matrix is performed as a second step after the mixing of the monomers with the CNTs. The mild conditions used in this method make it useful for the preparation of insoluble polymers or those that cannot withstand high temperatures. In situ methods are preferred when working with conductive polymers, such as PPy, PEDOT or PANI [199,200,201]. For instance, Dominguez-Alfaro et al. manufactured 3D conductive PEDOT/CNT and PPy/CNT scaffolds using the well-known vapor phase polymerization methodology, where EDOT was in situ chemically polymerized inside a CNT-containing template [199,200,202]. Furthermore, the same authors succeeded in the fabrication of 3D PEDOT/CNT scaffolds through electropolymerization of EDOT and electrodeposition within a CNT–sucrose template [203]. It is worthy to note that these are the first and unique examples in the literature where a 3D structure is uniquely composed of CNM and CP. As well, in these examples, the amount of CNTs embedded in the final composite was precisely controlled, which is usually difficult to achieve, making this the main drawback of the synthesis pathway.

On the other hand, methods in which the mixing of the filler occurs after the polymerization of the matrix, are referred to as ex situ methods. Mixing can be achieved by solubilizing a polymer and dispersing CNTs in common solvents or incorporating the nanotubes within a melted thermoplastic polymer framework. In the first case, i.e., solution mixing, a suitable solvent for both the nanotubes and the polymer is essential. Homogeneity of the CNTs dispersion within the matrix is a key point to obtain good composites, and simple stirring is usually ineffective. Therefore, ultrasonication has been extensively used for solution mixing, and surfactant or polymer wrapping techniques have been implied to individualize CNTs in solution and avoid the formation of defects on the CNT surface. In this line, PEDOT:PSS has been shown to wrap around the CNT cylinders, favoring its dispersibility [204]. Afterwards, the final composite is afforded throughout precipitation or evaporation of the solvent. On the other side, functionalization of CNTs has also even been used to improve their suspension in water. Thus, COOH-CNTs have been highly dispersed in water and immediately after, electrochemical deposition of PEDOT:PSS:CNT-COOH on glassy carbon electrode was achieved [205].

Overall, multiple original and modified strategies have been reported for fabricating structures containing CNTs and CPs, including 2D coating films, micrometer and nanometer size fibers, 3D composites and hydrogels. 

#### 5.1.1. Coated Films

Coated films, also known as 2D films, thin films, or simply coatings, are widely employed in electronic and biomedical applications to obtain nano- and micrometer sized film thicknesses of conductive materials and provide conductivity to an insulating structure of either 2D or 3D nature. The most typical and common approaches are simple, easy and quick: spraying, spin-coating, drop-casting and filtration after mixing. In addition, other complex techniques have also been employed for producing a thin film, such as chemical vapor deposition for CNT coatings, and vapor phase polymerization or electrodeposition for CPs [206]. All of these methodologies allow for the wide homogeneous covering and permeability of any kind of surface, even those made from rugose or porous materials.

In the literature, single layers are formed firstly by mixing and dispersing the CNTs with the conjugated polymer solutions [207,208,209,210]. For thin film bilayer fabrication, initially, a dispersion of one of the materials, i.e., CNT or the conjugated polymer, is prepared and coated on the target substrates, such as flexible polyethylene terephthalate (PET) [211]. Then, the first layer is covered with the remaining material. Coating CNT first and then, afterwards, the conjugated polymer, embeds and densifies the nanomaterial, increases the conductivity, wettability and decreases the roughness of the resulting films [212,213]. Electropolymerization deposition has also usually been employed for this purpose. In this case, the coating deposition is performed in a standard three-electrode cell configuration, with the CNT-coated film as the working electrode and monomer electrodeposition—such as EDOT or pyrrole—occurring during its own electrochemical polymerization [39,214]. Overall, CP/CNT films showed higher stability when compared to CNT-free films, and no typical peel off of, for instance, PEDOT:PSS during the post-processing with nitric acid treatment was observed [215,216].

#### 5.1.2. Fibers

Natural or synthesized fibers are non-conductive; thus, CNTs and CP are used to introduce electrical properties within the fibrous matrix [217]. Moreover, CNTs offer the advantage of enhancing the mechanical properties of the fiber making this structure extremely useful in multiple applications, such as drug delivery systems, sensors or electronic devices [218,219]. 

The production of embedded CNTs/polymer fibers is typically achieved via electrospinning [220], due to the simplicity of the preparation method and the wide variety of composition and dimensions that can be obtained. Different kind of polymers have been used in the literature, with both SWCNTs and MWCNTs. Natural polymers, such as silk fibers or agarose, gave rise to composites with increased mechanical properties [221,222]. Synthetic polymers are often used as well, such as—for example—polyurethane or polylactic acid with fiber diameters ranging from hundreds of nm to µm [223,224]. Furthermore, not only does the CNT and CP affect the electrical properties of the fibers, but their mechanical properties, structure, and dimensions also have an effect. Polyacrilonitrile (PAN)/PPy/CNT nanofibrous scaffolds were prepared by electrospinning a blend containing the three elements in multiple concentrations and the resulting effect on the nanofibrous properties was analyzed [225]. It was observed that the average diameter of electrospun nanofibers decreased with increasing PPy concentrations, while agglomerated CNTs caused beads and disordered parts on the surface of the nanofibers. Interestingly, the electrospinning was limited to concentrations of 10% PPy, since solutions with higher amounts of PPy that also incorporated CNT could not be electrospun.

The conductive properties induced via incorporation of CNT and/or CP, have led to the use of these materials as coatings for already prepared fibrous mats. In connection with the previous section, multiple simple coating methods have been used. For instance, polyacrylonitrile/CNT nanofibers were produced by electrospinning, followed PPy coating by in situ chemical polymerization [226,227]. In another example, spray coating was used to deposit CNTs on a paper-like substrate consisting of electrospun poly(vinylidene fluoride-co-trifluoro ethylene) (PVDF-TrFE), with subsequent coating with PEDOT via vapor phase polymerization (VPP), leading to the production of transparent electrodes in a piezoelectric pressure sensor [228]. Even though the conjugated material is commonly the last one to be added, a few studies have added carbon nanomaterial in the last step. Hazarika and co-workers first deposited PPy on Kevlar fibers (WKF) by in situ chemical polymerization, before growing Fe nanoparticle-decorated CNTs on top under microwave irradiation [229]. However, this was not the outer layer of their system, as they finally covered it with a Ag-graphene dispersed in polyester resin (PES) by vacuum-assisted resin transfer molding. Such combination resulted in composites with remarkable enhancement of the tensile properties and impact resistance.

The desired size of the fibers depends, of course, on the application it is designed for. In some case, such as intramuscular electrodes for stimulation, thicker fibers or wires are required. Such architectures cannot be prepared by electrospinning techniques, but via extrusion of a composite mixture. In this way, soft wires composed of silicone, PEDOT-PEG and CNT have been fabricated [230]. Interestingly, the combination of PEDOT-PEG with CNT resulting in simultaneously boosting the conductivity of the electrode and minimizing the CNT content (below 8%) to avoid potential CNT leaching and maintain the elongation and tensile strength of the silicon composite. Moreover, increasing the amount of CNTs resulted in larger wire diameters and higher variability in the Young’s modulus.

#### 5.1.3. Tridimensional Architectures

Until recent years, carbon nanomaterial science was devoted to the construction of bidimensional materials, given the nanoscale size of the structures used and the ease of the methodologies used. However, controlling and building in a three-dimensional manner is essential in the current applications, not only because the world is three-dimensional, but also to enhance the effectivity of the materials by increasing the functional surface area. 

For instance, to favor its acceptance in tissue engineering, the implants are required to mimic the biological environment’s porosity, permeability and mechanical stability to avoid possible rejection from the host tissue [231]. Current in vitro studies are based on bidimensional cell cultures to analyze the material’s biocompatibility, being far from the complexity and heterogeneity of real systems. One alternative is animal models, although they are expensive and time-consuming, and the ethical issues make them unfeasible for most projects, keeping them for final studies. Thus, tridimensional scaffolds appear as an effective strategy for tissue regeneration, not only because the results can be obtained faster, but also because they allow for the creation of cultures similar to in vivo tissues, having large surface areas for cell or biomaterial attachment, proliferation, sensing, etc. [232]. Several studies have demonstrated that cells have behaviors and responses when cultured in a 3D organization that is more similar to those that occur in vivo, providing a more realistic predictive outcome [233].

The main drawbacks of the few 3D architectures composed solely of CNTs that have been reported, include their poor structural properties and toxicity. The use of a polymer showed reductions in the cytotoxicity of CNTs in cell cultures. Additionally, the presence of a matrix fixes the CNTs spatially, preventing them from moving freely and circulating through the body. Using CNTs as fillers in a polymer matrix also positively affects the mechanical and electrical properties of the structures. In this way, 3D hybrid materials can be produced with more interesting and useful characteristics compared to the polymers or the carbon nanomaterial alone [234]. Controlling the different amount of the constituents of a blend can lead to the fine tuning of its properties and often a synergistic effect is observed in between the nature of matrix and the filler. 

CNTs or CP are not able to produce a 3D structure by themselves. In general, a large variety of polymers, dopants, compositions and biomaterials have been developed as 3D scaffolds to achieve the best material for a certain application, including fibrous mats, papers, foams and hydrogels. Most of them are composed of a non-conductive polymer matrix filled with CNTs and CPs to provide conductivity [235]; however, several papers have appeared showing original and peculiar strategies to achieve 3D architectures, each one of which deserves a proper description. In this section we will review the design and manufacturing processes of some of these latest distinctive CNT/CP-embedded hybrid materials.

**Figure 13:**
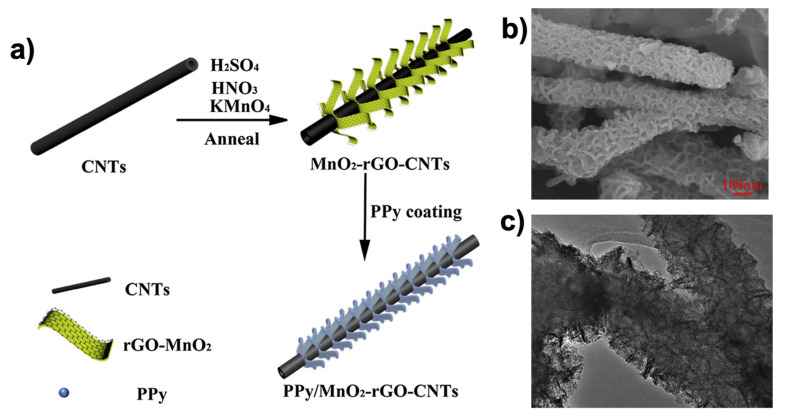
(**a**) Schematic illustration for the synthesis of the core-shell 3D structured PPy/MnO2-rGO-CNTs composite. (**b**) SEM and (**c**) TEM image of the PPy/MnO2-rGO-CNTs composite. CNT: carbon nanotubes. Adapted with permission from reference [237], Copyright (2017) Elsevier.

**3D fibrous mats and papers.** Molding the fiber leads to the production of 3D architectures with a porous structure. Thus, the challenge is to produce 3D structures with this approach, and there are scarce examples in the literature that succeeded in the manufacture of 3D structures using new and original strategies [236]. From our point of view, fibers from a few micrometers to a mm size can be considered 3D structures. These kinds of web-like networks have attracted attention as excellent substrates for electron and charge transfer applications, and the originality of their design and fabrication is worth mentioning. For instance, CNTs have undergone strong acid treatment and annealing to yield a kind of a banana-peeled rGO/CNT structure with rGO nanosheets attached to the CNT backbone, clearly shown in the SEM and TEM images shown in Figure 13 [237]. The flexible rGO has the double function of being a reactive material and a carrier for subsequent MnO2 in situ growth. Afterwards, PPy nanoparticles were wrapped on the MnO2 nanosheets by in situ polymerization, enabling the interfacial stabilization and providing a buffer layer to accommodate the volume expansion. The introduction of CNTs in the MnO_2_ structure favored the energy storage of the system, facilitating fast electron conduction and maintaining structural integrity [237]. In a second example, Zhang et al. used a carbon cloth as a substrate for anchoring randomly arranged CNT arrays via chemical vapor deposition (CVD), thus, forming a strong and robust integrated 3D CNTs network. Afterwards, the network was decorated with a first layer of MnO_2_ and a second of PEDOT by electrochemical deposition, resulting in an innovative flexible 3D CNT conductive framework with good mechanical and storage properties that addresses the sluggish storage issues of the MnO_2_ cathode for next-generation quick charging electronics [238]. 

**Figure 14:**
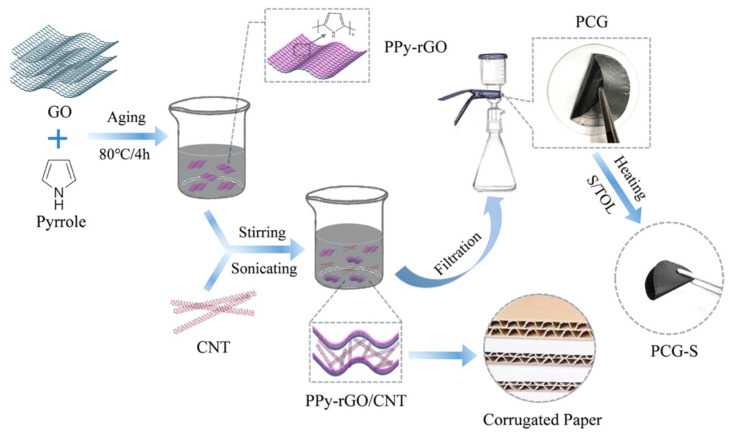
Schematic diagram of the synthesis process and structure of PCG-S paper. Reprinted with permission from reference [38]. Copyright (2020) American Chemical Society.

Paper-like composites have been attracting a great deal of attention as emerging lightweight flexible electrode devices. As the name implies, they are a thin sheet of material composed of fibrous-like mats with thicknesses in the range of microns to mm and shapes similar to the common cellulose paper. In fact, cellulose has been used as the substrate to fabricate PPy/CNT/cellulose composite films: first, a simple freeze-and-thaw approach was used to incorporate functionalized CNTs into the cellulose, resulting in a homogeneous porous composite with strong hydrogen bonding between the two species; later, PPy was uniformly coated via in situ chemical polymerization [239]. As a result, the modified paper showed high electrical conductivity, good wettability, a porous architecture, and large interfacial areas for the storage/release of charge carriers and for the facile diffusion of electrolyte. On the other side, papers can also be generated through a bottom-up assembling approach by filtration. In this line, Liu and co-workers fabricated a conductive paper with CNTs and SnS@PPy-nanobelts as building blocks by in situ polymerization of PPy on SnS nanobelt substrate and subsequent mixing with CNTs and filtration [240]. Similarly, as represented in Figure 14, a self-standing and flexible PPy@rGO/CNTs (PCG) paper was constructed by filtration after integrating CNTs into PPy@reduced graphene oxide hybrid structure, rooted from in situ redox reaction and spontaneous assembly of pyrrole and graphene oxide [38].

**Foams.** Due to their low processability, preparing 3D CP-based materials is tricky; however, the number of different strategies to manufacture these devices has increased significantly in recent years. Such processing methods are based on the use of a nonconducting template to define the 3D geometrical shape, as well as the internal configuration, and post-coating with CP to provide conductivity. 

The inclusion of CNT into the template is commonly prepared before the polymerization of the CP. Usually, a melamine sponge is repeatedly immersed into a CNT dispersion, compressed, and then loosened to promote complete adsorption of the nanomaterial [241,242]. Although this is the simplest step, it should be noted again, that a good dispersion is important to avoid aggregation, improve homogeneity and, thus, obtain high quality scaffolds. In the second step, in situ polymerization of the CP is performed. Such a coating can be performed via immersion, chemical polymerization, electropolymerization or a combination of these. As with the first step to incorporate CNT, the immersion is produced repeatedly, infiltrating an already prepared PEDOT-based solution within a CNT-containing carbon foam following compression and loosening [241]. Different amounts of loading could be easily obtained with a number of immersions. In another example, PPy-carboxymethyl cellulose films were in situ polymerized via chemical oxidation in solution and in presence of a prepared CNT-sponge [242]. However, the methodology can be performed in a reverse order; for instance, Wu and co-workers first immersed a melamine sponge in a pyrrole aqueous solution, performed chemical polymerization, and finally, immersed the resulting PPy-sponge in a CNT/polydimethylsiloxane (PDMS) solution, before curing [243]. The PDMS helped in retaining the nanomaterial and avoiding loss or delamination.

Apart from chemical polymerization, electrochemical deposition has also been employed for coating 3D substrates. In this case, the CNT-based composite is used as the working electrode during the electrodeposition. Thus, a bulk sponge made of CNTs or CNT/graphene, synthesized by chemical vapor deposition (CVD), was directly employed to electrodeposit PANI or PPy using a three-electrode cell [244,245]. As well, a graphene foam has been used as the substrate to first grow CNTs through plasma-enhanced chemical vapor deposition and then used to electrochemically deposit PEDOT [246].

Usually, the template or initial matrix is essential for maintaining the 3D architecture and the porosity and, therefore, it is maintained after the coating. However, only one group published a study in the literature that demonstrated the ability to construct tridimensional scaffolds, with homogeneous porosity and low density, composed uniquely of CNT and a CP, in particular, PEDOT or PPy [199,200,203]. To achieve this, the CP was chemically (via vapour phase polymerization, VPP) or electrochemically polymerized on 3D molds prepared with sucrose and CNT. Synthesis through VPP, in contrast to conventional chemical polymerizations, occurs through a monomer in its vapor state, which allows for control of the deposition of highly conductive films. In this process, the 3D matrix is soaked into an oxidant solution; then placed in a closed chamber with the monomer solution. It must be noted that both components inside the chamber must not be in direct contact. Finally, the coated substrate is rinsed in an alcohol solvent and water to remove any unreacted species. The monomer vapor can be formed through heat, a high vacuum under an inert atmosphere, or both [247,248]. In some cases, the vapor has been stored or produced in a separated connected chamber, and guided through pressure differences to the reacting chamber [249]. The monomer vapor polymerizes chemically when it reacts with the oxidant inside the 3D substrate. After polymerization, the resulting scaffolds were washed to remove the sugar and yielding to self-standing 3D foams. 

The last processing strategy to produce porous scaffolds does not include a polymerization step, thus, it is the simplest method reported. In this approach, the CPs used are commercially available, such as the commonly used PEDOT:PSS Clevios PH-1000, from Heraeus. Although such a strategy is the fastest and easiest way to generate conductive 3D scaffolds ever published, delamination does occur more often than in the other processes mentioned above due to the weak interaction between the CP and the substrate. For this reason, instead of only dipping and letting dry, Chang et al. homogeneously dispersed the commercial PEDOT:PSS solution with CNT by ultrasonication, placed it in plastic dishes and then freeze-dried it [250]. Finally, the resulting sponge was infiltrated with liquid PDMS and cured, to maintain the stability of the final porous scaffold. 

**Hydrogels.** One of the most common forms of 3D structures are the hydrogels. These are soft self-standing materials made of polymeric networks, presenting a degree of crosslinking in their internal structures. Their name arises from the capacity to trap large quantities of solvent or water inside their matrix. Changing the nature of the polymer and its internal network structure gives rise to a tunability of the hydrogel features, including the possibility to modify its porosity, biocompatibility, and stiffness. Two approaches can be considered to obtain hydrogels: the macromolecular approach and the supramolecular approach [251]. In the first, the chemical gel is based on crosslinking, while in the second, the physical hydrogel is stabilized thanks to noncovalent interactions. However, basic hydrogels present many limitations related to their physicochemical properties, low mechanical strength and strain, low thermal stability and their inherent electrical insulation. For this reason, CNTs and CP have been increasingly used as fillers in hydrogel hybrid composites. Given their excellent mechanical strength, conductivity and surface area, CNTs and CP can reinforce the structure of the hydrogels and extend their properties making them promising nanofillers for biotechnology applications [252,253]. 

CNTs can be embedded in the internal network of hydrogels both chemically (covalently or supramolecular) and physically (mechanically) [254,255]. Rehman et al. showed how acrylamide hydrogels reinforced with oxidized MWCNTs are up to four times stronger than the pure hydrogel [256,257]. Via swelling tests at different pH levels, they assessed the degree of internal crosslinking and concluded that the hydrogen bond formed between the functionalization of CNTs and the polymer chains is the main factor responsible for the improved mechanical properties and self-healing behavior. On the other side, the conjugated polymer is usually introduced by in situ chemical or electrochemical polymerization before, after or during the cross-linking process of the hydrogel matrix. Castagnola and co-workers followed the former approach, by first synthesizing a PEDOT:PSS/COOH-CNTs coating through electrochemical deposition of an aquous solution containing the three species [258]. Afterwards, they polymerized poly(2-hydroxyethyl methacrylate) (pHEMA) hydrogel directly on the coating surface by single dipping into a prepolymer aqueous solution and subsequent UV light exposure. 

On the other side, a highly stretchable and real-time omni-healable CNT/poly(acrylamide)/PPy (CNT/PAA@PPy) hydrogel electrode was fabricated by changing the order of polymerization steps: initially, CNTs were dispersed within a prepolymer solution of acrylamide; then, the PAA was crosslinked; and finally, the CNT/PAA hydrogel was soaked in a pyrrole monomer solution, before subsequently being subjected to in situ polymerization with PPy, initiated by ammonium persulfate [259]. In another example, CNT was used as both the physical cross-linker of N-isopropylacrylamide (NIPAM) and, together with PPy, the conductive component. They were obtained, as was the case before, by first producing the NIPAM/CNT hydrogels and then embedding PPy via immersion in a pyrrole solution, with subsequent chemical polymerization. These hydrogels, also containing β-cyclodextrin (β-CD), exhibit high conductivity, self-healing properties, flexible and elastic mechanical properties and rapid stimuli-responsive properties both to temperature and near-infrared (NIR)-light [260]. 

In a third approach, oxidative cross-linking was employed to simultaneously crosslink catechol-functionalized hyaluronic acid (HA−CA) hydrogels and in situ polymerize PPy in a dispersion that also contained CNT [261]. These kind of hydrogels are composed of natural polymers and are mostly used for biotechnological applications, since they ensure excellent viability in cell cultures of the 3D matrix [262,263,264]. In particular, the abovementioned example, i.e., electrically conductive HA hydrogels incorporated with CNTs and/or PPy, were developed to promote differentiation of human neural stem/progenitor cells (hNSPCs) [261].

### 5.2. Applications of CNT/CP-Based Materials

Since their discovery, CNTs have come to represent the leading frontier in nanotechnology due to their unique electrical, mechanical and thermal properties [265]. Their high mechanical strength and low weight, combined with their conductivity and stability makes them useful materials for field emission, energy storage and molecular electronic applications, but they also show great potential in biomedical applications [266,267]. The wide range of possibilities for using hybrid materials embedded with CNT and conjugated polymers mainly stems from the enhanced properties these conductive materials provide to the desired substrates. Conductivity is, of course, the most important asset for electronic applications, although it is gaining importance in biological applications, such as tissue engineering, since the polymer species used to build biomaterials so far are electrically inactive. Mechanical strength, tensile strength and degradation are among the physical properties that are also boosted by the effect of CNT and/or CP, as we have observed in multiple examples throughout this review. In fact, the presence of CNT has solved an urgent problem in the application of PEDOT:PSS in electric devices: CNT lessens the “peel-off” effect of PEDOT:PSS coated layers, thus reducing degradation of the film due to water absorption and aging, while maintaining high conductance, high transmittance and low resistance [208]. Furthermore, CNTs have been also tagged as promising organic thermoelectric materials for due to their large thermopower [268]. The addition of CNTs to PEDOT:PSS films has led to a noticeable increase not only in the electrical conductivity of the films, but also in their Seebeck coefficient values, leading to an enhancement of thermoelectric properties [209,210]. Lastly, incorporation of CNT is equivalent to incorporation of rugosity and, therefore, the inner surface contact area within the hybrid structure, which can be either 1D, 2D or 3D. The nanoneedle-like particles favor the direct contact of individual species, such as nanoparticles or cells, with the conductive components and build an interconnecting CNT framework that facilitates both mass and charge transfer, thus, intensifying the characteristics of the resulting device. For instance, incorporating CNT is positive for electrode materials that usually require carbon incorporation to improve capacity and stabilize cycling, such as titania-battery applications. Yang et al. demonstrated how introducing a double-fold CNT network on titania-nanoparticles anodes coated with PPy ensured direct contact of individual titania nanoparticles with the conductive elements, and guaranteed both efficient electrical connection of active materials and resilience against cycling [269].

Overall, CNTs have shown to always have a beneficial effect when introduced into hybrid composites. Herein, we will review such effects in some of the most common applications in more detail.

#### 5.2.1. Biomedical Purposes

Multiple experimental studies suggest that the use of nanoparticles in medicine may revolutionize therapeutic success in numerous diseases, e.g., neoplastic, heart, neurodegenerative diseases and wounds that are difficult to heal [270,271]. More specifically, CNTs are one of the most promising materials to interface with the central nervous system (CNS). They are currently being used as growth substrates, scaffolds for nerve tissue engineering, electrode coating, long-term implants, and as drug delivery agents and molecular sensors [272,273]. Over previous years, it has been reported that CNTs promote the growth of neurons and the extension of their axonal processes in all directions [274], modulating neuronal behavior at either the structural or functional level [275,276,277,278], boosting the frequency of spontaneous events [279], and even inducing neuronal differentiation [280,281]. The recipe for the successful influence of CNT on neuronal networks comes from the physical interface along the cylindrical morphology of the nanomaterial and the electrical contact between the conductive nanomaterial and the electroactive cells [282,283]. Neuronal membranes establish a tight contact with the CNT substrate, and it has been hypothesized that CNTs can provide a kind of shortcut between the proximal and distal compartments of the neuron, promoting genuine biological processes, such as axon excitability and synaptic activity of neuronal networks [284,285]. Furthermore, the impact of CNT substrates on central nervous system (CNS) tissue has also been demonstrated [286,287]. Spinal cord (SC) and dorsal root ganglia (DRG) multilayered explants co-cultured on CNTs substrates showed a higher number of longer neuronal processes, and apparently integrating CNT as their own supports or exoskeletons [287]. Furthermore, cell culture platforms combining CNTs, PEDOT and iridium oxide showed excellent and larger resistance to inflammatory insults of primary cultures of neurons, of astrocytes and co-cultures of both [288]. 

**Tissue Engineering.** Tissue engineering is focused on the repair or replacement of damaged or diseased tissues with synthetic and/or natural implants. The conductive scaffolds aimed at regeneration must be biocompatible with the host tissue without inducing inflammatory or immune reactions, allow cellular adhesion and extension, offer proper physical support, and mimic the biological environment, i.e., provide physical properties similar to the native tissue [289,290]. Again, CNTs have been suggested as excellent materials for this purpose. 

**Figure 15:**
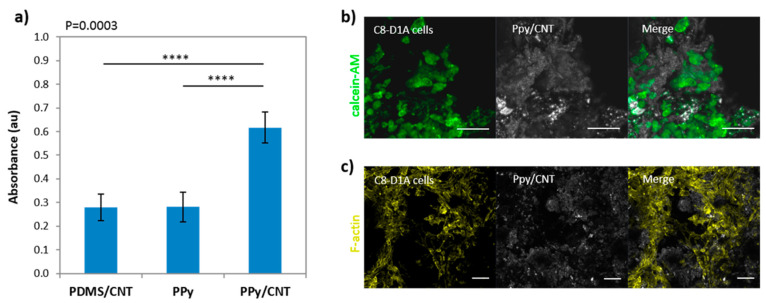
(**a**) In vitro cytotoxicity assay of C8-D1A astrocytes cultivated for 48 h on scaffolds in the presence or absence of CNTs. Absorbance readings for each scaffold are plotted as an average of five independent experiments (*n* = 4 ± SD). Confocal images after (**b**) calcein-AM stain of viable cells (green) and (**c**) the F-actin cytoskeleton (yellow) staining of PPy/CNT scaffolds after 2 days of culture. From left to right: stained cells (green or yellow), scaffold (gray), and merge. Images are split views of Z-stacks maximum intensity projections (57 μm optical sections). The elongated morphology of the cells indicates a good biocompatibility of the material. Scale bar = 50 μm. Reprinted with permission from reference [199]. Copyright (2018) American Chemical Society.

The nature and composition of the material used is essential for successful regeneration: Roberts and co-workers demonstrated how primary embryonic rat motor neurons grown on thin films of alternating stripes of HA/CNTs and SiO_2_ selectively migrate towards, and adhere to, the CNT-containing substrate [291]. Conductive polymers have been proven to down-regulate glial reactions without affecting neuronal viability and function [292]. Highly conductive scaffolds, composed of PPy or PEDOT in combination with CNT, have shown higher ability to support neuronal growth and regeneration than non-conductive matrices, as shown in Figure 15 [199,200]. It is worthy to note that among the different CPs, PANI has shown certain toxicity and very low biodegradability, which limits its implementation in biomedical fields [194]. Moreover, the architecture of the scaffold structure is crucial for a successful impact on regeneration and multiple constructions, e.g., fibers, porous substrates or hydrogels, have been used as support materials for the CNT, allowing for the shape to be modified, along with the dimensions and stiffness of the final scaffold [293,294,295,296]. Fibrous structures are widely used as scaffolds, since their shape and distribution can mimic the extracellular matrix (ECM) more effectively than other architectures [297,298]. Finally, the micro- and nanotopography of the material at the cellular level are also fundamental for successful nerve regeneration and several studies have shown that cells behave differently on aligned and randomly oriented fibers [299,300]. For instance, Yardimci et al. demonstrated that human keratinocytes cultured on PANI/PPy/CNT nanofibers showed good adhesion and proliferation but, more importantly, the cells stayed among the nanofibers, influencing in their shape and size [225]. 

Additionally, conjugated scaffolds are used for electrical stimulation of electrical sensitive cells and tissues, thus providing a new kind of interaction with biological systems and favoring and acceleration of regeneration and healing processes of electroactive tissues, enhancing cell–cell and cell–substrate interactions [301,302]. For this reason, the combination of CP scaffolds and ES can be a safer tool for use in clinics. This is due to the fact that since the current applied goes through the scaffold structure, the electrolysis of the biological medium and, thus, the production of cytotoxic agents, are minimized [303]. 

**Stem cell differentiation.** One of the strategies used in tissue engineering to develop artificial tissues in the laboratory is stimulating the direct differentiation of pluripotent or mesenchymal stem cells. In order to guide the differentiation into the desired type of cells, growth factors and specific proteins are usually introduced in the culture media. However, the mechanical properties of the materials also play an important role in such differentiation [304]. Electrically conductive hyaluronic acid (HA) hydrogels incorporated with CNTs and/or PPy showed significantly promoted neuronal differentiation of human fetal neural stem cells (hfNSCs) and human neural stem/progenitor cells (hNSPCs), with improved electrophysiological functionality [261]. More specifically, calcium channel expression was upregulated, depolarization was activated, and intracellular calcium influx was increased. These data suggest a potential mechanism for stem cell neurogenesis and provide a promising cell-culture platform and tissue-engineering scaffolds to improve neuronal regeneration. 

**Monitoring electrodes.** Traditional microelectrodes, such as metal-based devices, present very high impedance and deliver low currents. CNTs have appeared as exceptional coating materials for metal electrodes, not only due to their excellent interface with neurons, but also due to their large surface area that decreases the impedance and increases the charge transfer capability [305,306]. Such enhancement significantly reduces the size of the implantable devices, preserving excellent electrical performances, thus improving recording and stimulation for long periods [307,308]. Furthermore, given the excellent interface of CNT with neurons also facilitates the anchoring of the cells directly and only onto the electrode sites, avoiding the use of external chemical treatments [309]. This beneficial effect has been observed in various kinds of neuronal recording and stimulation methods in vitro and in vivo [288,310].

PEDOT coatings are useful to guarantee low impedance and ideal electrode conductivity of miniaturized electrodes. However, coating them with CNT has improved their mechanical and electrical properties and, therefore, their recording quality and stability [205]. On the other hand, soft and flexible devices, such as pHEMA hydrogel-based electrodes, are of great interest to reduce the mechanical mismatch between the soft brain tissue and stiff devices [258]. In addition, soft wire electrodes composed of silicone, a PEDOT-PEG copolymer and CNT have shown great results in intramuscular recordings [230]. 

**Sensors.** CNT-modified electrodes have been widely used for the detection of biomolecules due to their large active surface area, high conductivity, fast electron transfer kinetics and biocompatibility. They have also been used to provide chemical and electrochemical stability to the overall sensor [40,311]. Furthermore, CNTs are useful for the detection of neurotransmitters because the CNTs enhance the sensitivity and selectivity and have electrocatalytic effects, making them ideal biological sensors [312]. For instance, Samba and co-workers developed electrodes composed of PEDOT and CNT and demonstrated outstanding levels of selectivity and sensitivity in detecting small concentrations as low as 1 μM of dopamine, even in the presence of interfering molecules, such as uric acid and ascorbic acid (AA) [313]. 

CNTs allow both adsorption and covalent bonding, thus CNTs can be functionalized with specific recognition antibodies, thereby increasing the list of detection targets. For example, Kim and co-workers fabricated a densely aligned CNT array using Langmuir–Blodgett techniques for detecting biomarkers of Alzheimer’s disease [314]. The authors managed to attach analyte-specific antibodies via a carbodiimide-assisted covalent conjugation method after UV–ozone treatment of the CNT channels. As well, Son et al. were able to detect the aquaporin-4 (AQP4) antibody expressed in Neuromyelitis optica, a rare disease of the central nervous system that affects the optic nerves and the spinal cord, without pretreatment and using a CNT-based FET functionalized with (AQP4) extracellular loop peptides [315]. The sensor was able to detect the antibody with a detection limit of 1 ng·L^−1^ and was anticipated to be a rapid and simple way to detect this disease.

#### 5.2.2. Electronic Applications

CNT and CP are among the most frequently studied advanced electrode materials for electronic devices due to their high surface area and exceptional electrical and mechanical properties. Furthermore, their combination in composite electrodes for asymmetric supercapacitors offers complementary benefits in terms of improvements in the specific capacitance, energy density, and stability of materials. For instance, we have already mentioned that one of the challenges of CP-based devices is to improve their structural stability and, in electronics, such CP films degrade easily after repeating doping and un-doping of anions during the electrochemical redox process. A facile and effective approach is to coat a CNT network with the CP, thus forming a 3D network film that reduces the structural failure caused by anion intercalation and extraction [8]. This new construct provides multiple directions for electron transfer and enhances the charge capacity. 

**Figure 16:**
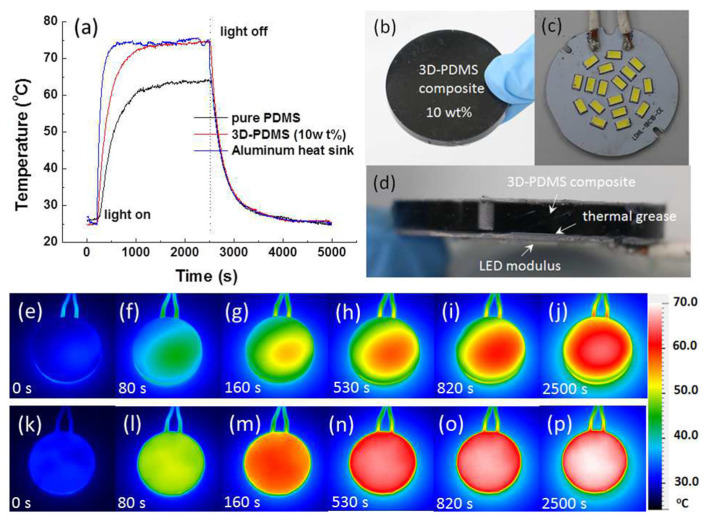
(**a**) Temperature–time profiles of LED modules incorporating Al, pure PDMS, and 3D-PDMS composites as heat sinks. (**b**) Photograph of the 3D-PDMS composite and (**c**) LED modulus. (**d**) Cross sectional image of an LED modulus featuring a polymeric heat sink. (**e**–**p**) Thermal imaging photographs of LED modules featuring (**e**–**j**) pure PDMS and (**k**–**p**) 3D-MWCNT/PDMS composite (10.0 wt%) as heat sinks, taken after various heating times. Reprinted with permission from reference [250]. Copyright (2008) Elsevier.

On the other side, PEDOT:PSS has also been used as a functional surfactant for dispersing CNTs. Upon sonication, PEDOT:PSS covered the surfaces of the MWCNTs, thereby preventing their π-stacking and increasing their dispersion. Furthermore, the CP acted as a thermally conductive bridge that connected the MWCNTs and decreased their thermal resistance, which could potentially be useful for thermal interface materials for thermal management in electronic and photonic applications (Figure 16) [250].

**Figure 17:**
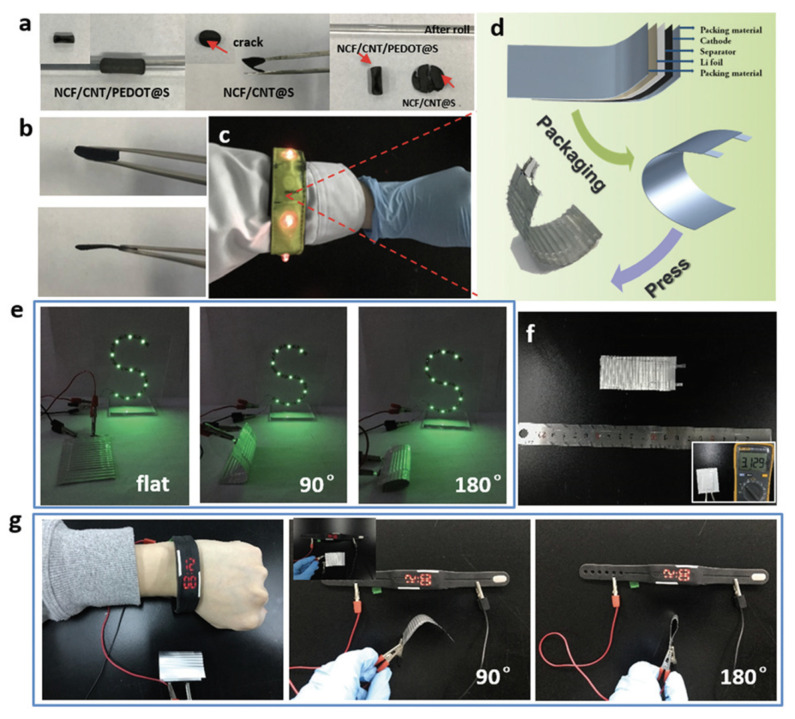
Applications of flexible batteries in charging LEDs, a flexible bracelet, and a smart watch. (**a**) Photographs of the freestanding NCF/CNT/PEDOT@S and NCF/CNT@S electrodes curled around a glass rod. (**b**) Photographs of the freestanding NCF/CNT/PEDOT@S electrodes before (top) and after (below) pressing. (**c**) Powering a flexible bracelet. (**d**) Schematic of the structure of the flexible battery. (**e**) Photographs of the soft-package Li–S battery lighting up 11 LEDs. (**f**) Size of the flexible battery for a bracelet (inset: initial voltage output of the flexible battery). (**g**) Powering a smart bracelet with the flexible NCF/CNT/PEDOT@S battery at 90° and 180° (inset: flat state). Reprinted with permission from reference [241]. Copyright (2018) RSC Pub.

**Energy Storage.** Together with the demand for more efficient, greener, and renewable energy technologies, the need to store this energy has also grown. Furthermore, the increasing interest in wearable devices in the past decade is driving the progress of flexible charge storage devices. Batteries and supercapacitors are the most common devices for electrical energy storage. Lithium-ion batteries (LIBs) have taken over the commercial battery market because of their high energy density and theoretical capacity. In addition, sulfur is environmentally friendly and abundant. Thus, Li–S batteries have become the most promising technology for next generation batteries. The main limitation is the low sulfur loading achieved so far. Therefore, CNT networks on flexible substrates and embedded with CP have been used as hosts for high sulfur loading, while keeping the flexibility and providing high conductive electron pathways [38]. Going a step further, Zhang and co-workers designed 3D electrodes composed of carbon foam/CNTs and filled with PEDOT (NCF/CNT/PEDOT@S), which demonstrated significant achievements at various bending angles after being assembled into multiple flexible Li–S batteries, as shown in Figure 17 [241]. On the other side, the toxic and/or flammable components of LIBs turned raised alarms towards its safety to power wearable devices. Therefore, nonflammable aqueous electrolytes appear to be the ideal replacement and, among them, rechargeable Zn–MnO_2_ batteries are emerging as one of the best candidates, owing to the high abundance and safety of both Zn and MnO_2_, as well as their stable output voltage platform. Again, flexible 3D CNT conductive networks have been highlighted as excellent electron and charge transfer substrates to achieve a high-rate MnO_2_ cathode [238]. The CNT/MnO_2_/PEDOT (denoted as CMOP) composite has been fabricated and showed high capacity, suggesting a step forwards towards next-generation quick charging electronics. 

However, batteries do not reach the ideal properties of high storage capacity, high power and energy densities, or high cyclability. Supercapacitors have thus appeared as advanced electrochemical energy storage devices with the desired characteristics, e.g., high storage capacity, rapid delivery of charge, and long cycle life [316]. With this topic being vast, particular emphasis has been given to PPy and PANi based CNT composites for their use as supercapacitors, due to their good conductivity, high capacitance and low cost [317]. A diverse variety and amount of all-solid state flexible films and papers have been reported, showing a unique microstructure, high electrical conductivity, good wettability, a porous architecture and, overall, higher area capacitance, good rate capability and outstanding cycling stability thanks to the porosity and higher specific area provided by the presence of CNTs [239,318,319]. As well, integrating the device with highly bendable and stretchable polymers, such as polyvinyl alcohol (PVA), yields novel deformable soft supercapacitors with excellent electrochemical performance and flexibility that heralds a new territory of hydrogel-based supercapacitors for portable and wearable devices [9,320]. As well, wire-shaped supercapacitors (WSC) have attracted tremendous attention as they can offer light-weight and small size powering portable electronic systems [321]. Ren et al. designed a facile and scalable process assembling wet-spinning and in situ electrodeposition technique to synthesize MnO2/CNT fibers and PPy/CNT fibers, which were then twisted to fabricate high energy density WSC [322]. Additionally, a similar twisting approach has been used, employing cotton ring spun yarns as the fibrous matrix coated with PPy and CNT [323].

**Photoelectronics.** Even though indium tin oxide (ITO) transparent conductive electrodes (TCE) have been most commonly employed in optoelectronic applications, such as displays, solar cells and touch panels, their brittleness and the increasingly high cost of indium has limited ITO to trendy flexible/wearable devices and demanding low-cost mass production. Several potential replacements have been proposed and, even though there is not a clear winner, two or more materials can combine their respective advantages. CNT-based transparent electrodes gained tremendous attention for application in optoelectronic devices, including solar cells, photodetectors and sensors due to their excellent electrical and mechanical properties. However, one of the major problems for CNT based TCEs, which is also met by other TCEs built up from nanorods/nanowires, is the high contact resistance between connecting CNTs. Among the CPs, PEDOT:PSS has been used in this regard to reduce the contact resistance and the roughness of the CNTs network, together with a highly neutral color transparency around above 80% [324]. Furthermore, as mentioned above, PEDOT:PSS also helps in dispersing CNTs, increasing the concentration of the nanomaterial within the phase separation limits and, thus, increasing the work function of the electrodes [211].

The introduction of CNTs and organic polymers in crystalline silicon hybrid heterojunction solar cells has also gained a great interest in recent years. The main advantages of such heterojunctions include the low-cost fabrication, remarkable power conversion efficiency, simple processing, lightweight, and low-temperature process [325]. Among the various organic/Si solar cells, PEDOT:PSS is the most widely used conducting polymer as the hole selective layer. However, planar PEDOT:PSS/Si devices require enhanced interfacial engineering to reach high power conversion efficiencies (PCE). Therefore, the combination of PEDOT:PSS and CNT into one composite to form coordinate heterojunctions with silicon, not only improves the mechanical strength of the composite film making possible to be used in flexible solar cells, but also helps in maintaining the electrical conductivity [326]. Note that in such hybrid PEDOT:PSS-CNT/n-Si configurations, which are different from the typical designs where an ultrathin polymer layer is sandwiched between the CNT and Si layers, PEDOT:PSS can fill hundreds of nanometer scale pores in the CNT network, which serves as a carrier transport bridge, and both the CNT network and PEDOT:PSS patches contact with the silicon concomitantly. Incorporation of the continuous CNT network with PEDOT:PSS jointly in the as-designed simple and explicit structure has generated synergistic effects, which can make full use of the respective merits and then considerably enhance the PCE. Moreover, a solution of PEDOT:PSS-CNT composite film which can be easily coated over Si or any other flexible substrate using spin coating is, therefore, on the correct path to achieve the desired low-cost and flexible solar cell devices [207]. A vast number of reports have contributed to the race for the finding the highest PCE, and the winning value—for now—is 15.6%, obtained by Yoon and co-workers [327].

#### 5.2.3. Other Applications

Even though the combination of CNT and CP has gained extensive ground in biomaterials and electronics, they have both also shown promising potential in other non-related applications. We would like to briefly mention these, given the successful results observed. One of these applications is in gas sensing and the classification of volatile organic compounds (VOC). Since current commercial VOC sensors lack the ability to distinguish between the individual VOCs detected, CNT sensing materials stand out because of their high surface area and feasibility of functionalization with a large variety of specific molecular targets, thus giving rise to a wide range of choices for improved sensing stability and sensitivity towards the next generation of chemoresistive sensor arrays. In this line, Wang and co-workers demonstrated how vertically aligned-CNT had a 10-fold improved sensitivity upon application of a thin conformal layer of the PEDOT coating [328]. Among the unique advantages of the designed sensor, its high signal quality, good reversibility and short response time should be commented on. In another example, Chen et al. fabricated a paper-based optoelectronic sensor (paper-nose) based on CNT and PEDOT:PSS, among other elements, yielding an enhanced sensor with merged colorimetric (optical) and chemiresistive (electronic) properties [329]. 

One last example of a non-biological and non-electronic application of CNT/CP, is as a hydrophobic or oleophilic sorbent. Wu and co-workers have recently showed a superhydrophobic/oleophilic CNT and PPy coated melamine sponge (m-CNT/PPy@MS) as a self-heating sorbent for high viscosity oils [243]. Thanks to the properties provided by the conductive elements, the sponge was able to convert light and electricity to heat, thus making sure that the adsorbent can be tuned to different working environments. Furthermore, the rapid heat generation on the sponge surface reduces the viscosity of the oil and accelerates the absorption rate. For instance, the authors report that under sun illumination (1.0 kW/m^2^) and an applied voltage (8 V), the surface temperature of the m-CNT/PPy@MS can reach 118.6 °C. The work points the potential of these sponges as a new option to attack large-scale oil spill disasters on the sea surface. 

## 6. Conclusions and Future Perspectives

Here, we have seen how the combination of both CNM and CP materials can improve the resulting hybrid behavior in terms of conductivity and mechanophysical properties and, at the same time, produce new properties such as increased thermal characteristics. The synergy of carbon nanomaterials and CP strengthens the nanocomposites, this combination creates a new generation of devices with unique capabilities. More interestingly, even though separately CNM and CP have low processability and dispersibility characteristics, multiple scientists have succeeded in developing new strategies to obtain a good homogeneity and mixture of both materials, thus yielding novel and advanced hybrid composites in multiple architectures, e.g., fibers, films, 3D scaffolds, hydrogels, with an excellent symbiosis between the properties provided by each of the conductive materials. Furthermore, the examples discussed here show huge potential for up-scaling and industrialization in numerous applications. 

In particular, compared to the rapid development of CPs embedded on CNTs and graphene, research on CDs is still in its infancy. CD–CPs have already shown immense potential in nanotechnology for the development of sensors and energy storage facilities. The high solubility of CDs and their easy doping makes them ideal candidates for replacing other carbon-allotropes/CP mixtures. These properties make the production methods of the nanocomposites simpler, due to their homogeneity and good interaction of the polymers and the CDs. However, the nanocomposites’ robustness is not improved as to as great a degree as in the case of graphene or CNTs. Additionally, low toxicity and good biocompatibility allow for its use in biomedical fields—a context that is still underexplored. The formation of heterostructures through the combination of CDs with graphene or CNTs could be a useful strategy for carbon nanomaterials embedded in CP applications, such as fluorescence applications, where other carbon allotropes present an important handicap. Nevertheless, there is still a vast amount to be explored, and some issues should be addressed for further development. For instance, the synthesis of near-infrared emission CDs from low cost and non-poison materials to improve practical bioapplications. Lastly, it is imperative to explore its excitation-wavelength dependent mechanism.

The good coexistence, multiple applications and different properties of graphene embedded in CP have been discussed in this review. However, there is still a long way to go in terms of the frontline investigation of these nanocomposites. On the one hand, the versatility of graphene-based materials still allows for exploring new conductive composites with promising applications where the interfacial interaction plays an important role. The quality of the produced graphene is also a critical factor to improve, including its functionalization and distribution of the functional groups along the surface, e.g., in GO and rGO. On the other hand, there is no universal strategy for the preparation of these nanocomposites; some drawbacks in the properties of graphene, such as the lack of dispersibility and the difficulty of producing high-quality graphene on a large industrial scale, must be taken into account. For that reason, as mentioned above, the introduction of new generation materials, such as transition metal dichalcogenides or CDs, may avoid or solve some of the problems described here; these materials can form new heterostructures/CP composites with a promising fine-tuning ability in the desired application.

Regarding the biomedical applications of CNT, the brilliant interface between CNT and neuronal networks, together with their inherent conductivity, large surface area and functionalization ability, have placed them as an excellent material for multiple biomedical applications: CNT coatings reduce the impedance of metal microelectrodes, thus, improving brain monitoring, recording and stimulation for long periods; functionalization of CNT with specific targets has been used to develop sensing devices to detect brain biomarkers of neurodegenerative diseases, such as Alzheimer’s disease, and hormones indicative of specific malfunctions; as well, similar functionalizations have been performed for drug loading and release purposes. More specifically, the ability of CNTs to change the electrical properties of neurons makes them great candidates for novel tissue engineering of other electroactive cells, in particular, of myocyte cells, that have not yet been fully exploited. Finally, CNTs have also shown great promise in electronics and energy storage. In particular, CNT/CP hybrids may yield mainstream technologies that require stretchable, self-healing, wearable and waterproof electronic devices in the near future, for multifunctional supercapacitors with an extremely long lifespan.

Overall, the large list of combinations and approaches to fabricate new conductive CNM/CP-containing hybrid materials is increasing exponentially every day. However, above all, the challenge is to find an outstanding combination of one of the CNMs with a CP in a determined architecture for each specific combination. The possibilities are almost infinite and, therefore, the game has only just begun.

## Figures and Tables

**Table 1:** Summary of graphene-based materials embedded in conductive polymers.

CP	Graphene Material	Method	Application	Ref
**PANI**	FLG	in situ polymerization	Methodology	[152]
**PANI**	rGO	in situ polymerization	Supercapacitors	[153]
**PANI**	FLG	in situ polymerization	Supercapacitors	[11]
**PANI/PES**	rGO	Phase inversion	Conductive membranes	[166]
**PANI**	rGO	Electrospinning	Nanofibers	[12]
**PANI**	GO/FLG	in situ polymerization	Hybrid flexible papers	[177]
**PANI**	FLG	in situ polymerization	Electrochromic	[178]
**PANI**	GO	in situ polymerization	Flexible electrodes	[161]
**PEDOT**	GO	in situ polymerization	Bio-interfacing	[160]
**PEDOT**	GO	in situ polymerization	Tissue interface	[179]
**PEDOT**	FLG	in situ polymerization	Biosensor	[180]
**PEDOT**	GO	Microwave in situ polymerization	Biosensor	[181]
**PEDOT**	FLG	Electrochemical polymerization	Photocapacitor	[182]
**PEDOT**	G nanoplatelets	Solvent mixing	Sensor	[183]
**PEDOT**	rGO	in situ polymerization	Sensor	[150]
**PPy**	GO	in situ polymerization	Anticorrosive performance	[154]
**PPy**	FLG	Solvent mixing	Corrosion protection	[165]
**PPy**	GO	in situ polymerization	Drug release	[184]
**PPy**	CVDG	Electrochemical deposition	Bioenergy storage	[185]
**PPy**	GO	Electrochemical deposition	Supercapacitors	[176]
**PPy**	FLG	Solvent mixing/capillary force	Supercapacitors	[36]

## Abbreviations

**CD**	carbon dots	**CND**	carbon nanodots
**CNT**	carbon nanotubes	**CP**	conductive polymers
**CQD**	carbon quantum dots	**CVD**	Chemical Vapour Deposition
**DCM**	dichloromethane	**DMF**	dimethylformamide
**DSSC**	Dye-Sensitized Solar Cells	**ES**	Electrospinning
**FLG**	few-layers graphene	**FT-IR**	Fourier-Transform Infrared Spectroscopy
**G**	graphene	**GCE**	Glassy Carbon Electrode
**GO**	graphene oxide	**GPC**	Gel Permeation Chromatography
**GQD**	graphene quantum dots	**HA**	hyaluronic acid
**hfNSC**	human fetal neural stem cells	**hNSC**	human neural stem cells
**ITO**	indium tin oxide	**MALDI**	Matrix-Assisted Laser Desorption/Ionization
**MWCNT**	multi-walled carbon nanotubes	**NIPAM**	*N*-isopropylacrylamide
**PAA**	poly(acrylamide)	**PAN**	polyacrylonitrile
**PANI**	polyaniline	**PDMS**	polydimethylsiloxane
**PEDOT**	poly(3,4-ethylenedioxythiophene)	**PEG**	polyethyleneglycol
**PET**	polyethylene terephthalate	**pHEMA**	poly(2-hydroxyethyl methacrylate)
**PPy**	polypyrrole	**PSS**	poly(styrenesulfonate)
**PVA**	polyvinyl alcohol	**rGO**	reduced graphene oxide
**SEC**	Size Exclusion Chromatography	**SEM**	Scanning Electron Microscopy
**SPCE**	Screen-Printed Carbon Electrode	**SWCNT**	single-walled carbon nanotubes
**TCE**	transparent conductive electrodes	**TEM**	Transmission Electron Microscopy
**TGA**	Thermogravimetric Analysis	**UV-Vis-NIR**	Ultraviolet-Visible-Near-IR Spectroscopy
**VOC**	volatile organic compounds	**VPP**	vapor phase polymerization
**XPS**	X-Ray Photoelectron Spectroscopy	**YM**	Young’s Modulus

## References

[1] Letheby H. (1862). On the production of a blue substance by the electrolysis of sulphate of aniline. J. Chem. Soc..

[2] De Surville R., Jozefowicz M., Yu L.T., Pepichon J., Buvet R. (1968). Electrochemical chains using protolytic organic semiconductors. Electrochim. Acta.

[3] Diaz A.F., Logan J.A. (1980). Electroactive polyaniline films. J. Electroanal. Chem. Interfacial Electrochem..

[4] MacDiarmid A.G., Epstein A.J. (1989). Polyanilines: A novel class of conducting polymers. Faraday Discuss. Chem. Soc..

[5] Macdiarmid A.G., Chiang J.C., Richter A.F., Epstein A.J. (1987). Polyaniline: A new concept in conducting polymers. Synth. Met..

[6] Chiang J.-C., MacDiarmid A.G. (1986). ‘Polyaniline’: Protonic acid doping of the emeraldine form to the metallic regime. Synth. Met..

[7] Wu K., Xu S.-z., Zhou X.-j., Wu H.-x. (2013). Graphene quantum dots enhanced electrochemical performance of polypyrrole as supercapacitor electrode. J. Electrochem..

[8] Xu K., Zhang Q., Hao Z., Tang Y., Wang H., Liu J., Yan H. (2020). Integrated electrochromic supercapacitors with visual energy levels boosted by coating onto carbon nanotube conductive networks. Sol. Energy Mater. Sol. Cells.

[9] Ben J., Song Z., Liu X., Lu W., Li X. (2020). Fabrication and Electrochemical Performance of PVA/CNT/PANI Flexible Films as Electrodes for Supercapacitors. Nanoscale Res. Lett..

[10] Kazemi F., Naghib S.M., Zare Y., Rhee K.Y. (2020). Biosensing Applications of Polyaniline (PANI)-Based Nanocomposites: A Review. Polym. Rev..

[11] Hu L., Tu J., Jiao S., Hou J., Zhu H., Fray D.J. (2012). In situ electrochemical polymerization of a nanorod-PANI–Graphene composite in a reverse micelle electrolyte and its application in a supercapacitor. Phys. Chem. Chem. Phys..

[12] Moayeri A., Ajji A. (2015). Fabrication of polyaniline/poly (ethylene oxide)/non-covalently functionalized graphene nanofibers via electrospinning. Synth. Met..

[13] Chauhan N.P.S., Mozafari M. (2019). Synthetic route of PANI (II): Enzymatic method. Fundamentals and Emerging Applications of Polyaniline.

[14] Chauhan N.P.S., Milan P.B., Kargozar S., Mozafari M. (2019). Synthetic route of PANI (III): Ultrasound-assisted polymerization. Fundamentals and Emerging Applications of Polyaniline.

[15] Zarrintaj P., Saeb M.R. (2019). Synthetic route of polyaniline (IV): Irradiation path. Fundamentals and Emerging Applications of Polyaniline.

[16] Dalmolin C., Canobre S.C., Biaggio S.R., Rocha-Filho R.C., Bocchi N. (2005). Electropolymerization of polyaniline on high surface area carbon substrates. J. Electroanal. Chem..

[17] Chen W.C., Wen T.C., Gopalan A. (2002). Negative capacitance for polyaniline: An analysis via electrochemical impedance spectroscopy. Synth. Met..

[18] Plesu N., Kellenberger A., Mihali M., Vaszilcsin N. (2010). Effect of temperature on the electrochemical synthesis and properties of polyaniline films. J. Non-Cryst. Solids.

[19] Korent A., Soderžnik K.Ž., Šturm S., Rožman K.Ž. (2020). A Correlative Study of Polyaniline Electropolymerization and its Electrochromic Behavior. J. Electrochem. Soc..

[20] Kaitsuka Y., Goto H. (2016). UV Light Induces Dedoping of Polyaniline. Polymers.

[21] Rannou P., Pron A., Nechtschein M. (1999). UV-vis-NIR studies of new PANI/dopant/solvent associations with metallic-like behaviour. Synth. Met..

[22] Ibrahim K.A. (2017). Synthesis and characterization of polyaniline and poly(aniline-co-o-nitroaniline) using vibrational spectroscopy. Arab. J. Chem..

[23] Karaoğlan N., Bindal C. (2018). Synthesis and optical characterization of benzene sulfonic acid doped polyaniline. Eng. Sci. Technol. Int. J..

[24] Šeděnková I., Trchová M., Stejskal J. (2008). Thermal degradation of polyaniline films prepared in solutions of strong and weak acids and in water—FTIR and Raman spectroscopic studies. Polym. Degrad. Stab..

[25] Yang D., Lu W., Goering R., Mattes B.R. (2009). Investigation of polyaniline processibility using GPC/UV-vis analysis. Synth. Met..

[26] Bláha M., Marek F., Morávkova Z., Svoboda J., Brus J., Dybal J., Prokes J., Varga M., Stejskal J. (2019). Role of p-Benzoquinone in the Synthesis of a Conducting Polymer, Polyaniline. ACS Omega.

[27] Angeli A., Pieroni A. (1919). Sopra un nuovo modo di formazione del nero di pirrolo. Gazz. Chim. Ital..

[28] Dall’ Olio A., Dascola G., Varacca V., Bocchi V. (1968). Résonance paramagnétique électronique et conductivité d’un noir d’oxypyrrol électrolitique. CR. Acad. Sci. Paris.

[29] Gardini G.P. (1973). The Oxidation of Monocyclic Pyrroles. Adv. Heterocycl. Chem..

[30] Diaz A.F., Kanazawa K.K., Gardini G.P. (1979). Electrochemical polymerization of pyrrole. J. Chem. Soc. Chem. Commun..

[31] Jain R., Jadon N., Pawaiya A. (2017). Polypyrrole based next generation electrochemical sensors and biosensors: A review. Trends Anal. Chem..

[32] Stejskal J., Trchová M. (2018). Conducting polypyrrole nanotubes: A review. Chem. Pap..

[33] Vernitskaya V.V., Efimov O.N. (1997). Polypyrrole: A conducting polymer; its synthesis, properties and applications. Russ. Chem. Rev..

[34] Maksymiuk K. (2006). Chemical reactivity of polypyrrole and its relevance to polypyrrole based electrochemical sensors. Electroanalysis.

[35] Watanabe A., Tanaka M., Tanaka J. (1981). Electrical and Optical Properties of a Stable Synthetic Metallic Polymer: Polypyrrole. Bull. Chem. Soc. Jpn..

[36] Biswas S., Drzal L.T. (2010). Multilayered nanoarchitecture of graphene nanosheets and polypyrrole nanowires for high performance supercapacitor electrodes. Chem. Mater..

[37] Zhou X., Ma P., Wang A., Yu C., Qian T., Wu S., Shen J. (2015). Dopamine fluorescent sensors based on polypyrrole/graphene quantum dots core/shell hybrids. Biosens. Bioelectron..

[38] Bao L., Yao J., Zhao S., Lu Y., Su Y., Chen L., Zhao C., Wu F. (2020). Densely Packed 3D Corrugated Papery Electrodes as Polysulfide Reservoirs for Lithium-Sulfur Battery with Ultrahigh Volumetric Capacity. ACS Sustain. Chem. Eng..

[39] Lee J.H., Jang Y.J., Kim D.W., Cheruku R., Thogiti S., Ahn K.-S., Kim J.H. (2019). Application of polypyrrole/sodium dodecyl sulfate/carbon nanotube counter electrode for solid-state dye-sensitized solar cells and dye-sensitized solar cells. Chem. Pap..

[40] Duan D., Yang H., Ding Y., Li L., Ma G. (2019). A three-dimensional conductive molecularly imprinted electrochemical sensor based on MOF derived porous carbon/carbon nanotubes composites and prussian blue nanocubes mediated amplification for chiral analysis of cysteine enantiomers. Electrochim. Acta.

[41] Deshmukh K., Ahamed M.B., Deshmukh R.R., Pasha S.K., Bhagat P.R., Chidambaram K. (2017). Biopolymer Composites with High Dielectric Performance: Interface Engineering. Biopolymer Composites in Electronics.

[42] Truong V.T., Ennis B.C., Forsyth M. (1995). Ion exchange, anisotropic structure and thermal stability of polypyrrole films. Synth. Met..

[43] Toshima N., Hara S. (1995). Direct synthesis of conducting polymers from simple monomers. Prog. Polym. Sci..

[44] Rapi S., Bocchi V., Gardini G.P. (1988). Conducting polypyrrole by chemical synthesis in water. Synth. Met..

[45] Kang E.T., Neoh K.G., Ong Y.K., Tan K.L., Tan B.T.G. (1991). X-ray Photoelectron Spectroscopic Studies of Polypyrrole Synthesized with Oxidative Fe(III) Salts. Macromolecules.

[46] Qi G., Huang L., Wang H. (2012). Highly conductive free standing polypyrrole films prepared by freezing interfacial polymerization. Chem. Commun..

[47] Bloor D., Monkman A.P., Stevens G.C., Cheung K.M., Pugh S. (1990). Structure-Property Relationships in Conductive Polymers. Mol. Cryst. Liq. Cryst..

[48] Beck F., Oberst M. (1989). Electrocatalytic deposition and transformation of polypyrrole layers. Synth. Met..

[49] Debiemme-Chouvy C., Tran T.T.M. (2008). An insight into the overoxidation of polypyrrole materials. Electrochem. Commun..

[50] Shiigi H., Kishimoto M., Yakabe H., Deore B., Nagaoka T. (2002). Highly Selective Molecularly Imprinted Overoxidized Polypyrrole Colloids: One-Step Preparation Technique. Anal. Sci..

[51] Chen X., Yu N., Zhang L., Liu Z., Wang Z., Chen Z. (2015). Synthesis of polypyrrole nanoparticles for constructing full-polymer UV/NIR-shielding film. RSC Adv..

[52] Song M.K., Kim Y.T., Kim B.S., Kim J., Char K., Rhee H.W. (2004). Synthesis and characterization of soluble polypyrrole doped with alkylbenzenesulfonic acids. Synth. Met..

[53] Omastová M., Mičušík M. (2012). Polypyrrole coating of inorganic and organic materials by chemical oxidative polymerisation. Chem. Pap..

[54] Liang Y., Goh J.C.-H. (2020). Polypyrrole-Incorporated Conducting Constructs for Tissue Engineering Applications: A Review. Bioelectricity.

[55] Chougule M.A., Pawar S.G., Godse P.R., Mulik R.N., Sen S., Patil V.B. (2011). Synthesis and Characterization of Polypyrrole (PPy) Thin Films. Soft Nanosci. Lett..

[56] Trchová M., Stejskal J. (2018). Resonance Raman Spectroscopy of Conducting Polypyrrole Nanotubes: Disordered Surface versus Ordered Body. J. Phys. Chem. A.

[57] Bufon C.C.B., Vollmer J., Heinzel T., Espindola P., John H., Heinze J. (2005). Relationship between chain length, disorder, and resistivity in polypyrrole films. J. Phys. Chem. B.

[58] Zotti G., Martina S., Wegner G., Schlüter A.D.D. (1992). Well-defined pyrrole oligomers: Electrochemical and UV/vis studies. Adv. Mater..

[59] Brédas J.L., Silbey R., Boudreaux D.S., Chance R.R. (1983). Chain-Length Dependence of Electronic and Electrochemical Properties of Conjugated Systems: Polyacetylene, Polyphenylene, Polythiophene, and Polypyrrole. J. Am. Chem. Soc..

[60] Mondal S., Sangaranarayanan V.M. (2015). A novel, rapid synthetic protocol for controllable sizes, conductivities and monomer units of soluble polypyrrole. Eur. Polym. J..

[61] Texidó R., Anguera G., Colominas S., Borrós S., Sánchez-García D. (2019). Extended 2,2′-Bipyrroles: New Monomers for Conjugated Polymers with Tailored Processability. Polymers.

[62] Andriukonis E., Ramanaviciene A., Ramanavicius A. (2018). Synthesis of Polypyrrole Induced by [Fe(CN)6]3− and Redox Cycling of [Fe(CN)6]4−/[Fe(CN)6]3−. Polymers.

[63] Armour M., Davies A.G., Upadhyay J., Wassermann A. (1967). Colored electrically conducting polymers from furan, pyrrole, and thiophene. J. Polym. Sci. Part A-1 Polym. Chem..

[64] Tourillon G., Garnier F. (1982). New electrochemically generated organic conducting polymers. J. Electroanal. Chem. Interfacial Electrochem..

[65] McCullough R.D., Lowe R.D., Gu H.B., Yoshino K., Wudl F., Spinelli D. (1992). Enhanced electrical conductivity in regioselectively synthesized poly(3-alkylthiophenes). J. Chem. Soc. Chem. Commun..

[66] Chen T.A., Rieke R.D. (1992). The first regioregular head-to-tail poly(3-hexylthiophene-2,5-diyl) and a regiorandom isopolymer: Nickel versus palladium catalysis of 2(5)-bromo-5(2)-(bromozincio)-3-hexylthiophene polymerization. J. Am. Chem. Soc..

[67] Guillerez S., Bidan G. (1998). New convenient synthesis of highly regioregular poly(3-octylthiophene) based on the Suzuki coupling reaction. Synth. Met..

[68] Wegener P., Feldhues M., Litterer H. (1988). Process for the Preparaton of Thiophene Ethers.

[69] Feldhues M., Kämpf G., Mecklenburg T. (1988). Highly Soluble Electrically Conductive Polymers.

[70] Kämpf G., Feldhues M. (1987). Electrically Conductive Coating Composition, Method for Its Manufacture and Its Use.

[71] Feldhues M., Mecklenburg T., Wegener P., Kämpf G. (1987). Soluble Electrically Conductive Polymers, Method of Producing Them and Their Use.

[72] Feldhues M., Kämpf G., Mecklenburg T. (1987). Modified Electrical Conductive Polymers.

[73] Kämpf G., Feldhues M. (1988). Electrically Conducting Coating Compound, Process for Its Preparation and Its Use/Electrically Conductive Polymers and Their Production.

[74] Heywang G., Jonas F. (1992). Poly(alkylenedioxythiophene)s—New, very stable conducting polymers. Adv. Mater..

[75] Mantione D., del Agua I., Sanchez-Sanchez A., Mecerreyes D. (2017). Poly(3,4-ethylenedioxythiophene) (PEDOT) Derivatives: Innovative Conductive Polymers for Bioelectronics. Polymers.

[76] Ha Y.H., Nikolov N., Pollack S.K., Mastrangelo J., Martin B.D., Shashidhar R. (2004). Towards a Transparent, Highly Conductive Poly(3,4-ethylenedioxythiophene). Adv. Funct. Mater..

[77] Kirchmeyer S., Reuter K. (2005). Scientific importance, properties and growing applications of poly(3,4-ethylenedioxythiophene). J. Mater. Chem..

[78] Zhang X., Lee J.-S., Lee G.S., Cha D.-K., Kim M.J., Yang D.J., Manohar S.K. (2006). Chemical Synthesis of PEDOT Nanotubes. Macromolecules.

[79] Snaith H.J., Kenrick H., Chiesa M., Friend R.H. (2005). Morphological and electronic consequences of modifications to the polymer anode ‘PEDOT:PSS’. Polymer.

[80] Wailes E.M., MacNeill C.M., McCabe E., Levi-Polyachenko N.H. (2016). Shaping PEDOT nanoparticles for use in 3D tissue phantoms. J. Appl. Polym. Sci..

[81] Xia Y., Ouyang J. (2012). Significant different conductivities of the two grades of poly(3,4-ethylenedioxythiophene):Poly(styrenesulfonate), Clevios P and clevios PH1000, arising from different molecular weights. ACS Appl. Mater. Interfaces.

[82] Mantione D., Del Agua I., Schaafsma W., Diez-Garcia J., Castro B., Sardon H., Mecerreyes D. (2016). Poly(3,4-ethylenedioxythiophene):GlycosAminoGlycan Aqueous Dispersions: Toward Electrically Conductive Bioactive Materials for Neural Interfaces. Macromol. Biosci..

[83] Kim S., Sanyoto B., Park W.-T., Kim S., Mandal S., Lim J.-C., Noh Y.-Y., Kim J.-H. (2016). Purification of PEDOT:PSS by Ultrafiltration for Highly Conductive Transparent Electrode of All-Printed Organic Devices. Adv. Mater..

[84] Cho H., Cho W., Kim Y., Lee J.G., Kim J.H. (2018). Influence of residual sodium ions on the structure and properties of poly(3,4-ethylenedioxythiophene):poly(styrenesulfonate). RSC Adv..

[85] Yoo D., Kim J., Kim J.H. (2014). Direct synthesis of highly conductive poly(3,4-ethylenedioxythiophene):Poly(4-styrenesulfonate) (PEDOT:PSS)/graphene composites and their applications in energy harvesting systems. Nano Res..

[86] Wijeratne K., Vagin M., Brooke R., Crispin X. (2017). Poly(3,4-ethylenedioxythiophene)-tosylate (PEDOT-Tos) electrodes in thermogalvanic cells. J. Mater. Chem. A.

[87] Chen S., Petsagkourakis I., Spampinato N., Kuang C., Liu X., Brooke R., Kang E.S.H., Fahlman M., Crispin X., Pavlopoulou E. (2020). Unraveling vertical inhomogeneity in vapour phase polymerized PEDOT:Tos films. J. Mater. Chem. A.

[88] Petsagkourakis I., Kim N., Tybrandt K., Zozoulenko I., Crispin X. (2019). Poly(3,4-ethylenedioxythiophene): Chemical Synthesis, Transport Properties, and Thermoelectric Devices. Adv. Electron. Mater..

[89] Seki Y., Takahashi M., Takashiri M. (2019). Effects of different electrolytes and film thicknesses on structural and thermoelectric properties of electropolymerized poly(3,4-ethylenedioxythiophene) films. RSC Adv..

[90] Massonnet N., Carella A., Jaudouin O., Rannou P., Laval G., Celle C., Simonato J.-P.P. (2014). Improvement of the Seebeck coefficient of PEDOT:PSS by chemical reduction combined with a novel method for its transfer using free-standing thin films. J. Mater. Chem. C.

[91] Koromilas N.D., Lainioti G.C., Oikonomou E.K., Bokias G., Kallitsis J.K. (2014). Synthesis and self-association in dilute aqueous solution of hydrophobically modified polycations and polyampholytes based on 4-vinylbenzyl chloride. Eur. Polym. J..

[92] Sriprachuabwong C., Karuwan C., Wisitsorrat A., Phokharatkul D., Lomas T., Sritongkham P., Tuantranont A. (2012). Inkjet-printed graphene-PEDOT:PSS modified screen printed carbon electrode for biochemical sensing. J. Mater. Chem..

[93] Langford E.G., Shaughnessy K.D., Devore T.C., Lawrence D., Constantin C. (2016). Analysis of PEDOT:PSS Films after Sulfuric Acid Treatment on Silicon and Fused Silica using FT-IR and UV-VIS. MRS Adv..

[94] Naithani S., Schaubroeck D., Vercammen Y., Mandamparambil R., Yakimets I., Van Vaeck L., Van Steenberge G. (2013). Excimer laser patterning of PEDOT:PSS thin-films on flexible barrier foils: A surface analysis study. Appl. Surf. Sci..

[95] Casado N., Hernández G., Veloso A., Devaraj S., Mecerreyes D., Armand M. (2016). PEDOT Radical Polymer with Synergetic Redox and Electrical Properties. ACS Macro Lett..

[96] Kumar A., Reynolds J.R. (1996). Soluble alkyl-substituted poly(ethylenedioxythiophenes) as electrochromic materials. Macromolecules.

[97] Chayer M., Faïd K., Leclerc M. (1997). Highly Conducting Water-Soluble Polythiophene Derivatives. Chem. Mater..

[98] Schottland P., Fichet O., Teyssié D., Chevrot C. (1999). Langmuir-Blodgett films of an alkoxy derivative of poly(3,4-ethylenedioxythiophene). Synth. Met..

[99] Kroto H.W., Heath J.R., O’Brien S.C., Curl R.F., Smalley R.E. (1985). C60: Buckminsterfullerene. Nature.

[100] Iijima S. (1991). Helical microtubules of graphitic carbon. Nature.

[101] Iijima S., Ichihashi T. (1993). Single-shell carbon nanotubes of 1-nm diameter. Nature.

[102] Novoselov K.S., Geim A.K., Morozov S.V., Jiang D., Zhang Y., Dubonos S.V., Grigorieva I.V., Firsov A.A. (2004). Electric Field Effect in Atomically Thin Carbon Films. Science.

[103] Xu X., Ray R., Gu Y., Ploehn H.J., Gearheart L., Raker K., Scrivens W.A. (2004). Electrophoretic analysis and purification of fluorescent single-walled carbon nanotube fragments. J. Am. Chem. Soc..

[104] Sun Y.-P., Zhou B., Lin Y., Wang W., Fernando K.A.S., Pathak P., Meziani M.J., Harruff B.A., Wang X., Wang H. (2006). Quantum-Sized Carbon Dots for Bright and Colorful Photoluminescence. J. Am. Chem. Soc..

[105] Baker S.N., Baker G.A. (2010). Luminescent carbon nanodots: Emergent nanolights. Angew. Chem. Int. Ed..

[106] Li X., Rui M., Song J., Shen Z., Zeng H. (2015). Carbon and Graphene Quantum Dots for Optoelectronic and Energy Devices: A Review. Adv. Funct. Mater..

[107] Zheng X.T., Ananthanarayanan A., Luo K.Q., Chen P. (2015). Glowing graphene quantum dots and carbon dots: Properties, syntheses, and biological applications. Small.

[108] Lim S.Y., Shen W., Gao Z. (2015). Carbon quantum dots and their applications. Chem. Soc. Rev..

[109] Cayuela A., Soriano M.L., Carrillo-Carrión C., Valcárcel M. (2016). Semiconductor and carbon-based fluorescent nanodots: The need for consistency. Chem. Commun..

[110] Liu J., Bi H., Cesar Morais P., Zhang X., Zhang F., Hu L. (2017). Room-temperature magnetism in carbon dots and enhanced ferromagnetism in carbon dots-polyaniline nanocomposite. Sci. Rep..

[111] Feast W.J. (1986). Synthesis of conducting polymers. Handbook of Conducting Polymers.

[112] Pal A., Sk M.P., Chattopadhyay A. (2016). Conducting Carbon Dot-Polypyrrole Nanocomposite for Sensitive Detection of Picric acid. ACS Appl. Mater. Interfaces.

[113] Moorthy M., Kumar V.B., Porat Z.E., Gedanken A. (2018). Novel polymerization of aniline and pyrrole by carbon dots. New J. Chem..

[114] Chen L., Guo C.X., Zhang Q., Lei Y., Xie J., Ee S., Guai G., Song Q., Li C.M. (2013). Graphene quantum-dot-doped polypyrrole counter electrode for high-performance dye-sensitized solar cells. ACS Appl. Mater. Interfaces.

[115] Dinari M., Momeni M.M., Goudarzirad M. (2016). Nanocomposite films of polyaniline/graphene quantum dots and its supercapacitor properties. Surf. Eng..

[116] Xie Y., Du H. (2015). Electrochemical capacitance of a carbon quantum dots-polypyrrole/titania nanotube hybrid. RSC Adv..

[117] Punrat E., Maksuk C., Chuanuwatanakul S., Wonsawat W., Chailapakul O. (2016). Polyaniline/graphene quantum dot-modified screen-printed carbon electrode for the rapid determination of Cr(VI) using stopped-flow analysis coupled with voltammetric technique. Talanta.

[118] Dong Y., Shao J., Chen C., Li H., Wang R., Chi Y., Lin X., Chen G. (2012). Blue luminescent graphene quantum dots and graphene oxide prepared by tuning the carbonization degree of citric acid. Carbon.

[119] Devadas B., Imae T. (2018). Effect of Carbon Dots on Conducting Polymers for Energy Storage Applications. ACS Sustain. Chem. Eng..

[120] Alaş M.O., Güngör A., Genç R., Erdem E. (2019). Feeling the power: Robust supercapacitors from nanostructured conductive polymers fostered with Mn2+ and carbon dots. Nanoscale.

[121] Kepić D.P., Marković Z.M., Jovanović S.P., Peruško D.B., Budimir M.D., Holclajtner-Antunović I.D., Pavlović V.B., Todorović Marković B.M. (2014). Preparation of PEDOT:PSS thin films doped with graphene and graphene quantum dots. Synth. Met..

[122] Li H., Yuan J., Zha L., Wang L., Chen H., Che J. (2020). Soft conducting polymer hydrogels in situ doped by sulfonated graphene quantum dots for enhanced electrochemical activity. J. Mater. Sci. Mater. Electron..

[123] Zhou Y., Sharma S.K., Peng Z., Leblanc R.M. (2017). Polymers in Carbon Dots: A Review. Polymers.

[124] Du F.P., Cao N.N., Zhang Y.F., Fu P., Wu Y.G., Lin Z.D., Shi R., Amini A., Cheng C. (2018). PEDOT:PSS/graphene quantum dots films with enhanced thermoelectric properties via strong interfacial interaction and phase separation. Sci. Rep..

[125] Lim H.C., Min S.H., Lee E., Jang J., Kim S.H., Hong J.I. (2015). Self-assembled Poly(3,4-ethylene dioxythiophene):Poly(styrenesulfonate)/Graphene quantum dot organogels for efficient charge transport in photovoltaic devices. ACS Appl. Mater. Interfaces.

[126] Oh W.K., Kwon O.S., Jang J. (2013). Conducting polymer nanomaterials for biomedical applications: Cellular interfacing and biosensing. Polym. Rev..

[127] Nezakati T., Seifalian A., Tan A., Seifalian A.M. (2018). Conductive Polymers: Opportunities and Challenges in Biomedical Applications. Chem. Rev..

[128] Zhao Z., Xie Y. (2017). Enhanced electrochemical performance of carbon quantum dots-polyaniline hybrid. J. Power Sour..

[129] Kausar A. (2019). Polymer/carbon-based quantum dot nanocomposite: Forthcoming materials for technical application. J. Macromol. Sci. Part A.

[130] O’Regan B., Grätzel M. (1991). A low-cost, high-efficiency solar cell based on dy-sensitized colloidad TiO_2_ films. Nature.

[131] Boschloo G. (2019). Improving the performance of dye-sensitized solar cells. Front. Chem..

[132] Randviir E.P., Brownson D.A., Banks C.E. (2014). A decade of graphene research: Production, applications and outlook. Mater. Today.

[133] Lee C., Wei X., Kysar J.W., Hone J. (2008). Measurement of the elastic properties and intrinsic strength of monolayer graphene. Science.

[134] Morozov S., Novoselov K., Katsnelson M., Schedin F., Elias D., Jaszczak J.A., Geim A. (2008). Giant intrinsic carrier mobilities in graphene and its bilayer. Phys. Rev. Lett..

[135] Nair R.R., Blake P., Grigorenko A.N., Novoselov K.S., Booth T.J., Stauber T., Peres N.M., Geim A.K. (2008). Fine structure constant defines visual transparency of graphene. Science.

[136] Balandin A.A., Ghosh S., Bao W., Calizo I., Teweldebrhan D., Miao F., Lau C.N. (2008). Superior thermal conductivity of single-layer graphene. Nano Lett..

[137] Schwierz F. (2010). Graphene transistors. Nat. Nanotechnol..

[138] Raju A.P.A., Lewis A., Derby B., Young R.J., Kinloch I.A., Zan R., Novoselov K.S. (2014). Wide-area strain sensors based upon graphene-polymer composite coatings probed by Raman spectroscopy. Adv. Funct. Mater..

[139] Yoo J.J., Balakrishnan K., Huang J., Meunier V., Sumpter B.G., Srivastava A., Conway M., Mohana Reddy A.L., Yu J., Vajtai R. (2011). Ultrathin planar graphene supercapacitors. Nano Lett..

[140] Ferrari A.C., Bonaccorso F., Fal’Ko V., Novoselov K.S., Roche S., Bøggild P., Borini S., Koppens F.H., Palermo V., Pugno N. (2015). Science and technology roadmap for graphene, related two-dimensional crystals, and hybrid systems. Nanoscale.

[141] Patchkovskii S., John S.T., Yurchenko S.N., Zhechkov L., Heine T., Seifert G. (2005). Graphene nanostructures as tunable storage media for molecular hydrogen. Proc. Natl. Acad. Sci. USA.

[142] Bianco A., Cheng H.-M., Enoki T., Gogotsi Y., Hurt R.H., Koratkar N., Kyotani T., Monthioux M., Park C.R., Tascon J.M. (2013). All in the graphene family–A recommended nomenclature for two-dimensional carbon materials. Carbon.

[143] Wick P., Louw-Gaume A.E., Kucki M., Krug H.F., Kostarelos K., Fadeel B., Dawson K.A., Salvati A., Vázquez E., Ballerini L. (2014). Classification framework for graphene-based materials. Angew. Chem. Int. Ed..

[144] Pumera M. (2011). Graphene in biosensing. Mater. Today.

[145] Kim H., Abdala A.A., Macosko C.W. (2010). Graphene/polymer nanocomposites. Macromolecules.

[146] Liu J., Cui L., Losic D. (2013). Graphene and graphene oxide as new nanocarriers for drug delivery applications. Acta Biomater..

[147] Sarno M., Baldino L., Scudieri C., Cardea S., Ciambelli P., Reverchon E. (2016). Supercritical CO_2_ processing to improve the electrochemical properties of graphene oxide. J. Supercrit. Fluids.

[148] Du J., Cheng H.M. (2012). The fabrication, properties, and uses of graphene/polymer composites. Macromol. Chem. Phys..

[149] Potts J.R., Lee S.H., Alam T.M., An J., Stoller M.D., Piner R.D., Ruoff R.S. (2011). Thermomechanical properties of chemically modified graphene/poly (methyl methacrylate) composites made by in situ polymerization. Carbon.

[150] Yang Y., Li S., Yang W., Yuan W., Xu J., Jiang Y. (2014). In situ polymerization deposition of porous conducting polymer on reduced graphene oxide for gas sensor. ACS Appl. Mater. Interfaces.

[151] Shi Y., Peng L., Ding Y., Zhao Y., Yu G. (2015). Nanostructured conductive polymers for advanced energy storage. Chem. Soc. Rev..

[152] Park J., Yang X., Wickramasinghe D., Sundhoro M., Orbey N., Chow K.-F., Yan M. (2020). Functionalization of pristine graphene for the synthesis of covalent graphene–polyaniline nanocomposite. RSC Adv..

[153] Kumari P., Khawas K., Nandy S., Kuila B.K. (2016). A supramolecular approach to Polyaniline graphene nanohybrid with three dimensional pillar structures for high performing electrochemical supercapacitor applications. Electrochim. Acta.

[154] Zhu Q., Li E., Liu X., Song W., Li Y., Wang X., Liu C. (2020). Epoxy coating with in-situ synthesis of polypyrrole functionalized graphene oxide for enhanced anticorrosive performance. Prog. Org. Coat..

[155] Khan R., Nishina Y. (2020). Grafting conductive polymers on graphene oxide through cross-linker: A stepwise approach. J. Mater. Chem. A.

[156] Bose S., Kuila T., Uddin M.E., Kim N.H., Lau A.K., Lee J.H. (2010). In-situ synthesis and characterization of electrically conductive polypyrrole/graphene nanocomposites. Polymer.

[157] Zhang Q., Dong H., Hu W. (2018). Electrochemical polymerization for two-dimensional conjugated polymers. J. Mater. Chem. C.

[158] Österholm A., Lindfors T., Kauppila J., Damlin P., Kvarnström C. (2012). Electrochemical incorporation of graphene oxide into conducting polymer films. Electrochim. Acta.

[159] Jiang F., Yao Z., Yue R., Du Y., Xu J., Yang P., Wang C. (2012). Electrochemical fabrication of long-term stable Pt-loaded PEDOT/graphene composites for ethanol electrooxidation. Int. J. Hydrog. Energy.

[160] Luo X., Weaver C.L., Tan S., Cui X.T. (2013). Pure graphene oxide doped conducting polymer nanocomposite for bio-interfacing. J. Mat. Chem. B.

[161] Wang D.-W., Li F., Zhao J., Ren W., Chen Z.-G., Tan J., Wu Z.-S., Gentle I., Lu G.Q., Cheng H.-M. (2009). Fabrication of graphene/polyaniline composite paper via in situ anodic electropolymerization for high-performance flexible electrode. ACS Nano.

[162] Sanes J., Sánchez C., Pamies R., Avilés M.-D., Bermúdez M.-D. (2020). Extrusion of polymer nanocomposites with graphene and graphene derivative nanofillers: An overview of recent developments. Materials.

[163] Garzón C., Palza H. (2014). Electrical behavior of polypropylene composites melt mixed with carbon-based particles: Effect of the kind of particle and annealing process. Compos. Sci. Technol..

[164] Zhang H.-B., Zheng W.-G., Yan Q., Yang Y., Wang J.-W., Lu Z.-H., Ji G.-Y., Yu Z.-Z. (2010). Electrically conductive polyethylene terephthalate/graphene nanocomposites prepared by melt compounding. Polymer.

[165] Qiu S., Li W., Zheng W., Zhao H., Wang L. (2017). Synergistic effect of polypyrrole-intercalated graphene for enhanced corrosion protection of aqueous coating in 3.5% NaCl solution. ACS Appl. Mater. Interfaces.

[166] Subtil E.L., Goncalves J., Lemos H.G., Venancio E.C., Mierzwa J.C., de Souza J.d.S., Alves W., Le-Clech P. (2020). Preparation and characterization of a new composite conductive polyethersulfone membrane using polyaniline (PANI) and reduced graphene oxide (rGO). Chem. Eng. J..

[167] Yu J., Kan C.-W. (2018). Review on fabrication of structurally colored fibers by electrospinning. Fibers.

[168] Bhardwaj N., Kundu S.C. (2010). Electrospinning: A fascinating fiber fabrication technique. Biotechnol. Adv..

[169] Barzegar F., Bello A., Fabiane M., Khamlich S., Momodu D., Taghizadeh F., Dangbegnon J., Manyala N. (2015). Preparation and characterization of poly (vinyl alcohol)/graphene nanofibers synthesized by electrospinning. J. Phys. Chem. Solids.

[170] Ceretti E., Ginestra P.S., Ghazinejad M., Fiorentino A., Madou M. (2017). Electrospinning and characterization of polymer–graphene powder scaffolds. Cirp Ann..

[171] Yang W., Ratinac K.R., Ringer S.P., Thordarson P., Gooding J.J., Braet F. (2010). Carbon nanomaterials in biosensors: Should you use nanotubes or graphene?. Angew. Chem. Int. Ed..

[172] Bao Q., Zhang H., Yang J.x., Wang S., Tang D.Y., Jose R., Ramakrishna S., Lim C.T., Loh K.P. (2010). Graphene–polymer nanofiber membrane for ultrafast photonics. Adv. Funct. Mater..

[173] Song J., Gao H., Zhu G., Cao X., Shi X., Wang Y. (2015). The preparation and characterization of polycaprolactone/graphene oxide biocomposite nanofiber scaffolds and their application for directing cell behaviors. Carbon.

[174] Chu C.-Y., Tsai J.-T., Sun C.-L. (2012). Synthesis of PEDOT-modified graphene composite materials as flexible electrodes for energy storage and conversion applications. Int. J. Hydrog. Energy.

[175] Ma J., Zhou X., Ding S., Liu Z. (2017). Solvent evaporation induced self-assembly of graphene foam for thermally conductive polymers. RSC Adv..

[176] Chang H.-H., Chang C.-K., Tsai Y.-C., Liao C.-S. (2012). Electrochemically synthesized graphene/polypyrrole composites and their use in supercapacitor. Carbon.

[177] Yan X., Chen J., Yang J., Xue Q., Miele P. (2010). Fabrication of free-standing, electrochemically active, and biocompatible graphene oxide− polyaniline and graphene− polyaniline hybrid papers. ACS Appl. Mater. Interfaces.

[178] Lyu H. (2020). Triple layer tungsten trioxide, graphene, and polyaniline composite films for combined energy storage and electrochromic applications. Polymers.

[179] Tian H.-C., Liu J.-Q., Wei D.-X., Kang X.-Y., Zhang C., Du J.-C., Yang B., Chen X., Zhu H.-Y., NuLi Y.-N. (2014). Graphene oxide doped conducting polymer nanocomposite film for electrode-tissue interface. Biomaterials.

[180] Ouyang X., Luo L., Ding Y., Liu B., Xu D. (2014). Simultaneous determination of purine and pyrimidine bases in DNA using poly (3, 4-ethylenedioxythiophene)/graphene composite film. J. Electroanal. Chem..

[181] Lei W., Wu L., Huang W., Hao Q., Zhang Y., Xia X. (2014). Microwave-assisted synthesis of hemin–graphene/poly (3, 4-ethylenedioxythiophene) nanocomposite for a biomimetic hydrogen peroxide biosensor. J. Mat. Chem. B.

[182] Armand M., Endres F., MacFarlane D.R., Ohno H., Scrosati B. (2011). Ionic-liquid materials for the electrochemical challenges of the future. Mater. Mater. Sustain. Energy.

[183] Zahid M., Papadopoulou E.L., Athanassiou A., Bayer I.S. (2017). Strain-responsive mercerized conductive cotton fabrics based on PEDOT: PSS/graphene. Mater. Des..

[184] Weaver C.L., LaRosa J.M., Luo X., Cui X.T. (2014). Electrically controlled drug delivery from graphene oxide nanocomposite films. ACS Nano.

[185] Byun K.-E., Choi D.S., Kim E., Seo D.H., Yang H., Seo S., Hong S. (2011). Graphene–polymer hybrid nanostructure-based bioenergy storage device for real-time control of biological motor activity. ACS Nano.

[186] Sanchez V.C., Jachak A., Hurt R.H., Kane A.B. (2012). Biological interactions of graphene-family nanomaterials: An interdisciplinary review. Chem. Res. Toxicol..

[187] Guo Z., Liao N., Zhang M., Xue W. (2018). Theoretical approach to evaluate graphene/PANI composite as highly selective ammonia sensor. Appl. Surf. Sci..

[188] Muthusankar E., Ragupathy D. (2019). Graphene/Poly (aniline-co-diphenylamine) nanohybrid for ultrasensitive electrochemical glucose sensor. Nano-Struct. Nano-Objects.

[189] Chen J., Wang Y., Cao J., Liu Y., Zhou Y., Ouyang J.-H., Jia D. (2017). Facile co-electrodeposition method for high-performance supercapacitor based on reduced graphene oxide/polypyrrole composite film. ACS Appl. Mater. Interfaces.

[190] Lei W., Si W., Xu Y., Gu Z., Hao Q. (2014). Conducting polymer composites with graphene for use in chemical sensors and biosensors. Microchim. Acta.

[191] Kinloch I.A., Suhr J., Lou J., Young R.J., Ajayan P.M. (2018). Composites with carbon nanotubes and graphene: An outlook. Science.

[192] Hamada N., Sawada S.-i., Oshiyama A. (1992). New one-dimensional conductors: Graphitic microtubules. Phys. Rev. Lett..

[193] Mintmire J.W., Dunlap B.I., White C.T. (1992). Are fullerene tubules metallic?. Phys. Rev. Lett..

[194] Walker B.W., Lara R.P., Mogadam E., Yu C.H., Kimball W., Annabi N. (2019). Rational design of microfabricated electroconductive hydrogels for biomedical applications. Prog. Polym. Sci..

[195] Yu Y., Luo Y., Wu H., Jiang K., Li Q., Fan S., Li J., Wang J. (2018). Ultrastretchable carbon nanotube composite electrodes for flexible lithium-ion batteries. Nanoscale.

[196] Senokos E., Ou Y., Torres J.J., Sket F., González C., Marcilla R., Vilatela J.J. (2018). Energy storage in structural composites by introducing CNT fiber/polymer electrolyte interleaves. Sci. Rep..

[197] Breuer O., Sundararaj U. (2004). Big returns from small fibers: A review of polymer/carbon nanotube composites. Polym. Compos..

[198] Tkalya E.E., Ghislandi M., de With G., Koning C.E. (2012). The use of surfactants for dispersing carbon nanotubes and graphene to make conductive nanocomposites. Curr. Opin. Colloid Interface Sci..

[199] Alegret N., Dominguez-Alfaro A., González-Domínguez J.M., Arnaiz B., Cossío U., Bosi S., Vázquez E., Ramos-Cabrer P., Mecerreyes D., Prato M. (2018). Three-Dimensional Conductive Scaffolds as Neural Prostheses Based on Carbon Nanotubes and Polypyrrole. ACS Appl. Mater. Interfaces.

[200] Dominguez-Alfaro A., Alegret N., Arnaiz B., González-Domínguez J.M., Martin-Pacheco A., Cossío U., Porcarelli L., Bosi S., Vázquez E., Mecerreyes D. (2020). Tailored Methodology Based on Vapor Phase Polymerization to Manufacture PEDOT/CNT Scaffolds for Tissue Engineering. ACS Biomater. Sci. Eng..

[201] Wang Q., Yao Q., Chang J., Chen L. (2012). Enhanced thermoelectric properties of CNT/PANI composite nanofibers by highly orienting the arrangement of polymer chains. J. Mater. Chem..

[202] Dominguez-Alfaro A., Gómez I.J., Alegret N., Mecerreyes D., Prato M. (2021). 2D and 3D Immobilization of Carbon Nanomaterials into PEDOT via Electropolymerization of a Functional Bis-EDOT Monomer. Polymers.

[203] Dominguez-Alfaro A., Alegret N., Arnaiz B., Salsamendi M., Mecerreyes D., Prato M. (2020). Toward Spontaneous Neuronal Differentiation of SH-SY5Y Cells Using Novel Three-Dimensional Electropolymerized Conductive Scaffolds. ACS Appl. Mater. Interfaces.

[204] Mahdavi M., Baniassadi M., Baghani M., Dadmun M., Tehrani M. (2015). 3D reconstruction of carbon nanotube networks from neutron scattering experiments. Nanotechnology.

[205] Goshi N., Castagnola E., Vomero M., Gueli C., Cea C., Zucchini E., Bjanes D., Maggiolini E., Moritz C., Kassegne S. (2018). Glassy carbon MEMS for novel origami-styled 3D integrated intracortical and epicortical neural probes. J. Micromech. Microeng..

[206] Wang Q., Liang X., Zhang D., Miao M. (2020). A multifunctional supercapacitor based on 2D nanosheets on a flexible carbon nanotube film. Dalton Trans..

[207] Markose K.K., Jasna M., Subha P.P., Antony A., Jayaraj M.K. (2020). Performance enhancement of organic/Si solar cell using CNT embedded hole selective layer. Sol. Energy.

[208] Tian Y., Wang T., Geng H.-Z., Etihraj A.S., Gu Z.-Z., Jing L.-C., Yuan X.-T., Zhao H., Wen J.-G., Xu Z.-H. (2019). Improved resistance stability of transparent conducting films prepared by PEDOT: PSS hybrid CNTs treated by a two-step method. Mater. Res. Express.

[209] Du Y., Shi Y., Meng Q., Shen S.Z. (2020). Preparation and thermoelectric properties of flexible SWCNT/PEDOT:PSS composite film. Synth. Met..

[210] Chung S.-H., Kim D.H., Kim H., Kim H., Jeong S.W. (2020). Thermoelectric properties of PEDOT: PSS and acid-treated SWCNT composite films. Mater. Today Commun..

[211] Lee T., Kwon W., Park M. (2019). Highly conductive, transparent and metal-free electrodes with a PEDOT:PSS/SWNT bilayer for high-performance organic thin film transistors. Org. Electron..

[212] Zhao H., Geng W., Cao W.-W., Wen J.-G., Wang T., Tian Y., Jing L.-C., Yuan X.-T., Zhu Z.-R., Geng H.-Z. (2020). Highly stable and conductive PEDOT:PSS/GO-SWCNT bilayer transparent conductive films. New J. Chem..

[213] Xu L., Xu J., Yang Y., Mao X., He X., Yang W., Zhao Y., Zhou Y. (2018). A flexible fabric electrode with hierarchical carbon-polymer composite for functional supercapacitors. J. Mater. Sci. Mater. Electron..

[214] Chang Z.-H., Feng D.-Y., Huang Z.-H., Liu X.-X. (2018). Electrochemical deposition of highly loaded polypyrrole on individual carbon nanotubes in carbon nanotube film for supercapacitor. Chem. Eng. J..

[215] Wang T., Jing L.-C., Zhu Q., Ethiraj A.S., Fan X., Liu H., Tian Y., Zhu Z., Meng Z., Geng H.-Z. (2020). Tannic acid modified graphene/CNT three-dimensional conductive network for preparing high-performance transparent flexible heaters. J. Colloid Interface Sci..

[216] Gu Z.-Z., Tian Y., Geng H.-Z., Rhen D.S., Ethiraj A.S., Zhang X., Jing L.-C., Wang T., Xu Z.-H., Yuan X.-T. (2019). Highly conductive sandwich-structured CNT/PEDOT:PSS/CNT transparent conductive films for OLED electrodes. Appl. Nanosci..

[217] Kizildag N., Ucar N. (2016). Electrospinning Functional Polyacrylonitrile Nanofibers with Polyaniline, Carbon Nanotubes, and Silver Nitrate as Additives. Electrospinning—Material, Techniques, and Biomedical Applications.

[218] Im J.S., Yun J., Kim J.G., Bae T.-S., Lee Y.-S. (2012). The effects of carbon nanotube addition and oxyfluorination on the glucose-sensing capabilities of glucose oxidase-coated carbon fiber electrodes. Appl. Surf. Sci..

[219] Manesh K.M., Kim H.T., Santhosh P., Gopalan A.I., Lee K.P. (2008). A novel glucose biosensor based on immobilization of glucose oxidase into multiwall carbon nanotubes-polyelectrolyte-loaded electrospun nanofibrous membrane. Biosens. Bioelectron..

[220] Su Z., Ding J., Wei G. (2014). Electrospinning: A facile technique for fabricating polymeric nanofibers doped with carbon nanotubes and metallic nanoparticles for sensor applications. RSC Adv..

[221] Ayutsede J., Gandhi M., Sukigara S., Ye H., Hsu C.M., Gogotsi Y., Ko F. (2006). Carbon nanotube reinforced Bombyx mori silk nanofibers by the electrospinning process. Biomacromolecules.

[222] Lewitus D.Y., Landers J., Branch J.R., Smith K.L., Callegari G., Kohn J., Neimark A.V. (2011). Biohybrid Carbon Nanotube/Agarose Fibers for Neural Tissue Engineering. Adv. Funct. Mater..

[223] Shao S., Zhou S., Li L., Li J., Luo C., Wang J., Li X., Weng J. (2011). Osteoblast function on electrically conductive electrospun PLA/MWCNTs nanofibers. Biomaterials.

[224] Meng J., Kong H., Han Z., Wang C., Zhu G., Xie S., Xu H. (2009). Enhancement of nanofibrous scaffold of multiwalled carbon nanotubes/polyurethane composite to the fibroblasts growth and biosynthesis. J. Biomed. Mater. Res. Part A.

[225] Yardimci A.I., Aypek H., Ozturk O., Yilmaz S., Ozcivici E., Mese G., Selamet Y. (2019). CNT Incorporated Polyacrilonitrile/Polypyrrole Nanofibers as Keratinocytes Scaffold. J. Biomim. Biomater. Biomed. Eng..

[226] Roh S.-H. (2015). Electricity Generation from Microbial Fuel Cell with Polypyrrole-Coated Carbon Nanofiber Composite. J. Nanosci. Nanotechnol..

[227] Jung H.-Y., Roh S.-H. (2017). Carbon Nanofiber/Polypyrrole Nanocomposite as Anode Material in Microbial Fuel Cells. J. Nanosci. Nanotechnol..

[228] Ahmed A., Jia Y., Huang Y., Khoso N.A., Deb H., Fan Q., Shao J. (2019). Preparation of PVDF-TrFE based electrospun nanofibers decorated with PEDOT-CNT/rGO composites for piezo-electric pressure sensor. J. Mater. Sci. Mater. Electron..

[229] Hazarika A., Deka B.K., Kim D., Kong K., Park Y.-B., Park H.W. (2017). Microwave-synthesized freestanding iron-carbon nanotubes on polyester composites of woven Kevlar fibre and silver nanoparticle-decorated graphene. Sci. Rep..

[230] Zheng X.S., Griffith A.Y., Chang E., Looker M.J., Fisher L.E., Clapsaddle B., Cui X.T. (2020). Evaluation of a conducting elastomeric composite material for intramuscular electrode application. Acta Biomater..

[231] Jie W., Song F., Li X., Li W., Wang R., Jiang Y., Zhao L., Fan Z., Wang J., Liu B. (2017). Enhancing the proliferation of MC3T3-E1 cells on casein phosphopeptide-biofunctionalized 3D reduced-graphene oxide/polypyrrole scaffolds. RSC Adv..

[232] Choi J.S., Park J.S., Kim B., Lee B.-T., Yim J.-H. (2017). In Vitro Biocompatibility of Vapour Phase Polymerised Conductive Scaffolds for Cell Lines. Polymer.

[233] Ravi M., Paramesh V., Kaviya S.R., Anuradha E., Solomon F.P. (2015). 3D Cell Culture Systems: Advantages and Applications. J. Cell. Physiol..

[234] Chen J., Liu B., Gao X., Xu D. (2018). A review of the interfacial characteristics of polymer nanocomposites containing carbon nanotubes. RSC Adv..

[235] Gorain B., Choudhury H., Pandey M., Kesharwani P., Abeer M.M., Tekade R.K., Hussain Z. (2018). Carbon nanotube scaffolds as emerging nanoplatform for myocardial tissue regeneration: A review of recent developments and therapeutic implications. Biomed. Pharmacother..

[236] Yeo L.Y., Friend J.R. (2006). Electrospinning carbon nanotube polymer composite nanofibers. J. Exp. Nanosci..

[237] Li Y., Ye D., Liu W., Shi B., Guo R., Pei H., Xie J. (2017). A three-dimensional core-shell nanostructured composite of polypyrrole wrapped MnO(2)/reduced graphene oxide/carbon nanotube for high performance lithium ion batteries. J. Colloid Interface Sci..

[238] Zhang X., Wu S., Deng S., Wu W., Zeng Y., Xia X., Pan G., Tong Y., Lu X. (2019). 3D CNTs Networks Enable MnO2 Cathodes with High Capacity and Superior Rate Capability for Flexible Rechargeable Zn-MnO2 Batteries. Small Methods.

[239] Jyothibasu J.P., Kuo D.-W., Lee R.-H. (2019). Flexible and freestanding electrodes based on polypyrrole/carbon nanotube/cellulose composites for supercapacitor application. Cellulose.

[240] Liu J., Wen Y., van Aken P.A., Maier J., Yu Y. (2015). In situ reduction and coating of SnS2 nanobelts for free-standing SnS@polypyrrole-nanobelt/carbon-nanotube paper electrodes with superior Li-ion storage. J. Mater. Chem. A.

[241] Zhang M., Amin K., Cheng M., Yuan H., Mao L., Yan W., Wei Z. (2018). A carbon foam-supported high sulfur loading composite as a self-supported cathode for flexible lithium-sulfur batteries. Nanoscale.

[242] Wang Y., Pan X., Chen Y., Wen Q., Lin C., Zheng J., Li W., Xu H., Qi L. (2020). A 3D porous nitrogen-doped carbon nanotube sponge anode modified with polypyrrole and carboxymethyl cellulose for high-performance microbial fuel cells. J. Appl. Electrochem..

[243] Wu X., Lei Y., Li S., Huang J., Teng L., Chen Z., Lai Y. (2021). Photothermal and Joule heating-assisted thermal management sponge for efficient cleanup of highly viscous crude oil. J. Hazard. Mater..

[244] Zhao W., Li Y., Wu S., Wang D., Zhao X., Xu F., Zou M., Zhang H., He X., Cao A. (2016). Highly Stable Carbon Nanotube/Polyaniline Porous Network for Multifunctional Applications. ACS Appl. Mater. Interfaces.

[245] Zhang Y., Zhen Z., Zhang Z., Lao J., Wei J., Wang K., Kang F., Zhu H. (2015). In-situ synthesis of carbon nanotube/graphene composite sponge and its application as compressible supercapacitor electrode. Electrochim. Acta.

[246] Jiang H., Cai X., Qian Y., Zhang C., Zhou L., Liu W., Li B., Lai L., Huang W. (2017). V2O5 embedded in vertically aligned carbon nanotube arrays as free-standing electrodes for flexible supercapacitors. J. Mater. Chem. A.

[247] Giffney T., Xie M., Sartelet M.C., Aw K. (2015). Vapor phase polymerization of PEDOT on silicone rubber as flexible large strain sensor. Aims Mater. Sci..

[248] Iandolo D., Ravichandran A., Liu X., Wen F., Chan J.K., Berggren M., Teoh S.H., Simon D.T. (2016). Development and Characterization of Organic Electronic Scaffolds for Bone Tissue Engineering. Adv. Healthc. Mater..

[249] Tenhaeff W.E., Gleason K.K. (2008). Initiated and Oxidative Chemical Vapor Deposition of Polymeric Thin Films: iCVD and oCVD. Adv. Funct. Mater..

[250] Chang-Jian C.-W., Cho E.-C., Lee K.-C., Huang J.-H., Chen P.-Y., Ho B.-C., Hsiao Y.-S. (2018). Thermally conductive polymeric composites incorporating 3D MWCNT/PEDOT:PSS scaffolds. Compos. Part B Eng..

[251] Carayon I., Gaubert A., Mousli Y., Philippe B. (2020). Electro-responsive hydrogels: Macromolecular and supramolecular approaches in the biomedical field. Biomater. Sci..

[252] Cao H., Yang Y., Qi Y., Li Y., Sun B., Li Y., Cui W., Li J., Li J. (2018). Intraparticle FRET for Enhanced Efficiency of Two-Photon Activated Photodynamic Therapy. Adv. Healthc. Mater..

[253] Adewunmi A.A., Ismail S., Sultan A.S. (2016). Carbon Nanotubes (CNTs) Nanocomposite Hydrogels Developed for Various Applications: A Critical Review. J. Inorg. Organomet. Polym. Mater..

[254] Homenick C.M., Sheardown H., Adronov A. (2010). Reinforcement of collagen with covalently-functionalized single-walled carbon nanotube crosslinkers. J. Mater. Chem..

[255] Yu H., Zhao H., Huang C., Du Y. (2017). Mechanically and Electrically Enhanced CNT-Collagen Hydrogels As Potential Scaffolds for Engineered Cardiac Constructs. ACS Biomater. Sci. Eng..

[256] Kolahchi R., Safari M., Esmailpour M. (2016). Dynamic stability analysis of temperature-dependent functionally graded CNT-reinforced visco-plates resting on orthotropic elastomeric medium. Compos. Struct..

[257] Rehman H.U., Chen Y., Guo Y., Du Q., Zhou J., Guo Y., Duan H., Li H., Liu H. (2016). Stretchable, strong and self-healing hydrogel by oxidized CNT-polymer composite. Compos. Part A Appl. Sci. Manuf..

[258] Castagnola E., Maggiolini E., Ceseracciu L., Ciarpella F., Zucchini E., De Faveri S., Fadiga L., Ricci D. (2016). pHEMA Encapsulated PEDOT-PSS-CNT Microsphere Microelectrodes for Recording Single Unit Activity in the Brain. Front. Neurosci..

[259] Chen C.-R., Qin H., Cong H.-P., Yu S.-H. (2019). A Highly Stretchable and Real-Time Healable Supercapacitor. Adv. Mater..

[260] Deng Z., Guo Y., Zhao X., Ma P.X., Guo B. (2018). Multifunctional Stimuli-Responsive Hydrogels with Self-Healing, High Conductivity, and Rapid Recovery through Host-Guest Interactions. Chem. Mater..

[261] Shin J., Choi E.J., Cho J.H., Cho A.N., Jin Y., Yang K., Song C., Cho S.W. (2017). Three-Dimensional Electroconductive Hyaluronic Acid Hydrogels Incorporated with Carbon Nanotubes and Polypyrrole by Catechol-Mediated Dispersion Enhance Neurogenesis of Human Neural Stem Cells. Biomacromolecules.

[262] MacDonald R.A., Laurenzi B.F., Viswanathan G., Ajayan P.M., Stegemann J.P. (2005). Collagen-carbon nanotube composite materials as scaffolds in tissue engineering. J. Biomed. Mater. Res. A.

[263] Pok S., Vitale F., Eichmann S.L., Benavides O.M., Pasquali M., Jacot J.G. (2014). Biocompatible Carbon Nanotube–Chitosan Scaffold Matching the Electrical Conductivity of the Heart. ACS Nano.

[264] Tosun Z., McFetridge P.S. (2010). A composite SWNT-collagen matrix: Characterization and preliminary assessment as a conductive peripheral nerve regeneration matrix. J. Neural Eng..

[265] Peña B., Bosi S., Aguado B.A., Borin D., Farnsworth N.L., Dobrinskikh E., Rowland T.J., Martinelli V., Jeong M., Taylor M.R.G. (2017). Injectable Carbon Nanotube-Functionalized Reverse Thermal Gel Promotes Cardiomyocytes Survival and Maturation. ACS Appl. Mater. Interfaces.

[266] Raphey V.R., Henna T.K., Nivitha K.P., Mufeedha P., Sabu C., Pramod K. (2019). Advanced biomedical applications of carbon nanotube. Mater. Sci. Eng. C.

[267] Prajapati S.K., Malaiya A., Kesharwani P., Soni D., Jain A. (2020). Biomedical applications and toxicities of carbon nanotubes. Drug Chem. Toxicol..

[268] Hsu J.-H., Yu C. (2020). Sorting-free utilization of semiconducting carbon nanotubes for large thermoelectric responses. Nano Energy.

[269] Yang X., Wang S., Zhuang X., Tomanec O., Zboril R., Yu D.Y.W., Rogach A.L. (2019). Polypyrrole and Carbon Nanotube Co-Composited Titania Anodes with Enhanced Sodium Storage Performance in Ether-Based Electrolyte. Adv. Sustain. Syst..

[270] Rudramurthy G.R., Swamy M.K. (2018). Potential applications of engineered nanoparticles in medicine and biology: An update. J. Biol. Inorg. Chem..

[271] Gorjikhah F., Davaran S., Salehi R., Bakhtiari M., Hasanzadeh A., Panahi Y., Emamverdy M., Akbarzadeh A. (2016). Improving “lab-on-a-chip” techniques using biomedical nanotechnology: A review. Artif. Cells Nanomed. Biotechnol..

[272] Silva G.A. (2006). Neuroscience nanotechnology: Progress, opportunities and challenges. Nat. Rev. Neurosci..

[273] Rauti R., Musto M., Bosi S., Prato M., Ballerini L. (2019). Properties and Behavior of Carbon Nanomaterials when Interfacing Neuronal Cells: How Far Have We Come?. Carbon.

[274] Mattson M.P., Haddon R.C., Rao A.M. (2000). Molecular functionalization of carbon nanotubes and use as substrates for neuronal growth. J. Mol. Neurosci..

[275] Fabbro A., Prato M., Ballerini L. (2013). Carbon Nanotubes in Neuroregeneration and Repair. Adv. Drug Deliv. Rev..

[276] Fabbro A., Bosi S., Ballerini L., Prato M. (2012). Carbon Nanotubes: Artificial Nanomaterials to Engineer Single Neurons and Neuronal Networks. ACS Chem. Neurosci..

[277] Pampaloni N.P., Scaini D., Perissinotto F., Bosi S., Prato M., Ballerini L. (2018). Sculpting neurotransmission during synaptic development by 2D nanostructured interfaces. Nanomed. Nanotechnol. Biol. Med..

[278] Fiorito S., Russier J., Salemme A., Soligo M., Manni L., Krasnowska E., Bonnamy S., Flahaut E., Serafino A., Togna G.I. (2018). Switching on microglia with electro-conductive multi walled carbon nanotubes. Carbon.

[279] Lovat V., Pantarotto D., Lagostena L., Cacciari B., Grandolfo M., Righi M., Spalluto G., Prato M., Ballerini L. (2005). Carbon Nanotube Substrates Boost Neuronal Electrical Signaling. Nano Lett..

[280] Shao H., Li T.T., Zhu R., Xu X.T., Yu J.D., Chen S.F., Song L., Ramakrishna S., Lei Z.G., Ruan Y.W. (2018). Carbon nanotube multilayered nanocomposites as multifunctional substrates for actuating neuronal differentiation and functions of neural stem cells. Biomaterials.

[281] Su W.T., Shih Y.A. (2015). Nanofiber containing carbon nanotubes enhanced PC12 cell proliferation and neuritogenesis by electrical stimulation. Bio-Med. Mater. Eng..

[282] Mazzatenta A., Giugliano M., Campidelli S., Gambazzi L., Businaro L., Markram H., Prato M., Ballerini L. (2007). Interfacing Neurons with Carbon Nanotubes: Electrical Signal Transfer and Synaptic Stimulation in Cultured Brain Circuits. J. Neurosci..

[283] Fabbro A., Cellot G., Prato M., Ballerini L., Schouenborg J., Garwicz M., Danielsen N. (2011). Interfacing neurons with carbon nanotubes: (re) engineering neuronal signaling. Brain Machine Interfaces: Implications for Science, Clinical Practice and Society.

[284] Cellot G., Cilia E., Cipollone S., Rancic V., Sucapane A., Giordani S., Gambazzi L., Markram H., Grandolfo M., Scaini D. (2009). Carbon nanotubes might improve neuronal performance by favouring electrical shortcuts. Nat. Nanotechnol..

[285] Cellot G., Toma F.M., Varley Z.K., Laishram J., Villari A., Quintana M., Cipollone S., Prato M., Ballerini L. (2011). Carbon nanotube scaffolds tune synaptic strength in cultured neural circuits: Novel frontiers in nanomaterial-tissue interactions. J. Neurosci..

[286] Fabbro A., Sucapane A., Toma F.M., Calura E., Rizzetto L., Carrieri C., Roncaglia P., Martinelli V., Scaini D., Masten L. (2013). Adhesion to Carbon Nanotube Conductive Scaffolds Forces Action-Potential Appearance in Immature Rat Spinal Neurons. PLoS ONE.

[287] Fabbro A., Villari A., Laishram J., Scaini D., Toma F.M., Turco A., Prato M., Ballerini L. (2012). Spinal Cord Explants Use Carbon Nanotube Interfaces to Enhance Neurite Outgrowth and To Fortify Synaptic Inputs. ACS Nano.

[288] Lichtenstein M.P., Carretero N.M., Perez E., Pulido-Salgado M., Moral-Vico J., Sola C., Casan-Pastor N., Sunol C. (2018). Biosafety assessment of conducting nanostructured materials by using co-cultures of neurons and astrocytes. Neurotoxicology.

[289] Accardo A., Cirillo C., Lionnet S., Vieu C., Loubinoux I. (2019). Interfacing cells with microengineered scaffolds for neural tissue reconstruction. Brain Res. Bull..

[290] Papadimitriou L., Manganas P., Ranella A., Stratakis E. (2020). Biofabrication for neural tissue engineering applications. Mater. Today Bio.

[291] Roberts M.J., Leach M.K., Bedewy M., Meshot E.R., Copic D., Corey J.M., Hart A.J. (2014). Growth of primary motor neurons on horizontally aligned carbon nanotube thin films and striped patterns. J. Neural Eng..

[292] Cellot G., Lagonegro P., Tarabella G., Scaini D., Fabbri F., Iannotta S., Prato M., Salviati G., Ballerini L. (2015). PEDOT:PSS Interfaces Support the Development of Neuronal Synaptic Networks with Reduced Neuroglia Response In Vitro. Front. Neurosci..

[293] Lee S.J., Zhu W., Nowicki M., Lee G., Heo D.N., Kim J., Zuo Y.Y., Zhang L.G. (2018). 3D printing nano conductive multi-walled carbon nanotube scaffolds for nerve regeneration. J. Neural Eng..

[294] Wu S.Q., Duan B., Lu A., Wang Y.F., Ye Q.F., Zhang L.N. (2017). Biocompatible chitin/carbon nanotubes composite hydrogels as neuronal growth substrates. Carbohydr. Polym..

[295] Liu X.F., Miller A.L., Park S., Waletzki B.E., Terzic A., Yaszemski M.J., Lu L.C. (2016). Covalent crosslinking of graphene oxide and carbon nanotube into hydrogels enhances nerve cell responses. J. Mat. Chem. B.

[296] Rad S.M., Khorasani M.T., Joupari M.D. (2016). Preparation of HMWCNT/PLLA nanocomposite scaffolds for application in nerve tissue engineering and evaluation of their physical, mechanical and cellular activity properties. Polym. Adv. Technol..

[297] Tayi A.S., Pashuck E.T., Newcomb C.J., McClendon M.T., Stupp S.I. (2014). Electrospinning Bioactive Supramolecular Polymers from Water. Biomacromolecules.

[298] Holzwarth J.M., Ma P.X. (2011). 3D nanofibrous scaffolds for tissue engineering. J. Mater. Chem..

[299] Park S.Y., Kang B.S., Hong S. (2013). Improved neural differentiation of human mesenchymal stem cells interfaced with carbon nanotube scaffolds. Nanomedicine.

[300] Hasanzadeh E., Ebrahimi-Barough S., Mirzaei E., Azami M., Tavangar S.M., Mahmoodi N., Basiri A., Ai J. (2019). Preparation of fibrin gel scaffolds containing MWCNT/PU nanofibers for neural tissue engineering. J. Biomed. Mater. Res. Part A.

[301] Balint R., Cassidy N.J., Cartmell S.H. (2012). Electrical Stimulation: A Novel Tool for Tissue Engineering. Tissue Eng. Part B.

[302] Niu X., Rouabhia M., Chiffot N., King M.W., Zhang Z. (2015). An electrically conductive 3D scaffold based on a nonwoven web of poly(L-lactic acid) and conductive poly(3,4-ethylenedioxythiophene). J. Biomed. Mater. Res. A.

[303] Zhang J., Li M., Kang E.-T., Neoh K.G. (2016). Electrical stimulation of adipose-derived mesenchymal stem cells in conductive scaffolds and the roles of voltage-gated ion channels. Acta Biomater..

[304] Inal S., Hama A., Ferro M., Pitsalidis C., Oziat J., Iandolo D., Pappa A.-M., Hadida M., Huerta M., Marchat D. (2017). Conducting Polymer Scaffolds for Hosting and Monitoring 3D Cell Culture. Adv. Biosyst..

[305] Kolarcik C.L., Catt K., Rost E., Albrecht I.N., Bourbeau D., Du Z.H., Kozai T.D.Y., Luo X.L., Weber D.J., Cui X.T. (2015). Evaluation of poly(3,4-ethylenedioxythiophene)/carbon nanotube neural electrode coatings for stimulation in the dorsal root ganglion. J. Neural Eng..

[306] Kumar S., Kim B.S., Song H. (2018). An Integrated Approach of CNT Front-end Amplifier towards Spikes Monitoring for Neuro-prosthetic Diagnosis. Biochip J..

[307] Zhang J., Liu X.J., Xu W.J., Luo W.H., Li M., Chu F.B., Xu L., Cao A.Y., Guan J.S., Tang S.M. (2018). Stretchable Transparent Electrode Arrays for Simultaneous Electrical and Optical Interrogation of Neural Circuits in Vivo. Nano Lett..

[308] Abu-Saude M.J., Morshed B.I. (2015). Patterned Vertical Carbon Nanotube Dry Electrodes for Impedimetric Sensing and Stimulation. IEEE Sens. J..

[309] Su J.Y., Zhang X., Li M.N., Gao T., Wang R., Chai X.Y., Zhang D.G., Zhang X.H., Sui X.H. Insulation of Carbon Nanotube Yarn Electrodes for Intrafascicular Neural Stimulation and Recording. Proceedings of the 9th International IEEE/EMBS Conference on Neural Engineering.

[310] Pan A.I., Lin M.H., Chung H.W., Chen H., Yeh S.R., Chuang Y.J., Chang Y.C., Yew T.R. (2016). Direct-growth carbon nanotubes on 3D structural microelectrodes for electrophysiological recording. Analyst.

[311] Liu J.H., Liu M.L., Bai Y., Zhang J.H., Liu H.W., Zhu W.B. (2020). Recent Progress in Flexible Wearable Sensors for Vital Sign Monitoring. Sensors.

[312] Massicotte G., Carrara S., Di Micheli G., Sawan M. (2016). A CMOS Amperometric System for Multi-Neurotransmitter Detection. IEEE Trans. Biomed. Circuits Syst..

[313] Samba R., Fuchsberger K., Matiychyn I., Epple S., Kiesel L., Stett A., Schuhmann W., Stelzle M. (2014). Application of PEDOT-CNT Microelectrodes for Neurotransmitter Sensing. Electroanalysis.

[314] Kim K., Kim M.J., Kim W., Kim S.Y., Park S., Park C.B. (2020). Clinically accurate diagnosis of Alzheimer’s disease via multiplexed sensing of core biomarkers in human plasma. Nat. Commun..

[315] Son M., Kim D., Park K.S., Hong S., Park T.H. (2016). Detection of aquaporin-4 antibody using aquaporin-4 extracellular loop-based carbon nanotube biosensor for the diagnosis of neuromyelitis optica. Biosens. Bioelectron..

[316] Afzal A., Abuilaiwi F.A., Habib A., Awais M., Waje S.B., Atieh M.A. (2017). Polypyrrole/carbon nanotube supercapacitors: Technological advances and challenges. J. Power Sources.

[317] Singh P., Kumar V., Kalia S., Swart H.C. (2017). Composites Based on Conducting Polymers and Carbon Nanotubes for Supercapacitors. Conducting Polymer Hybrids.

[318] Zeng S., Chen H., Cai F., Kang Y., Chen M., Li Q. (2015). Electrochemical fabrication of carbon nanotube/polyaniline hydrogel film for all-solid-state flexible supercapacitor with high areal capacitance. J. Mater. Chem. A.

[319] Bai Y., Liu R., Li E., Li X., Liu Y., Yuan G. (2019). Graphene/Carbon Nanotube/Bacterial Cellulose assisted supporting for polypyrrole towards flexible supercapacitor applications. J. Alloy. Compd..

[320] Yin B.-S., Zhang S.-W., Ren Q.-Q., Liu C., Ke K., Wang Z.-B. (2017). Elastic soft hydrogel supercapacitor for energy storage. J. Mater. Chem. A.

[321] Song C., Yun J., Keum K., Jeong Y.R., Park H., Lee H., Lee G., Oh S.Y., Ha J.S. (2019). High performance wire-type supercapacitor with Ppy/CNT-ionic liquid/AuNP/carbon fiber electrode and ionic liquid based electrolyte. Carbon.

[322] Ren C., Yan Y., Sun B., Gu B., Chou T.-W. (2020). Wet-spinning assembly and in situ electrodeposition of carbon nanotube-based composite fibers for high energy density wire-shaped asymmetric supercapacitor. J. Colloid Interface Sci..

[323] Hao B., Deng Z., Bi S., Ran J., Cheng D., Luo L., Cai G., Wang X., Tang X. (2020). In situ polymerization of pyrrole on CNT/cotton multifunctional composite yarn for supercapacitors. Ionics.

[324] Hao H.-Y., Dai L., Li Z., Low K.-H. (2016). Enhanced Conductivity and Color Neutrality of Transparent Conductive Electrodes Based on CNT/PEDOT: PSS Composite with a Layer-by-layer Structure. Advanced Material Engineering: Proceedings of the 2015 International Conference on Advanced Material Engineering.

[325] Mbuyise X.G., Arbab E.A.A., Kaviyarasu K., Pellicane G., Maaza M., Mola G.T. (2017). Zinc oxide doped single wall carbon nanotubes in hole transport buffer layer. J. Alloy. Compd..

[326] Fan Q., Zhang Q., Zhou W., Xia X., Yang F., Zhang N., Xiao S., Li K., Gu X., Xiao Z. (2017). Novel approach to enhance efficiency of hybrid silicon-based solar cells via synergistic effects of polymer and carbon nanotube composite film. Nano Energy.

[327] Yoon S., Ha S.R., Moon T., Jeong S.M., Ha T.-J., Choi H., Kang D.-W. (2019). Carbon nanotubes embedded poly(3,4-ethylenedioxythiophene):poly (styrenesulfonate) hybrid hole collector for inverted planar perovskite solar cells. J. Power Sour..

[328] Wang X., Ugur A., Goktas H., Chen N., Wang M., Lachman N., Kalfon-Cohen E., Fang W., Wardle B.L., Gleasont K.K. (2016). Room Temperature Resistive Volatile Organic Compound Sensing Materials Based on a Hybrid Structure of Vertically Aligned Carbon Nanotubes and Conformal oCVD/iCVD Polymer Coatings. ACS Sens..

[329] Chen Y., Owyeung R.E., Sonkusale S.R. (2018). Combined optical and electronic paper-nose for detection of volatile gases. Anal. Chim. Acta.

